# Exploring Size-Controlled Exciton Evolution Using DNA Libraries

**DOI:** 10.1021/jacs.5c21113

**Published:** 2026-02-19

**Authors:** Jeffrey Gorman, Sarah Orsborne, Peter Budden, Akshay Sridhar, Jake L. Greenfield, Daniel G. Congrave, Raj Pandya, Yun Liu, Simon Dowland, Seán Ryan, Hugo Bronstein, Jonathan R. Nitschke, Akshay Rao, Rosana Collepardo-Guevara, Eugen Stulz, Florian Auras, Richard H. Friend

**Affiliations:** 1 Cavendish Laboratory, Department of Physics, 2152 University of Cambridge, Cambridge CB3 0HE, United Kingdom; 2 Department of Chemistry, 3057 Durham University, Durham DH1 3LE, United Kingdom; 3 EaStCHEM School of Chemistry, University of St Andrews, St Andrews, Fife KY16 9ST, United Kingdom; 4 Yusuf Hamied Department of Chemistry, 2152 University of Cambridge, Cambridge CB2 1EG, United Kingdom; 5 Department of Chemistry, 6396 University of Oxford, Oxford OX1 3TA, United Kingdom; 6 Department of Chemistry, University of Warwick, Coventry CV4 7AL, United Kingdom; 7 Institute of High Performance Computing, Agency for Science, Technology and Research, Singapore 138634, Republic of Singapore; 8 Department of Genetics, 2152 University of Cambridge, Cambridge CB2 3EH, United Kingdom; 9 School of Chemistry and Chemical Engineering, 7423 University of Southampton, Southampton SO17 1BJ, United Kingdom; 10 Faculty of Chemistry and Food Chemistry, TUD Dresden University of Technology, Dresden 01069, Germany

## Abstract

To investigate multichromophore phenomena, progress traditionally relies on model covalent dimers for spectroscopic interrogation. Integrating molecular semiconductors into nucleic acid libraries can enable rapid screening of multichromophore phenomena. Here, we report DNA-directed assembly of up to five π-conjugated chromophores that demonstrate charge separation and electronic delocalization phenomena. We integrate a range of porphyrins and perylene diimides (PDIs)—molecular semiconducting materials widely used in organic electronic devices—in DNA, encoding nearest-neighbor assembly through base-sequence programmed hybridization. In this way, we can assemble multicomponent stacks with tailored electronic properties from a central chromophore-DNA library. This allows dimer and multimer production on demand, within hours, from presynthesized DNA-chromophores for spectroscopic analysis. We demonstrate the library’s ability to optimize for charge transfer, computationally prescreening for close π-stacking as a proxy for large orbital overlap and exchange energy. Our modular DNA assembly reveals opportunities for rapid development of simple, bespoke chromophore architectures with stoichiometric chromophore control and ordering.

## Graphical abstract

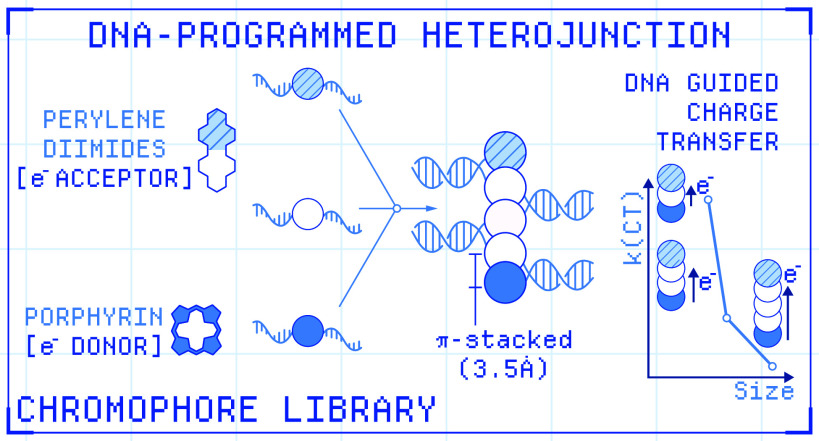

## Introduction

Optoelectronic device optimization begins with molecular design for a specific energetic requirement, followed by synthesis and device engineering to build efficient π-orbital networks. This process relies on “organic semiconducting” molecules, which are π-conjugated chromophores. Their electronic structure can enable exciton and charge transport at the macroscopic device scale. Recent *in silico* molecular design has accelerated iteration and optimization of materials for organic light-emitting diodes (OLEDs), organic field-effect transistors (OFETs), organic photovoltaics (OPVs), and battery technologies.^3–6^ However, functional behavior depends critically on intermolecular organization in condensed phases. Despite many compounds of interest at this modeling stage, condensed-phase implementation often fails to match prediction. One major obstacle is the disordered, aggregated nature within device layers, where uncontrolled intermolecular interactions introduce complex and detrimental electronic couplings that limit efficiency.

A key bottleneck in the optoelectronics discovery pipeline is our inability to control favorable energetics and electronic coupling of designer chromophores in the condensed phase. Chromophores aggregate with minimal control over aggregate size, pairwise spatial separation, and orientation, which are key parameters dictating excited-state evolution. At present, finite self-assembly of simple dimers proves challenging. The self-aggregation of unrestricted chromophores hinders size control of π-stacked molecules, which leads to inefficient trap states from perturbed energy levels.^7–11^ Covalent approaches can confine multiple chromophores into π-stacking arrangements, but the discovery of new, useful compositions can only be achieved by repetitive, synthetic trial and error to generate many structure configurations. Hence, examples of assemblies composed of larger π-stacked heteroaggregates (containing two different chromophores) are rare.^12–14^

In contrast, Nature efficiently optimizes functional biological molecules by mining nucleic acid libraries, iterating through cycles of selection and diversification in parallel to generate high-performing systems. This approach has been mimicked for drug discovery and materials libraries.^15,16^ Minute material quantities are needed for library synthesis and processing. By conjugating abiotic molecules to nucleic acids, non-native materials appropriate DNA’s exceptional assembly, screening, and amplification characteristics.^16–22^

For chromophores attached to DNA, base pairing between complementary single-stranded DNA (ssDNA) is stable, high yielding, and predictable, leading to size-defined structures.^23–25^ Because each member of a chromophore-DNA library can be linked to an encoding nucleic acid strand, they are suitable for plug-and-play combinatorial assembly to screen for excited-state evolution with compositional chromophore control. We had previously used DNA for homoaggregated chromophores that adopt extended stacking structures.^26–28^ However, charge transfer (CTr) at donor–acceptor interfaces requires heteroaggregates, a fundamentally different challenge. Structurally disparate chromophores typically homoaggregate rather than form programmed donor-bridge-acceptor sequences needed to probe how CTr evolves with molecular composition.

In this work, we explore DNA assembly methods to chaperone predetermined copy numbers of chromophores with the goal of CTr within heterojunction structures. We link chromophores and DNA covalently. Conceptually, this instructs self-assembly of nearest-neighbor semiconductors by nucleobase sequence. This unlocks otherwise inaccessible chromophore-sequence programmability along the π-stacking direction, where unencoded-dye conjugates would predominantly self-aggregate. We employ atomistic metadynamics molecular dynamics screening of composite DNA-chromophores, before solid phase oligonucleotide synthesis (SPOS) installs chromophores onto DNA. With this approach, we generate a small library of aqueous-soluble structures. We use a broad range of optical spectroscopic tools, in particular fs and ps transient optical spectroscopy methods, to track the temporal evolution of photogenerated excitons and their subsequent dissociation into separated charges.

## Results

We set out to use DNA as a tool to control structure and electronic coupling between aggregating chromophores. The programmable and selective base pairing could predefine and direct component assembly,^29,30^ while the hydrophilic and charged phosphodiester backbone provides water solubility and a barrier against undesired overaggregation. To build such a large system, we first append our chromophores onto ssDNA.

### Conjugating Chromophore Components to DNA

In order to build the principal library materials, we synthesized a suite of component ssDNAs (Figure 1a) using phosphoramidite and SPOS. To demonstrate this modular approach, we targeted well-characterized chromophores with established excited-state and CTr photophysics: tetra *bay*-phenoxy perylene diimide (**pPDI**), tetra *ortho*-phenyl perylene diimide (**oPDI**),^26^ and porphyrin (**Por**) (Figure 1a). We found that perylene diimides (PDIs)^31,32^ and porphyrins^33,34^ were stable and used these as our model acceptors and donors.

**Figure 1:**
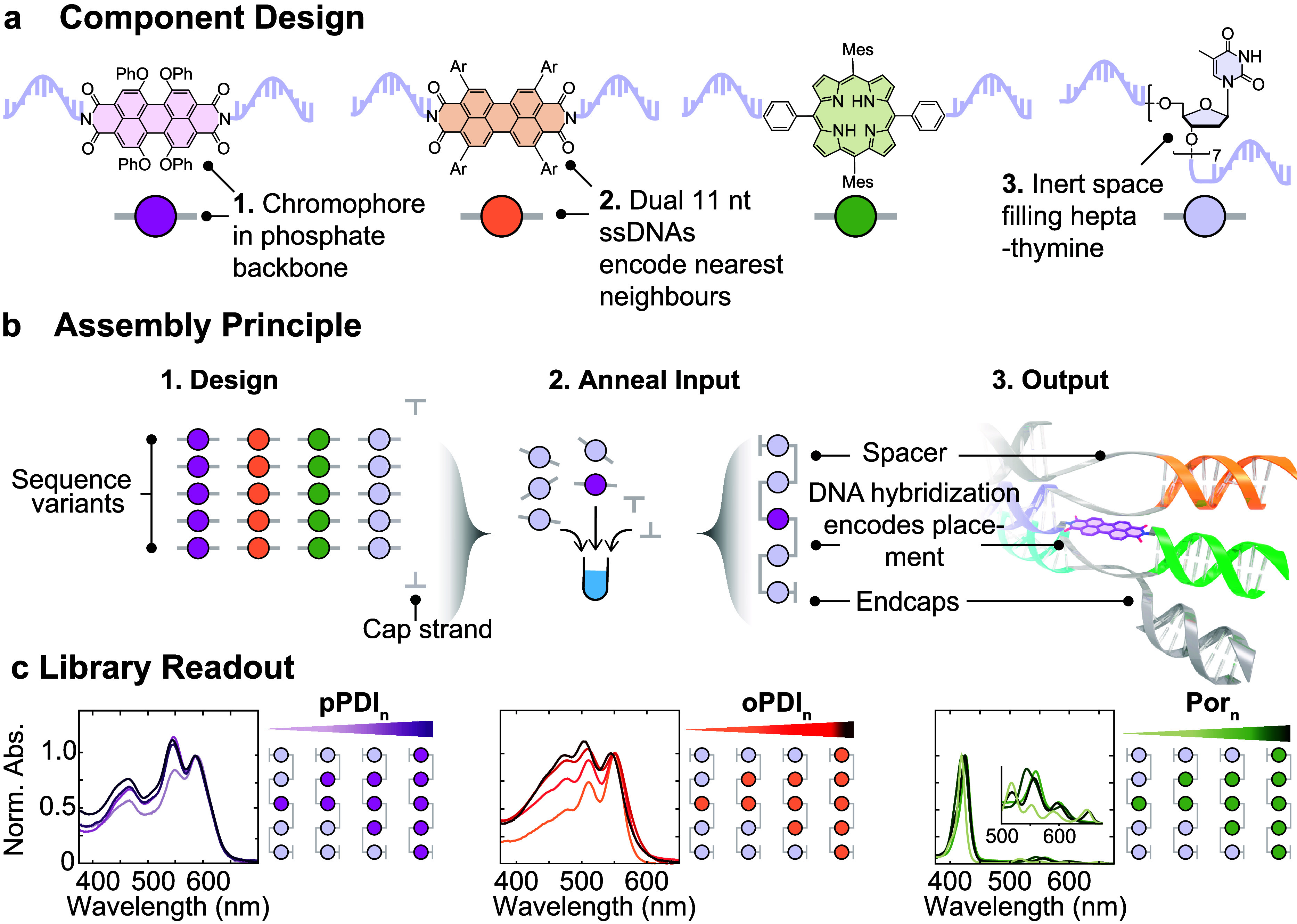
Assembling excitonic chromophores with DNA. (a) Chemical structures of chromophore components appended to two single-stranded DNAs. The two differently substituted perylene diimides (PDIs) and the porphyrin (Por) were the electronically active chromophores, while the heptathymidine (dT)_7_ served as a redox and optically inert spacer. (b) Conceptual basis of this work. The synthetic library was made from five different DNA sequences, with predesigned select base complementarity. All five sequences were modified with our chromophore components at the center. Predesigned π-stacks were assembled by selecting, mixing, and annealing the required library members. Hybridization formed DNA double-helices that simultaneously afforded chromophores self-assembly. End-capping strands hybridized with leftover ssDNA. (c) Optical readout of the generated π-stacks of controlled chromophores copy number. Ar = 4-^*t*^butylphenyl.

Converting chromophores into DNA-compatible phosphoramidites presents unique challenges. SPOS requires hydroxyl groups to couple into the DNA backbone, typically not present on “organic semiconducting” chromophores. This necessitated a protection–deprotection strategy absent from conventional device-oriented syntheses. Hence, Por, pPDI, and oPDI phosphoramidites syntheses (Scheme S1 and S2) were longer (five to six steps) with overall yields of 14–23%.

Por phosphoramidite (23% overall yield, Scheme S1) required temporary ester protection of the hydroxyl during the acid-promoted macrocycle condensation and oxidation. Subsequent base hydrolysis regenerated the free hydroxyls required for SPOS.

The pPDI phosphoramidite synthesis (six steps, 14% yield, Scheme S2) required hydroxyl protection. Tetrachloroperylenedianhydride was first converted to octyl diimides, temporarily masking the imide positions to enable selective phenoxylation of the four chloro-positions. Octyl imides were then hydrolyzed to regenerate the dianhydride before reimidization installed the hydroxyl groups. This imide–anhydride–imide sequence added three steps (half the total route) solely to protect hydroxyls during phenoxylation. The oPDI phosphoramidite (five steps, 14% yield) has been reported previously.^26^

To limit undesired aggregation beyond programmed assemblies, we incorporated heptathymidine (dT)_7_ as a redox and optically inactive component. (dT)_7_ was deployed as a spacer unit on neighboring strands. SPOS enabled direct insertion of the chromophores into the phosphodiester backbone, flanked by two orthogonal 11-nucleotide ssDNA domains to ensure unique sequence recognition and programmability within the assembled architecture.

### Assembling Architectures by Base Pair Programming

To prepare a mix-and-match library approach, all components were inserted into the same master set of five ssDNAs (Figure 1b), simply swapping out which chromophore was in each strand to generate a small library capable of plug-and-play assembly of homo- and heterostructures (SI, Tables S1–S8). We defined the chromophore aggregate size by the number of complementary strands.

All output structures were built from seven interchangeable input ssDNA building blocks, where five ssDNAs deliver an internal chromophore or spacer component. Exchanging a spacer-ssDNA with a chromophore-ssDNA of identical base sequence dictated the number of chromophores in an architecture. For example, monomeric **pPDI**_**2**_ was built by two chromophore components, three spacer components, and two caps (to inhibit sticky-end interactions) in Figure 1b. Larger chromophore structures were built by increasing the number of chromophore components in the place of spacer units, e.g., **pPDI**_**3**_ and **pPDI**_**5**_. The base sequences of the flanking strands of all architectures remained constant; conceptually only the internalized component is exchanged.

Assembly was fully DNA-encoded. Annealing in buffer generated six zigzag-interconnected dsDNA helices, as described previously (Figure 1b).^26^ Library members were mixed equimolarly and annealed in just 1 h, yielding structures immediately ready for optical interrogation. In our hands, this DNA assembly approach could be performed in parallel for different architectures, with a possible upper limit of 384 structures at once using standard DNA equipment (4 × 96-well plates). Non-nucleic **pPDI**, **oPDI**, and **Por** analogues did not self-assemble into the well-defined copy number structures described in Figure 1. ssDNA hybridization enforces a high local concentration of chromophores.^16^ We anticipated quasi-1D chromophore stacks, because confinement by the surrounding dsDNA inhibits significant lateral offsets between chromophores. In this way, envisioned a pipeline of assembly, simulation-informed structure, and optical analysis of the output architecture (Figure 2a–e).

**Figure 2:**
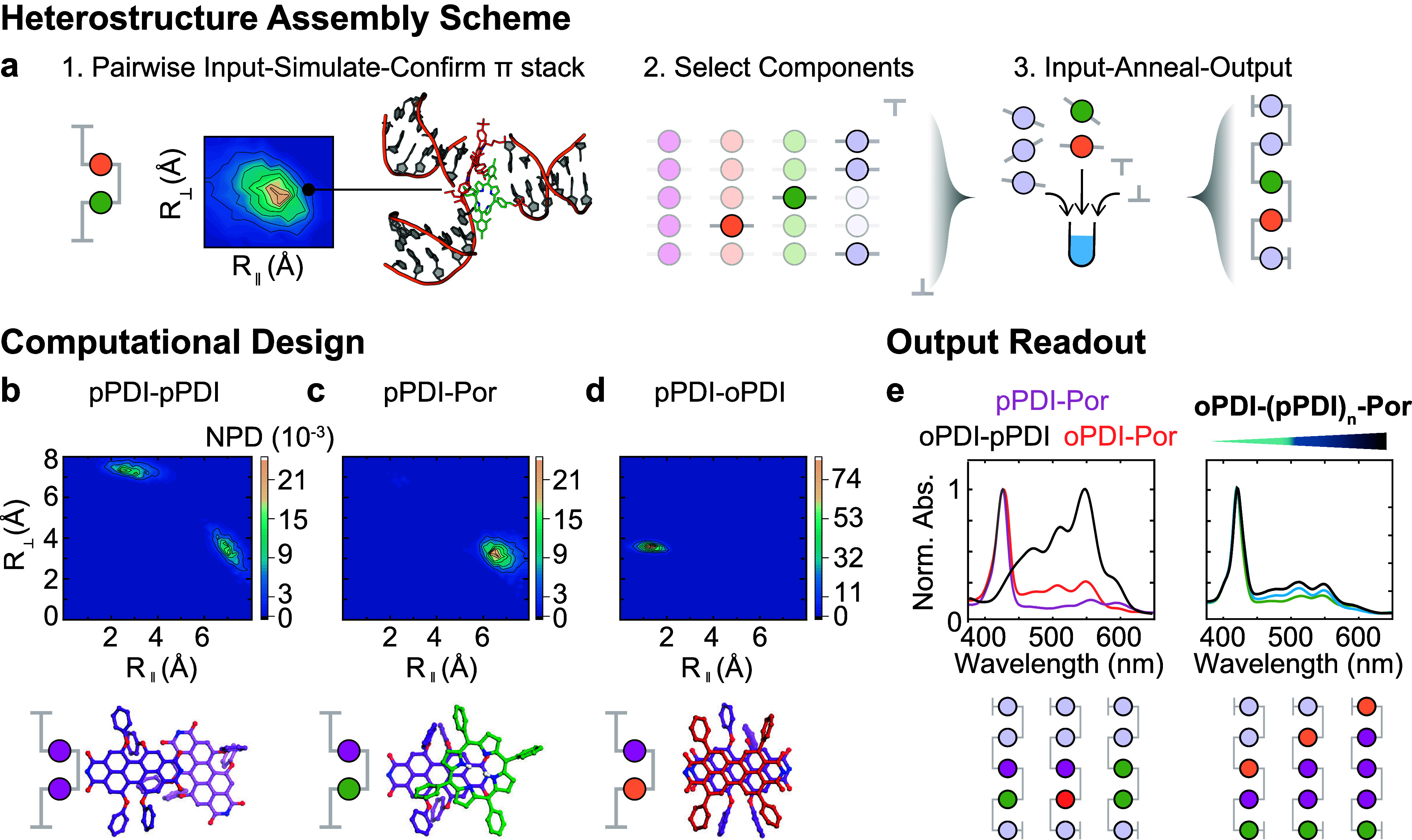
Assembling heterostructured chromophore with DNA. (a) Conceptual scheme for heteroaggregate design. Initially, simplified dimers were generated and simulated to predict favorable orbital overlap for CTr. Combinatorial ssDNA component assemblies were mixed and annealed to output dsDNA-scaffolded heteroaggregates capable of CTr. (b–d) Normalized probability densities (NPDs) of configurations of two chromophore components as a function of orthogonal (*R*_⊥_) and planar (*R*_∥_) center-to-center distances between planes of the chromophore. The sum of all grid element probabilities was normalized to 1 in each experiment. Dominant structures for each interaction are visualized. (e) Optical readout of the generated π-stacks of controlled heteroaggregated chromophore and increasing copy number.

### Modeling of Heterostructures

To investigate whether our component combinatorial approach was generalizable, we sought to develop heterostructures where the different energy levels of each chromophore facilitate an electron donor–acceptor system. With just four components and five master sequences (Figure 1), we could already generate a maximum of 625 different output structure combinations including homo- and heteroaggregated structures. Such large chromophore libraries are typically assayed through singlet exciton emission.^35^ However, the photoluminescence quantum yields (φ_PL_) of monomers **pPDI**_**1**_, **oPDI**_**1**_, and **Por**_**1**_ *vide infra* were low, inhibiting traditional and highly parallelizable 384- or 96-well plate fluorescence readouts. In addition, our work specifically aimed to explore exciton evolution to nonemissive dark states, necessitating a more measured approach to output structure generation that large-scale fluorescent screening.

With the goal of CTr and to address the predicted lack of fluorescent readout in our target structures, we computationally screened for close-packed, pairwise heterodimers (Figure 2a,b). Close π-stacking would provide a screening proxy for large orbital overlap and large exchange energy required for efficiency electron transfer. We performed atomistic molecular dynamics (MD) simulations in combination with a well-tempered metadynamics algorithm^30,36^ for enhanced sampling to characterize the conformational ensembles of various pairs of stacked chromophore-component systems conjugated to dsDNA.^37,38^ Metadynamics simulations were initialized with ≥1.5 nm component separation and proceeded in 2 fs random steps for 100 ns, driven by a history-dependent biasing potential to escape local minima. Given the size of the stacked chromophore-component pairs, prior MD simulations of PDI–DNA base surrogates had shown that the configurations typically converge in <10 ns^39,40^ and well before the 100 ns sampled here. Component and DNA were free to sample a wide range of configurations, including undergoing dsDNA dehybridization and varying of the chromophore separation.

Normalized probability densities (NPDs) as a function of vertical (*R*_⊥_) and lateral (*R*_∥_) displacement between the two π-systems (Figure 2b–d) showed which conformations were populated in the statistical ensembles, with dominant dimer configurations along the *R*_⊥_ axis. During simulation, the chromophore component and DNA were free to adopt any structures, including dsDNA dehybridization and chromophore separation.

Each dimer architecture yielded a preferred cofacial semiconductor structure (∼3.5 Å π–π distance, NPD > 0.018) with dsDNA hybridized (Figure 2b–d and Figures S6–S11). Large *R*_⊥_ separation reflected isolated semiconductors exposed to water, hence were unfavorable (NPD < 0.001). These hydrophobic chromophore interactions partly drove π-stacking. Notably, a single pPDI π-stacked favorably with Por, oPDI, and pPDI components. Hence, we postulated that pPDI could act as a universal structure-bridging component at the center of each of our architectures. **pPDI–pPDI** dimers (Figure 2b) were less preferred and adopt a weaker coupled configuration.^41^ We note that the hierarchy of interactions must be balanced. We had expected that chromophore and DNA stacking interactions would compete with one another. Hence, not all interactions and architectures could assemble with appreciable electronic coupling between chromophores. For example, **oPDI–Por** side-chain steric clash precluded stacking according to our simulations, hence such pairwise interactions were avoided here (Note S1). Additionally, a second **pPDI–pPDI** structure (Figure 2b and Figure S11c) with *R*_⊥_ ∼ 7.5 Å reflected each chromophore component collapsing onto the blunt end of a dsDNA helix. We expect that such a system would only be weakly coupled electronically.

Our modeling suggested that there was sufficient space to cofacially stack all five chromophore components in **oPDI–(pPDI)**_**3**_ **–Por** (Figure S12), while accommodating their bulky dsDNAs. For modeling the entire stacks, we used force-field methods, as MD of larger architectures was computationally too expensive. The pPDI and oPDI pentyl-chains installed to bridge the imide and DNA phosphate backbone were long enough to accommodate the disparate component π–π stacking (∼3.5 Å) and dsDNA repulsions (>1 nm). To summarize, pPDI appeared to stack well with all components, so it was selected as the bridging design unit moving forward experimentally.

### Maintaining Monomeric Excitons

With pPDI established as the principal bridging component in our heterojunction library, we examined its photophysics in its simplest monomer configurations.

To interrogate whether DNA participates electronically in our systems, we tracked the photoluminescence quantum yield (φ_PL_) of each chromophore both off- and on-DNA. The isolated pPDI molecule off-DNA had a high φ_PL_ = 85.7% (Table S18), which decreased to 23.5% upon attachment to DNA (**pPDI**_**1**_), despite appearing disaggregated in its absorption spectrum (Figure 3a). The substantial quenching of the emissive singlet was consistent with the extracted energy level alignments between pPDI and nucleobases (Figure 3b). Previous reports have demonstrated that guanine (G)—the most electron-rich nucleobase—can accept holes from electron-deficient chromophores.^42–45^

**Figure 3:**
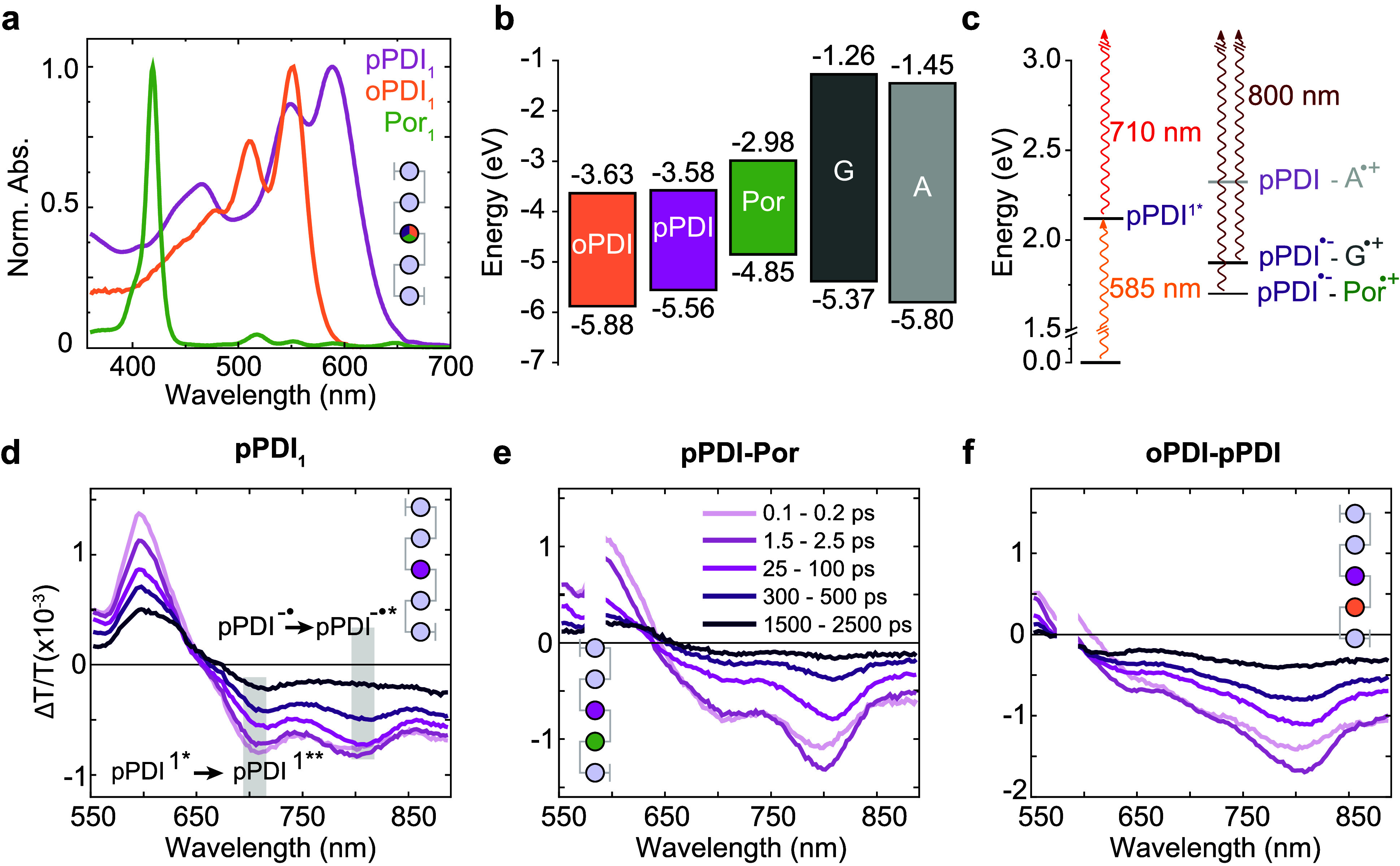
Excited states of DNA-programmed monomers and dimers. (a) Steady-state absorption of monomer architectures. (b) Extracted HOMO–LUMO component energy level alignment. A and G are DNA nucleobases. (c) Neutral and charged state diagram overlaid with fs-TA PIA transitions. (d) fs-TA spectra of monomeric **pPDI** showing excitons and parasitic DNA charge losses. Pump energy density was 25 μJ cm^–2^. (e) fs-TA of heterojunction **pPDI–Por** showing CTr between components. 24 μJ cm^–2^. (f) fs-TA of heterodimer **oPDI–pPDI**. 24.8 μJ cm^–2^. Concentrations of all samples were 10–30 μM in PBS (10 mM phosphate buffer, 200 mM NaCl). λ_ex_ = 585 nm. 575–590 nm removed due to pump scatter. Δ*T*/*T* is the transmittance change of the pump pulse divided by the transmittance without pump excitation.

Hence, we designed the six surrounding nucleobases (corresponding to ∼2.1 nm when fully extended in dsDNA) to be G-free (Tables S1–S17). The favorable energetic alignment between pPDI’s excited state and G’s oxidation potential suggested that this DNA-mediated CTr could occur in **pPDI**_**1**_. In contrast, adenine’s (A) energy levels did not favor singlet quenching of pPDI.

We previously reported oPDI, which is more electron-deficient than pPDI.^26^ In brief, the singlet state of the oPDI chromophore was quenched by both G and A. Off-DNA, oPDI exhibited a low φ_PL_ = 2.5% due to intrinsic nonradiative relaxation pathways mediated by its side chains. DNA conjugation further quenched emission on **oPDI**_**1**_ to φ_PL_ ∼ 0.6%, assigned to hole transfer with both A and G, consistent with the stronger driving force for hole-mediated CTr from this more electron-deficient chromophore.

In contrast, **Por**_**1**_ fully maintained an excitonic state. DNA-assembled **Por**_**1**_ (φ_PL_ = 5.7%) and its off-DNA analogue (φ_PL_ = 5.6%) had the same photoluminescence, as porphyrins are too electron-rich to undergo G-mediated hole transfer, consistent with our band alignment in Figure 3b,c.

To track the fate of **pPDI**_**1**_ excited states, we used vis-NIR transient absorption (TA) spectroscopy (Figure 3d). Nucleobase transitions were insignificant over this spectral window, allowing direct observation of pPDI evolution. We recorded femtosecond TA (fs-TA; Figure 3c) and nanosecond TA (ns-TA; SI, Figure S14a). 585 nm pPDI photoexcitation resulted in positive overlapping ground-state bleach (GSB) and stimulated emission (SE) signals at 550–625 nm that matched ground-state absorption and emission bands, respectively. Photoinduced absorptions (PIAs) caused by transitions from newly excited states appeared negative; these transitions at 650–900 nm were a product of two overlapping bands. We assigned the 710 nm PIA minimum to predominantly pPDI^1^* signal, based on our non-nucleic analogue in CHCl_3_ (Figure S15). We assigned the 800 nm PIA minimum to the radical anion (pPDI^•–^), based on our chemically doped anion spectrum (Figure S16), consistent with earlier reports.^46–48^ Early time pPDI^1^* decay coincided with growth of the pPDI^•–^ signal, assigned to CTr between pPDI and surrounding G bases. Most CTr occurred within our instrument response time, where the pPDI^•–^ PIA signal was present from time zero. pPDI^•–^ decayed rapidly with a lifetime of 390 ± 20 ps by charge recombination (CR). The final excited state was an independent residual pPDI^1^* population that had remained unquenched by DNA (Figure S14a).

CTr was strongly distance dependent;^49^ only geometries with close initial pPDI to G distances underwent CTr on this time scale. These independent, pPDI^1^* and pPDI^•–^ PIAs highlighted some structural heterogeneity in **pPDI**_**1**_. Next, we targeted DNA-encoded heterojunctions formed of two interacting chromophore components.

### Directed CTr through DNA-Assembled Dimers

To investigate CTr between different chromophores, we assembled heterojunctions. Replacing one spacer component in **pPDI**_**1**_ with a second chromophore yielded heterostructures **pPDI–Por** (Figure 3e) and **oPDI–pPDI** (Figure 3f). We expected that the HOMO and LUMO energy alignments in **pPDI–Por** favor intercomponent CTr, while the minimal LUMO offset in **oPDI–pPDI** would likely inhibit it (Figure 3b).

We measured the free energy dependence of these CTr processes by Rehm–Weller analysis. The relative energies of neutral and charged pPDI states were plotted in Figure 3c (see SI Section 9 for further details).^42,44,46^ We estimated that CTr in **pPDI–Por** would thermodynamically favor hole transfer to Por over G (Figure 3c), and the electronic contribution of DNA would be minimal in our heterostructures. The **PDI–Por** φ_PL_ = 1.8% was more quenched compared to **pPDI**_**1**_ (φ_PL_ = 23.5%), indicative of greater CTr when Por was controllable placed in the architecture. Additionally, closer proximity (∼3.5 Å π-stacking) and strong electronic coupling between pPDI and Por likely enable interchromophore CTr to outcompete more distant DNA-mediated pathways. We expected a similar argument to hold for **oPDI–Por**.

### Output Homochromophore Structure and Optical Properties

To support our free energy predictions, we interrogated the excited-state dynamics of dimer architectures **pPDI–Por** and **oPDI–pPDI** using fs-TA (Figure 3e,f) and ns-TA (Figure S14b,c). 585 nm photoexcitation of **pPDI–Por** predominantly excited the pPDI, while optical nucleotide and Por transitions were weak over this range (Figure 3e).^50,51^

To track the fidelity of our design concept, we spectroscopically interrogated a series of larger chromophore homostructures with each component (Figure 1c) using this plug-and-play approach.

The peak normalized absorption spectra of all structures were tracked as a readout for our combinatorial component library.

We first examined assembled monomers **pPDI**_**1**_, **oPDI**_**1**_, and **Por**_**1**_ in a DNA environment. Each architecture was made from seven ssDNA components, but only one strand delivered a chromophore component. Absorption spectra of the monomer-encoded chromophore on DNA in aqueous buffer (Figure 1c) were near-identical to their diluted references in CHCl_3_ (Figure S13), suggesting no dipolar coupling with other components in solution.^52^ This confirmed that the *n* = 1 structures remain isolated (>2 nm apart).

Across the **pPDI**_***n***_ series, we observed excitonic coupling when *n* > 1. Monomer **pPDI**_**1**_ yielded *S*_0_ to *S*_1_ ∼550, 590 nm and *S*_0_ to *S*_2_ 400–525 nm vibronic absorption bands. In contrast, **pPDI**_**2**_ dimerization resulted in a redistribution of oscillator strength from the 0–0 transition toward the 0–1 vibrational band, a result of a complex interplay of charge transfer and dipolar couplings between chromophores interacting in a structure.^7^ These shifts indicated controllable π-stacking interactions consistent with the preprogrammed copy number in our designs.

We observed smaller absorptive differences in **pPDI**_**3**_ and **pPDI**_**5**_, suggesting that π-stacked assembly did not proceed beyond a dimer with a mixture of heterogeneous dimeric and monomeric pPDI components. We speculate that this may be a result of the steric bulk of the four tetraphenoxy groups and a twisted perylene core.

While cofacial π-stacking beyond dimers was incomplete for pPDI_*n*_, the DNA template ensured stoichiometric control. Each architecture contained the programmed number of chromophore units at defined positions along the scaffold, distinguishing our approach from both uncontrolled aggregation and fully ordered covalent assemblies.

We have reported the **oPDI**_***n***_ series previously.^26^ In brief, the flatter perylene core of oPDI, and evolving redistribution in oscillator strength as the chromophore components were assembled, indicated consistent π-stacking interactions out to the full pentamer. Modeling has shown that oPDI aggregates could stack closely, consistent with the stronger effect of aggregation on the optical properties.

**Por**_**1**_ monomer absorption showed typical, sharp *S*_0_ to *S*_2_ Soret (400–500 nm) and *Q*-band (500–675 nm) transitions. Excitonic coupling between components was evident in **Por**_**2**_, with a 9 nm red shift of the Soret and redistributions of the vibronic *Q*-band intensities, indicating some orbital overlap. Stoichiometrically controlled structures **Por**_**3**_ and **Por**_**5**_ showed broader Soret bands, again suggesting a heterogeneous mixture of monomeric and dimeric porphyrins in structure beyond a dimer, similar to **pPDI**_***n***_.

### Heterochromophore Optical Properties

GSB, SE, and PIA band positions in **pPDI–Por** were similar to those seen in **pPDI**_**1**_. 710 nm pPDI^1^* and 800 nm pPDI^•–^ PIAs were again present within the instrument response time. However, the amplitude of the pPDI^•–^ signal was visibly larger at early times in **pPDI–Por** (Figure 3e) compared to **pPDI**_**1**_ (Figure 3d), indicating substantially more radical anion formation from CTr to Por, in contrast to the lower amplitude from CTr to DNA in **pPDI**_1_. Residual 710 nm pPDI^1^* PIA rapidly decayed concomitant with growth of the pPDI^•–^ population. These results were consistent with our favorable **pPDI–Por** energy levels and close-packed heterodimer assembly predictions to yield more CTr than monomeric **pPDI**_**1**_. However, this CTr state was short-lived (τ = 402 ± 15 ps). Hole transport from Por to DNA was thermodynamically uphill, and the CTr state could not spatially separate; hence, CR depleted the pPDI^•–^ signal by 3 ns (Figure S14b).

In contrast, we observed only parasitic CTr to DNA in **oPDI–pPDI**, as the small LUMO offset between components was uncompetitive (Figure 3f). Photoexcitation at 585 nm selectively excited pPDI, producing a broad 590–900 nm PIA centered at 800 nm, assigned to a delocalized radical anion. Anion PIA broadening results from electron delocalization over multiple π-stacked PDIs, as reported previously.^46,53–55^ We speculated that the **oPDI–pPDI** radical anion delocalized over a heterodimer of approximately resonant LUMO energies. This delocalization outcompeted the CTr event, as we observed no time-dependent PIA broadening/sharpening consistent with this process. Delocalization over two molecules stabilizes the radical anion, hence the slower decay of the dimeric anion species in **oPDI–pPDI** (τ = 628 ± 20 ns) compared to the monomer **pPDI**_**1**_ (Figure S14a,b). Additionally, we experimentally confirmed that poor π-stacking and coupling observed in MD simulations translates to limited component CTr in fs-TA for **oPDI–Por** (see the Supporting Information (SI)).

### Programmed Copy Number Control Dictates CTr

Finally, we turned our focus to the target architectures holding all three different chromophore components. Excited-state dynamics were examined using fs-TA (Figure 4a–c) and ns-TA (Figure S14d–f).

**Figure 4:**
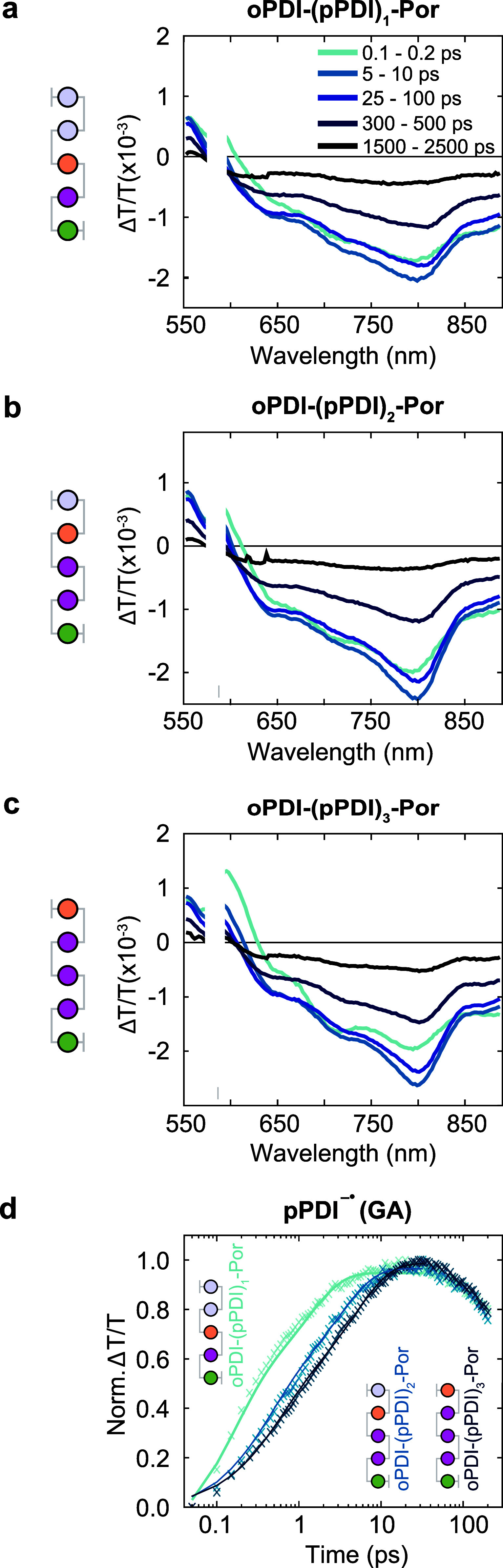
fs-TA of three-component architectures. (a) Trimer **oPDI–(pPDI)**_**1**_ **–Por**. The pump energy density was 24.8 μJ cm^–2^. (b) Tetramer **oPDI–(pPDI)**_**2**_ **–Por**. 17.0 μJ cm^–2^. (c) Pentamer **oPDI–(pPDI)**_**3**_ **–Por**. 21.7 μJ cm^–2^. (d) Growth of pPDI anion extracted using the genetic algorithm (GA, data points) with biexponential fits (solid lines). All samples 10–30 μM, in PBS. λex = 585 nm. 575–590 nm removed due to pump scatter. Δ*T*/*T* is the transmittance change caused by the pump pulse divided by the transmittance without pump excitation.

In the trimer **oPDI–(pPDI)**_**1**_ **–Por**, photophysics were dominated by the stronger **oPDI–pPDI** component interaction (Figure 4a). Similarities between **oPDI–(pPDI)**_**1**_ **–Por** and **oPDI–pPDI** (Figure 3f) spectra indicated that the initial pPDI^1^* was only strongly coupled to the oPDI component in **oPDI–(pPDI)**_**1**_ **–Por**. While MD predicted well-stacked oPDI–pPDI and pPDI–Por dimers (Figure 2b–d), we experimentally confirmed that pPDI did not simultaneously dock to both oPDI and Por. In **oPDI–(pPDI)**_**1**_ **–Por**, the 700 nm pPDI^1^* PIA underwent CTr to G, where the radical anion was delocalized over both PDIs observed as a broad 600–900 nm PIA. We used a genetic algorithm^56^ to extract the individual decay rates for pPDI^1^* and pPDI^•–^ PIAs, which were spectrally overlapped (Figure 4d and Figure S21c). Biexponential growth (τ_1_ = 170 fs, τ_2_ = 1.90 ps) of the anion coincided with pPDI^1^* decay. In the tetramer **oPDI–(pPDI)**_**2**_ **–Por**, hole transfer between pPDI and Por returned as the main CTr decay channel (Figure 4b).

The two pPDI components were each free to engage in optimized structures with oPDI and Por components, respectively. We saw a larger and sharper 800 nm pPDI^•–^ species PIA than **oPDI–(pPDI)**_**1**_ **–Por**. This indicated CTr between pPDI and Por. Again, the amplitude of pPDI^•–^ increased over the first 20 ps. Interestingly, the **oPDI–(pPDI)**_**2**_ **–Por** extracted CTr rate was much slower than **oPDI–(pPDI)**_**1**_ **–Por** (Figure 4d). Biexponential growth (τ_1_ = 370 fs, τ_2_ = 3.08 ps) of the pPDI^•–^ again coincided with pPDI^1^* PIA decay (Figure S21d).

### Origin of Slower CTr in Three-Component Assemblies

We observed a progressive reduction in CTr rate with increasing pPDI stoichiometry across the three-chromophore architectures. We plotted the reduction in CTr rate with increasing size of the three-component systems (Figure 4d). These short, ps time scales could not be assigned to changes arising from DNA structure fluctuations, or pPDI-, oPDI-, and Por-component side-chain rearrangements (>400 ps).^57,58^ Because the structures were essentially stationary on the CTr time scale, our data essentially captured a static ensemble of solution-state geometries. The sub-10 ps time scale for CTr in all systems does indicate a variation in electronic coupling across this heterogeneous distribution. Our absorption data showed that pPDI_*n*_ cofacial aggregation was limited to dimers (Figure 1c), which constrains exciton delocalization effects, leaving structural disorder and hopping as the more plausible contributors.

We have shown that CTr between components only occurred between dimers containing pPDI and Por (Figure 3d,e). The slower CTr rates in larger assemblies likely reflect structural heterogeneity arising from a variation in exciton hopping pathways between pPDI–pPDI sites before reaching the active pPDI–Por pair. Furthermore, CTr slowdown could manifest from a variation in pPDI–Por contact geometries affecting interfacial CTr rates. We speculate that both effects contribute to the ensemble-averaged kinetics. Our MD predictions in Figure 2b show reasonable probability of neighboring pPDI molecules populating structures with large *R*_⊥_ separation. We propose that this slows the CTr rate in larger three-component architectures, a result of incoherent hopping between pPDI sites, before reaching the active pPDI–Por pair heterojunction. In larger architectures (e.g., **oPDI–(pPDI)**_**2**_ **–Por**), the initial 585 nm photoexcitation could sample two pPDI components through a diffusive incoherent hopping mechanism, before reaching the active-pair for CTr. As we encoded more pPDI components into an assembly toward the largest **oPDI–(pPDI)**_**3**_ **–Por**, this incoherent energy transfer regime would take longer on average longer to reach the active pPDI–Por pair. The experimental data in Figure 4d indicated that the initial photoexcitation was substantially localized in our artificial photosynthetic systems. Furthermore, once those excitons reached a pPDI adjacent to Por, the CTr rate depends sensitively on pPDI–Por contact geometry. Our MD simulations (Figure 2c) showed that these pairs populated a distribution of π-stacking configurations. Electronic coupling (and by extension CTr) varies exponentially with separation and orientation offset between pPDI and Por, making CTr rates particularly sensitive to geometric disorder. We speculated that the multicomponent kinetics (Figure 4d) reflected this underlying distribution of both exciton and CTr disorder, which emerged from the structure heterogeneity present in the sample.

## Discussion

This work presents a method for rapid development of bespoke chromophore architectures. Assembly via DNA hybridization provides a route for rapid redesign of candidate structures within hours, enabling significantly faster development cycles than conventional covalent chemistry approaches to multicomponent semiconductor systems.

The large upfront synthetic effort of the three chromophore-DNA building blocks was offset by the modular and rapid DNA assembly, which could then assemble an upper limit of 625 unique chromophore homo/heterostructures in parallel. Here, we limited to 18 structures for detailed spectroscopic analysis. Each chromophore-phosphoramidite building block required 5–6 weeks of dedicated synthesis, likely longer than analogous chromophores for optoelectronic devices due to the additional protection–deprotection steps needed to enable hydroxyl (and DNA) compatibility. However, the phosphoramidites then enable >100 oligonucleotide coupling reactions, each generating unique DNA-chromophore conjugates for the library. The synthetic complexity is comparable to covalent multichromophore systems (e.g., covalent PDI dimers, porphyrin–perylene dyads^10^) with the distinction that each phosphoramidite building block enables extensive architectural diversification rather than yielding a single target structure.

However, expanding this toolkit to new chromophore classes would require repeating phosphoramidite syntheses for each new building block. The method’s throughput advantage is therefore most pronounced when exploring architectural variants of existing building blocks rather than screening chemically diverse chromophore libraries. Our DNA method provides controlled stoichiometry and positioning for fast (<1 h assembly) fs-TA interrogation of different heterostructures, enabling systematic structure–property relationships even for nonemissive states. Importantly, this throughput advantage applies to assembly and characterization of architectures from presynthesized building blocks; the upfront synthesis of new chromophore-phosphoramidites remains the rate-limiting step for expanding chromophore diversity.

Our extracted CTr dynamics provide a molecular ruler for our DNA assembly method. Controlled addition of the pPDI component into our complex, multisemiconductor assembly translated into progressively localized CTr states. These results confirm that our target assemblies systematically increase in size molecule by molecule from **pPDI**_**1**_ through to **oPDI–(pPDI)**_**3**_ **–Por**, encoded by their flanking DNA assembly instructions. Conceptually, these architectures represent primitive photosynthetic reaction centers built from chromophore building blocks typically employed in “organic semiconducting” devices. Our DNA approach shares comparable intermolecular interactions and enables π-wave function delocalization across multiple chromophores.

DNA is also an electronic participant, introducing design constraints that must be managed, but can be predicted. By free energy analysis, we showed that electron-deficient chromophores (pPDI, oPDI) undergo CTr with electron-rich A and G nucleobases on DNA, experimentally confirmed by photoluminescence. Critically, closer π-stacking and stronger energetics in **pPDI–Por** heterostructures mitigate DNA quenching, observed in isolated **pPDI**_**1**_. However, electron-deficient chromophores (n-type materials, e.g., PDIs)—despite their DNA quenching issues—are essential as electron acceptors for solar charge separation, photoredox catalysis, and emerging singlet fission applications. Addressing their parasitic DNA quenching is critical for realizing functional photosystems and quantum information science applications^59,60^ on space-programmable scaffolds. Toward this, mitigation strategies have been disclosed. For example, engineering interchromophore interactions that outcompete DNA interactions are shown here for **pPDI–Por** where favorable energetics enable charge separation despite possible G quenching pathways. Further approaches have used chemically modified purines to reduce CTr driving force,^61^ rigid frameworks minimizing nucleobase orbital overlap,^62^ or longer linkers.

The sequence-encoded assembly method with programmable chromophore stoichiometry opens up greater chemical design space to interrogate phenomena including singlet fission, triplet–triplet annihilation, and symmetry-breaking CTr in the future. Once identified through the DNA assembly method, we envision that those suitable candidates could be transferred to device-compatible covalent chemistry-based architectures.

## Supplementary Material



## Notes

Experimental procedures, synthesis, additional optical spectroscopy, compound characterization data, and copies of NMR spectra (PDF)

## Abbreviations

A: adenine
C: cytosine
CTr: charge transfer
DNA: DNA
dsDNA: double-stranded DNA
fs-TA: femtosecond transient absorption
G: guanine
HOMO: highest occupied molecular orbital
LUMO: lowest unoccupied molecular orbital
MD: molecular dynamics
ns-TA: nanosecond transient absorption
NPD: normalized probability density
oPDI: tetraortho perylene diimide
OFET: organic field-effect transistor
OLED: organic light-emitting diode
OPV: organic photovoltaic
PBS: phosphate-buffered saline
PDI: perylene diimide
φPL: photoluminescence quantum yield
pPDI: tetraphenoxy perylene diimide
Por: porphyrin
SPOS: solid phase oligonucleotide synthesis
ssDNA: single-stranded DNA
T: thymine
TA: transient absorption.
