# Supplementary material for: Exploring Size-Controlled Exciton Evolution Using DNA Libraries
Source: J Am Chem Soc. 2026 Feb 19;148(8):8893–903. doi: 10.1021/jacs.5c21113 (PMC12964420; doi:10.1021/jacs.5c21113)
Supplement: Supplementary file 1 [file ja5c21113_si_001.pdf]

Supporting Information

Exploring Size-Controlled Exciton Evolution Using DNA Libraries

Jeffrey Gorman[1,2*], Sarah Orsborne[1], Peter Budden[1], Akshay Sridhar[1], Jake L. Greenfield[3,4], Daniel G. Congrave[4,5], Raj Pandya[1,6], Yun Liu[1,7], Simon Dowland[1], Seán Ryan[1], Hugo Bronstein[4], Jonathan R. Nitschke[4], Akshay Rao[1], Rosana Collepardo-Guevara[1,4,8*], Eugen Stulz[9*], Florian Auras[1,10*], Richard H. Friend[1*]

[1] Cavendish Laboratory, Department of Physics, University of Cambridge, Cambridge, U.K.
[2] Department of Chemistry, Durham University, Durham, U.K.
[3] EaStCHEM School of Chemistry, University of St Andrews, St Andrews, Fife, KY16 9ST, U.K.
[4] Yusuf Hamied Department of Chemistry, University of Cambridge, Cambridge, U.K.
[5] Department of Chemistry, University of Oxford, OX1 3TA Oxford, U.K.
[6] Department of Chemistry, University of Warwick, Coventry, UK.
[7] Institute of High Performance Computing, Agency for Science, Technology and Research, Singapore, Republic of Singapore.
[8] Department of Genetics, University of Cambridge, Cambridge, U.K.
[9] School of Chemistry and Chemical Engineering, University of Southampton, Southampton, U.K.
[10] Faculty of Chemistry and Food Chemistry, TUD Dresden University of Technology, Dresden, Germany.
* jeffrey.e.gorman@durham.ac.uk, rc597@cam.ac.uk, est@soton.ac.uk, florian.auras@tu-dresden.de, rhf10@cam.ac.uk

Table of contents

1. Methods . . . 2
2. Component-ssDNA Sequences . 3
3. Supplementary Library Spectra . 9
4. Atomistic Metadynamics MD Simulations 21
5. Modelling of Larger Aggregates . 18
6. Monomer Steady-State Spectra . 20
7. ns-Transient Absorption Spectroscopy 22
8. fs-TA and pPDI Radical Anion Spectra 23
9. Rehm-Weller Analysis . . 24
10. Absorption of Dimers . . 26
11. Three-Component Spectra . 26
12. Genetic Algorithm . . 24
13. Supplementary Note 1 . . 29
14. Synthesis . . . 30
15. References . . . 36
16. NMR Spectra . . . 39

Abbreviations

A	adenine
BHT	dibutylhydroxytoluene
C	cytosine
CR	charge recombination
CT	charge transfer
CV	cyclic voltammetry
DBN	1,5-diazabicyclo[4.3.0]non-5-ene
DCM	dichloromethane
DDQ	2,3-dichloro-5,6-dicyano-1,4-benzoquinone
DIPEA	*N*,*N*-diisopropylethylamine
DMAP	4-(dimethylamino)pyridine
DMF	*N*,*N*-dimethylformamide
DMTr	dimethoxytrityl
dsDNA	double stranded deoxyribonucleic acid
E_g	optical bandgap
eq.	equivalents
ESI	electrospray ionisation
ETT	5-(ethylthio)-1*H*-tetrazole
Fc	ferrocene
FT-IR	fourier-transform infrared
G	guanine
GSB	ground state bleach
HOMO	highest occupied molecular orbital
HRMS	high resolution mass spectrometry
IP	ionization potential
ISC	intersystem crossing
LUMO	lowest unoccupied molecular orbital
MALDI-TOF	matrix assisted laser desorption/ionization time of flight
MD	molecular dynamics
NMP	*N*-methyl-2-pyrrolidone
NMR	nuclear magnetic resonance
NPD	normalised probability density
nt	nucleotide
PBS	phosphate buffered saline
PDI	perylene diimide
PIA	photoinduced absorption
PTFE	polytetrafluoroethylene
SE	stimulated emission
SPOS	solid phase oligonucleotide synthesis
ssDNA	single strand deoxyribonucleic acid
T	thymine
TA	transient absorption
TD-DFT	time-dependent density functional theory
TEAA	triethylammonium acetate
TFA	trifluoroacetic acid
THF	tetrahydrofuran
VR	vibrational relaxation
Φ_{PL}	photoluminescence quantum efficiency

1. Methods

Steady-state UV-Vis absorption spectra were recorded on Perkin-Elmer Lambda 950, ThermoFisher NanoDrop ND-1000, or Agilent Cary 400 spectrometers.

Steady-state PL emission spectra were recorded on an Edinburgh FLS980 spectrometer equipped with a photon counting PMT detector.

Photoluminescence Quantum Efficiencies (ϕ_{PL}) measure the ratio of photons emitted to photons absorbed by a sample. To measure the total yield samples are excited inside an integrating sphere. The ϕ_{PL} values were determined by using the method from De Mello *et al.*[1] Temperature and current controlled laser diodes (Thorlabs) were used to generate stable laser beams. These were focused through a small hole onto samples suspended in a Spectralon coated integrating sphere (Newport 819C-SL-5.3) modified with a custom baffle extension. Light from the experiment was collected using an optical fibre connected to a Andor Kymera 328i Spectrometer housing a DU490A-1.7 InGaAs detector.

For **femtosecond transient absorption spectroscopy (fs-TA)**, measurements were taken using a Pharos Amplifier from Light Conversion (1030 nm, 190 fs, 37 kHz). The pump was the ~250fs 530nm output of an ORPHEUS optical parametric amplifier (Light Conversion), while the probe pulses were white light generated by a YAG crystal. Detection was by a JAI Silicon detector, read out by a custom board (Entwicklungsbuero Stresing) at full repetition rate.

For **nanosecond transient absorption (ns-TA)** measurements, the pump was generated by the output of a Ti:Sapphire amplifier system (800 nm, 90 fs, 1 kHz, Solstice Ace), with a Light Conversion TOPAS to generate the desired pump wavelength. The probe was a ~1 ns pulse length supercontinuum laser (LEUKOS Disco STM-1-UV). The probe was split into a probe and reference beam to account for shot-to-shot fluctuations in the probe intensity. The probe and reference beams are focused into an imaging spectrometer (Andor, Shamrock SR 303i) and detected using a pair of linear InGaAs image sensors (Hamamatsu, G11608) driven and read out at the full laser repetition rate by a custom-built board (Entwicklungsbuero Stresing).

Electrochemistry. The experimental setup for cyclic voltammetry (CV) consisted of a polished glassy carbon working electrode, a Pt wire auxiliary electrode and an Ag wire quasi-reference electrode. All measurements were performed under an argon atmosphere and each measurement was referenced to an internal reference, Fc/Fc^+. A 0.1 M $TBAPF_6/CH_2Cl_2$ (TBA = $n\mathrm{Bu}_4\mathrm{N}^+$) electrolyte was employed in the CV measurements.

UV-Vis spectroelectrochemistry was performed using a BioLogic SP-150 potentiostat, an Agilent Cary 60 UV-Vis spectrophotometer, and an optically transparent thin layer electrochemical (OTTLE) cell (purchased from Spectroelectrochemistry Reading). The OTTLE cell comprised of a Pt gauze working electrode, a Pt wire counter electrode and an Ag wire reference electrode. A 0.5 M $TBAPF_6$/DMF electrolyte was employed in these studies. Measurements were performed at 298 K. Samples were prepared in the absence of oxygen and water and sealed in the sample chamber with PTFE plugs.

TD-DFT and Transfer Integral Calculations. The dimer structures without the DNA scaffolding from MD simulation were used without further relaxation. The excited states of the fragment chromophores were computed using time-dependent density functional theory (TDDFT) within the Tamm-Dancoff approximation using ORCA (v4.2).[2,3] To account for the delocalized nature of the molecules, the CAM-B3LYP range-separated functional[4] and def2-TZVP basis set[5] were used in the TDDFT calculations. The transfer integrals of the singlet excitation energy transfer were then computed using the TrEsp method[6,7] as implemented in Multiwfn.[8]

2. Component-ssDNA Sequences

The component-DNA toolbox is constructed such that the base sequences (except for the component) at each position in the stack are identical for all constructs. oPDI strand synthesis and characterization was described previously.[9]

Table S1. Sequences for **pPDI$_1$**.

Position	Strand ID	Sequence 5' → 3'
1	Strand 1	ATA ACT CGG TC
2	Strand 2	GAC CGA GTT AT T TTT TTT TAA CCA GTG GA
3	Strand 3	TCC ACT GGT TA T TTT TTT ATA GGC GGA AC
4	Strand 8	GTT CCG CCT AT (pPDI) ATT CTG TCT GG
5	Strand 5	CCA GAC AGA AT T TTT TTT TTT CCT ACA GG
6	Strand 6	CCT GTA GGA AA T TTT TTT TTA GGT GCA AG
7	Strand 7	CTT GCA CCT AA

Table S2. Sequences for **pPDI$_2$**.

Position	Strand ID	Sequence 5' → 3'
1	Strand 1	ATA ACT CGG TC
2	Strand 2	GAC CGA GTT AT T TTT TTT TAA CCA GTG GA
3	Strand 9	TCC ACT GGT TA (pPDI) ATA GGC GGA AC
4	Strand 8	GTT CCG CCT AT (pPDI) ATT CTG TCT GG
5	Strand 5	CCA GAC AGA AT T TTT TTT TTT CCT ACA GG
6	Strand 6	CCT GTA GGA AA T TTT TTT TTA GGT GCA AG
7	Strand 7	CTT GCA CCT AA

Table S3. Sequences for **pPDI$_3$**.

Position	Strand ID	Sequence 5' → 3'
1	Strand 1	ATA ACT CGG TC
2	Strand 2	GAC CGA GTT AT T TTT TTT TAA CCA GTG GA
3	Strand 9	TCC ACT GGT TA (pPDI) ATA GGC GGA AC
4	Strand 8	GTT CCG CCT AT (pPDI) ATT CTG TCT GG
5	Strand 10	CCA GAC AGA AT (pPDI) TTT CCT ACA GG
6	Strand 6	CCT GTA GGA AA T TTT TTT TTA GGT GCA AG
7	Strand 7	CTT GCA CCT AA

Table S4. Sequences for **pPDI$_5$**.

Position	Strand ID	Sequence 5' → 3'
1	Strand 1	ATA ACT CGG TC
2	Strand 11	GAC CGA GTT AT (pPDI) TAA CCA GTG GA
3	Strand 9	TCC ACT GGT TA (pPDI) ATA GGC GGA AC
4	Strand 8	GTT CCG CCT AT (pPDI) ATT CTG TCT GG
5	Strand 10	CCA GAC AGA AT (pPDI) TTT CCT ACA GG
6	Strand 12	CCT GTA GGA AA (pPDI) TTA GGT GCA AG
7	Strand 7	CTT GCA CCT AA

Table S5. Sequences for **oPDI$_1$**.

Position	Strand ID	Sequence 5' → 3'
1	Strand 1	ATA ACT CGG TC
2	Strand 2	GAC CGA GTT AT T TTT TTT TAA CCA GTG GA
3	Strand 3	TCC ACT GGT TA T TTT TTT ATA GGC GGA AC
4	Strand 13	GTT CCG CCT AT (oPDI) ATT CTG TCT GG
5	Strand 5	CCA GAC AGA AT T TTT TTT TTT CCT ACA GG
6	Strand 6	CCT GTA GGA AA T TTT TTT TTA GGT GCA AG
7	Strand 7	CTT GCA CCT AA

Table S6. Sequences for **oPDI$_2$**.

Position	Strand ID	Sequence 5' → 3'
1	Strand 1	ATA ACT CGG TC
2	Strand 2	GAC CGA GTT AT T TTT TTT TAA CCA GTG GA
3	Strand 14	TCC ACT GGT TA (oPDI) ATA GGC GGA AC
4	Strand 13	GTT CCG CCT AT (oPDI) ATT CTG TCT GG
5	Strand 5	CCA GAC AGA AT T TTT TTT TTT CCT ACA GG
6	Strand 6	CCT GTA GGA AA T TTT TTT TTA GGT GCA AG
7	Strand 7	CTT GCA CCT AA

Table S7. Sequences for **oPDI$_3$**.

Position	Strand ID	Sequence 5' → 3'
1	Strand 1	ATA ACT CGG TC
2	Strand 2	GAC CGA GTT AT T TTT TTT TAA CCA GTG GA
3	Strand 14	TCC ACT GGT TA (oPDI) ATA GGC GGA AC
4	Strand 13	GTT CCG CCT AT (oPDI) ATT CTG TCT GG
5	Strand 15	CCA GAC AGA AT (oPDI) TTT CCT ACA GG
6	Strand 6	CCT GTA GGA AA T TTT TTT TTA GGT GCA AG
7	Strand 7	CTT GCA CCT AA

Table S8. Sequences for **oPDI$_5$**.

Position	Strand ID	Sequence 5' → 3'
1	Strand 1	ATA ACT CGG TC
2	Strand 16	GAC CGA GTT AT (oPDI) TAA CCA GTG GA
3	Strand 14	TCC ACT GGT TA (oPDI) ATA GGC GGA AC
4	Strand 13	GTT CCG CCT AT (oPDI) ATT CTG TCT GG
5	Strand 15	CCA GAC AGA AT (oPDI) TTT CCT ACA GG
6	Strand 17	CCT GTA GGA AA (oPDI) TTA GGT GCA AG
7	Strand 7	CTT GCA CCT AA

Table S9. Sequences for **Por$_1$**.

Position	Strand ID	Sequence 5' → 3'
1	Strand 1	ATA ACT CGG TC
2	Strand 2	GAC CGA GTT AT T TTT TTT TAA CCA GTG GA
3	Strand 3	TCC ACT GGT TA T TTT TTT ATA GGC GGA AC
4	Strand 18	GTT CCG CCT AT (Por) ATT CTG TCT GG
5	Strand 5	CCA GAC AGA AT T TTT TTT TTT CCT ACA GG
6	Strand 6	CCT GTA GGA AA T TTT TTT TTA GGT GCA AG
7	Strand 7	CTT GCA CCT AA

Table S10. Sequences for **Por$_2$**.

Position	Strand ID	Sequence 5' → 3'
1	Strand 1	ATA ACT CGG TC
2	Strand 2	GAC CGA GTT AT T TTT TTT TAA CCA GTG GA
3	Strand 19	TCC ACT GGT TA (Por) ATA GGC GGA AC
4	Strand 18	GTT CCG CCT AT (Por) ATT CTG TCT GG
5	Strand 5	CCA GAC AGA AT T TTT TTT TTT CCT ACA GG
6	Strand 6	CCT GTA GGA AA T TTT TTT TTA GGT GCA AG
7	Strand 7	CTT GCA CCT AA

Table S11. Sequences for **Por$_3$**.

Position	Strand ID	Sequence 5' → 3'
1	Strand 1	ATA ACT CGG TC
2	Strand 2	GAC CGA GTT AT T TTT TTT TAA CCA GTG GA
3	Strand 19	TCC ACT GGT TA (Por) ATA GGC GGA AC
4	Strand 18	GTT CCG CCT AT (Por) ATT CTG TCT GG
5	Strand 20	CCA GAC AGA AT (Por) TTT CCT ACA GG
6	Strand 6	CCT GTA GGA AA T TTT TTT TTA GGT GCA AG
7	Strand 7	CTT GCA CCT AA

Table S12. Sequences for **Por$_5$**.

Position	Strand ID	Sequence 5' → 3'
1	Strand 1	ATA ACT CGG TC
2	Strand 21	GAC CGA GTT AT (Por) TAA CCA GTG GA
3	Strand 19	TCC ACT GGT TA (Por) ATA GGC GGA AC
4	Strand 18	GTT CCG CCT AT (Por) ATT CTG TCT GG
5	Strand 20	CCA GAC AGA AT (Por) TTT CCT ACA GG
6	Strand 22	CCT GTA GGA AA (Por) TTA GGT GCA AG
7	Strand 7	CTT GCA CCT AA

Table S13. Sequences for **pPDI-Por**.

Position	Strand ID	Sequence 5' → 3'
1	Strand 1	ATA ACT CGG TC
2	Strand 2	GAC CGA GTT AT T TTT TTT TAA CCA GTG GA
3	Strand 9	TCC ACT GGT TA (pPDI) ATA GGC GGA AC
4	Strand 18	GTT CCG CCT AT (Por) ATT CTG TCT GG
5	Strand 5	CCA GAC AGA AT T TTT TTT TTT CCT ACA GG
6	Strand 6	CCT GTA GGA AA T TTT TTT TTA GGT GCA AG
7	Strand 7	CTT GCA CCT AA

Table S14. Sequences for **oPDI-pPDI**.

Position	Strand ID	Sequence 5' → 3'
1	Strand 1	ATA ACT CGG TC
2	Strand 2	GAC CGA GTT AT T TTT TTT TAA CCA GTG GA
3	Strand 9	TCC ACT GGT TA (pPDI) ATA GGC GGA AC
4	Strand 13	GTT CCG CCT AT (oPDI) ATT CTG TCT GG
5	Strand 5	CCA GAC AGA AT T TTT TTT TTT CCT ACA GG
6	Strand 6	CCT GTA GGA AA T TTT TTT TTA GGT GCA AG
7	Strand 7	CTT GCA CCT AA

Table S15. Sequences for **oPDI-(pPDI)$_1$-Por**.

Position	Strand ID	Sequence 5' → 3'
1	Strand 1	ATA ACT CGG TC
2	Strand 2	GAC CGA GTT AT T TTT TTT TAA CCA GTG GA
3	Strand 14	TCC ACT GGT TA (oPDI) ATA GGC GGA AC
4	Strand 8	GTT CCG CCT AT (pPDI) ATT CTG TCT GG
5	Strand 20	CCA GAC AGA AT (Por) TTT CCT ACA GG
6	Strand 6	CCT GTA GGA AA T TTT TTT TTA GGT GCA AG
7	Strand 7	CTT GCA CCT AA

Table S16. Sequences for **oPDI-(pPDI)$_2$-Por**.

Position	Strand ID	Sequence 5' → 3'
1	Strand 1	ATA ACT CGG TC
2	Strand 16	GAC CGA GTT AT (oPDI) TAA CCA GTG GA
3	Strand 9	TCC ACT GGT TA (pPDI) ATA GGC GGA AC
4	Strand 8	GTT CCG CCT AT (pPDI) ATT CTG TCT GG
5	Strand 20	CCA GAC AGA AT (Por) TTT CCT ACA GG
6	Strand 6	CCT GTA GGA AA T TTT TTT TTA GGT GCA AG
7	Strand 7	CTT GCA CCT AA

Table S17. Sequences for **oPDI-(pPDI)$_3$-Por**.

Position	Strand ID	Sequence 5' → 3'
1	Strand 1	ATA ACT CGG TC
2	Strand 16	GAC CGA GTT AT (oPDI) TAA CCA GTG GA
3	Strand 9	TCC ACT GGT TA (pPDI) ATA GGC GGA AC
4	Strand 8	GTT CCG CCT AT (pPDI) ATT CTG TCT GG
5	Strand 10	CCA GAC AGA AT (pPDI) TTT CCT ACA GG
6	Strand 22	CCT GTA GGA AA (Por) TTA GGT GCA AG
7	Strand 7	CTT GCA CCT AA

The oligonucleotides were synthesized trityl-on on solid supports (Glen Research, 1000 Å CPG) at a 1.0 µmol scale using standard protocols. The synthesis was carried out on an ABI 394 DNA/RNA Synthesizer in the standard mode, using 2-cyanoethyl-*N*,*N*-diisopropylphosphoramidites under inert conditions with anhydrous solvents. Detritylation with deblock (3% Trichloroacetic acid in DCM) was followed by a MeCN wash. Phosphoramidite and activator (0.25 M ETT in MeCN) were loaded onto the column and coupled for 40 s. The column was washed with MeCN. The resin was oxidised (0.02 M Iodine in THF/Water/Pyridine). The column was washed with MeCN. Failure sequences were capped with Cap Mix A (THF/Acetic anhydride) and Cap Mix B (10 % 1-methylimidazole in THF). The column was washed with MeCN, dried, and the cycle repeated for the next nucleobase phosphoramidite.

For installing the semiconductor component, the 11-mer resin column was transferred to an Expedite 8900 Nucleic Acid Synthesis System. First the column was detritylated with deblock (3% Trichloroacetic acid in DCM), followed by a MeCN wash. For the monomer coupling step activator (0.25 M ETT in MeCN), semiconductor phosphoramidite (6, 10, and 16 in DCM), and DCM were injected sequentially into the column in 4:10:1 volume ratios, and coupled for 7 minutes with pulses of activator every 1 min to ensure a steady flow of fresh mixture; this cycle was repeated three times. The column was washed with DCM until no colour was observed in the washings. The colour of the resin was inspected by eye to estimate semiconductor phosphoramidite coupling efficiency, if yields appeared poor the monomer coupling cycle was repeated. Sequential oxidation, capping, steps were identical to those previously described on the ABI 394 DNA/RNA Synthesizer.

The semiconductor-modified strands were transferred back to the ABI 392 DNA/RNA Synthesizer, and an additional 11 DNA bases were coupled as previously described.

The completed oligonucleotide solid phases were incubated in diethylamine for 30 mins, washed with MeCN, and incubated overnight with 1 mL concentrated ammonium hydroxide. The solutions were diluted by two with NaCl (100 mg/mL). The mixtures were loaded onto a Glen-Pack™ DNA purification cartridge 60-5200-10 (pre-washed with MeCN and TEAA), washed with brine, detritylated with 2% TFA, washed with H_2O, and eluted in MeCN:H_2O (1:1). The resulting red solutions were lyophilized.

2. Supplementary Library Spectra

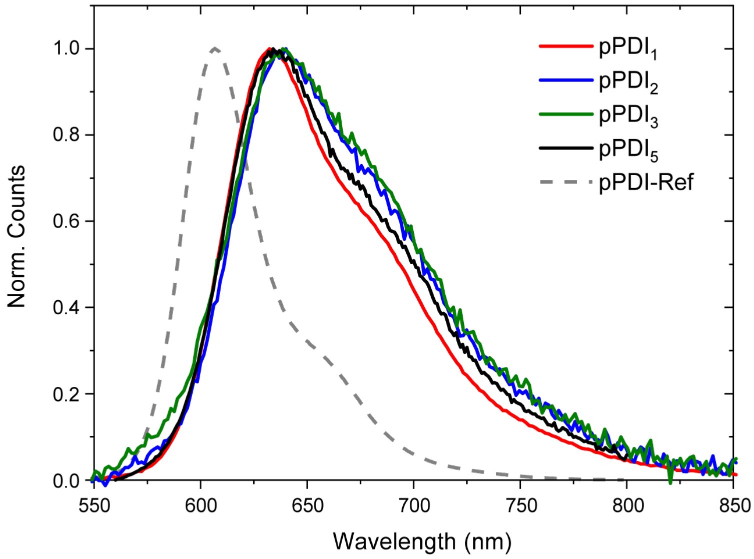

Figure S1. pPDI emission. Steady-state emission of pPDI constructs. λ_{ex} = 525 nm. Normalised to emission maxima. pPDI-Ref = **9**.

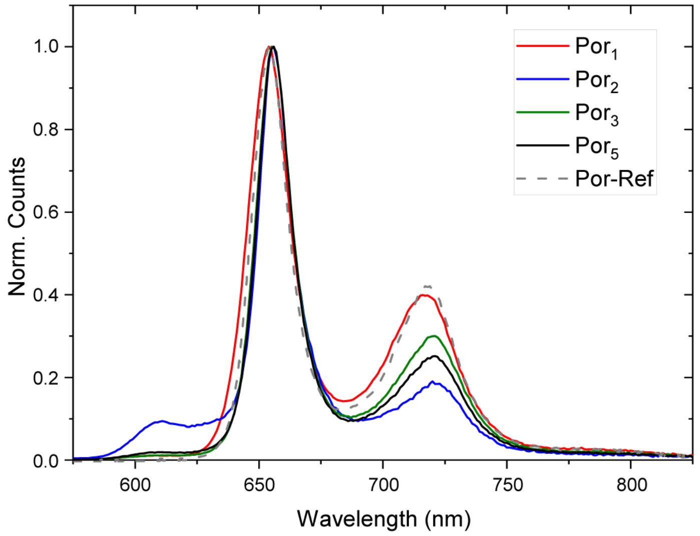

Figure S2. Por-DNA conjugate emission. λ_{ex} = 405 nm. Normalised to emission maxima. 60–70 µM. Por-Ref = **3**.

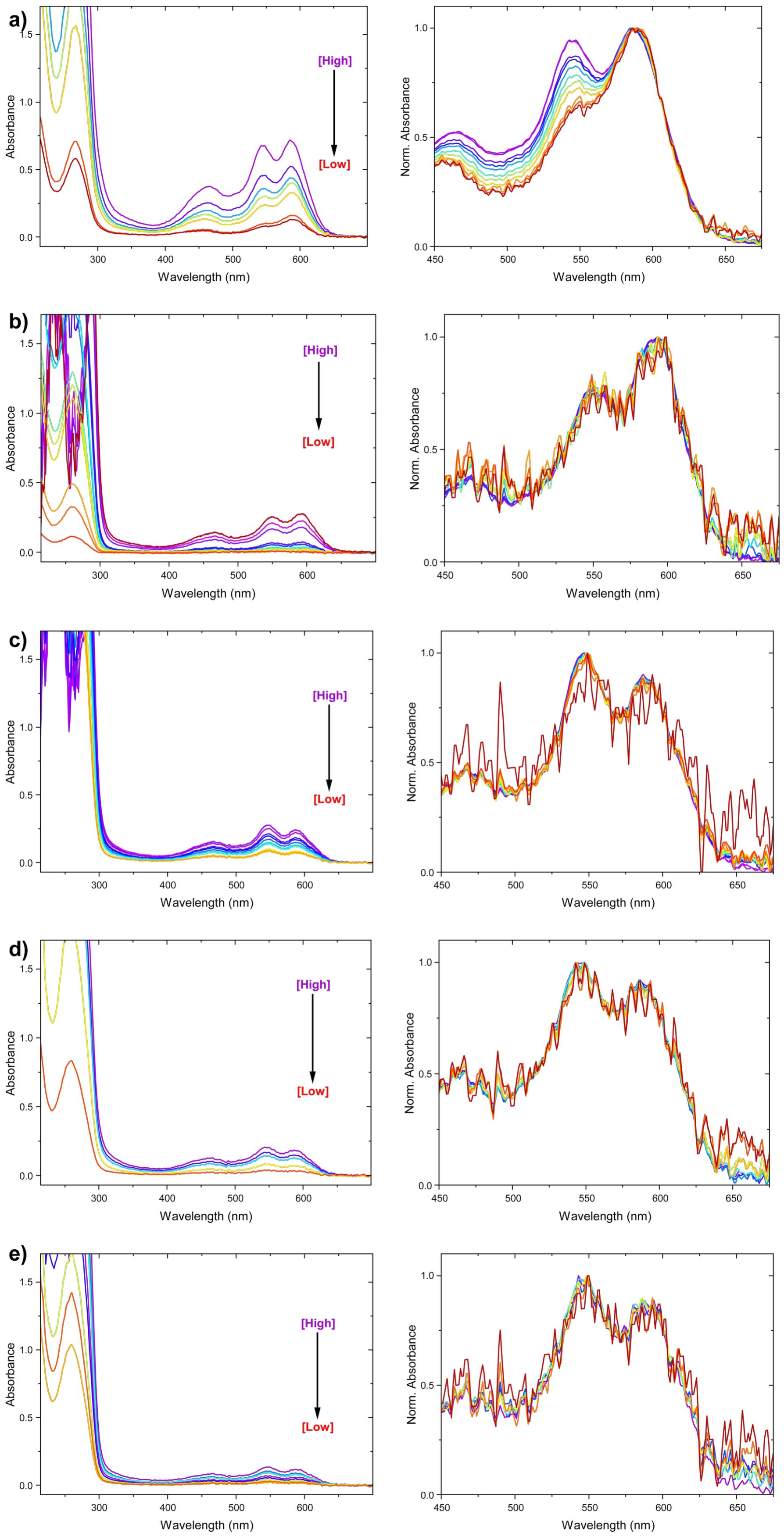

Figure S3. Concentration dependent UV-vis. (left) raw data, (right) normalised to the pPDI absorption maxima. PBS. 0.1–75 µM. **a)** pPDI-ssDNA with no Placeholder strands. **b)** $pPDI_1$. **c)** $pPDI_2$. **d)** $pPDI_3$. **e)** $pPDI_5$.

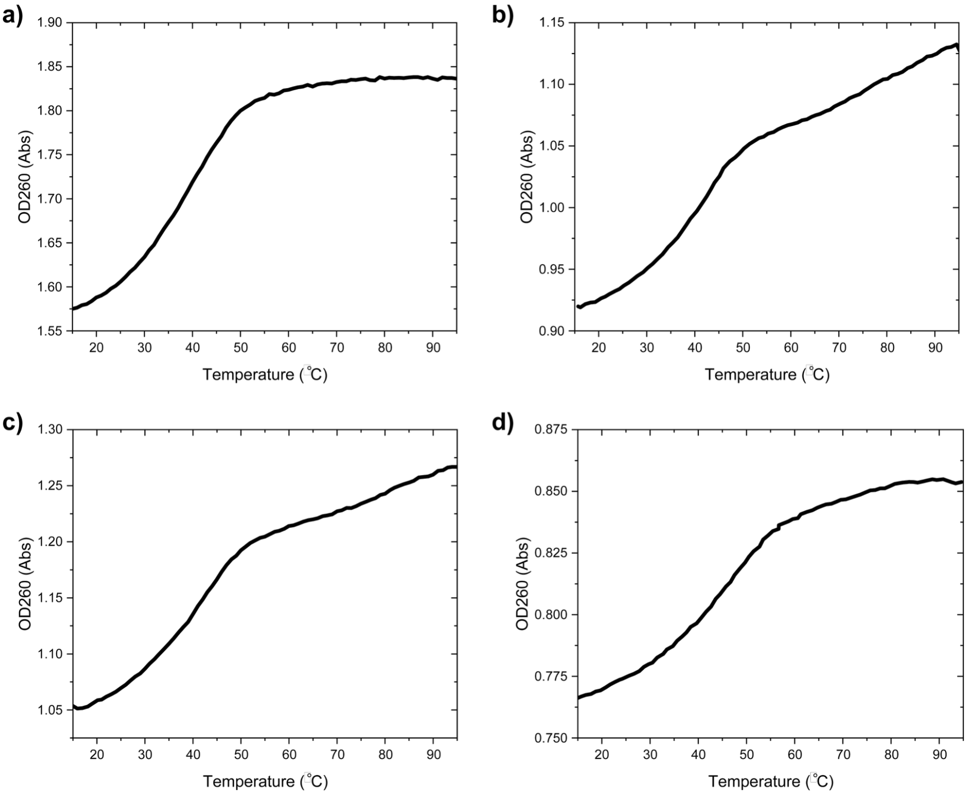

Figure S4. DNA 260 nm melting curves, 1 X PBS, 40 µg/mL. **a)** $pPDI_1$, T_m = 39.9 °C. **b)** $pPDI_2$, T_m = 40.5 °C. **c)** $pPDI_3$, T_m = 43.7 °C. **d)** $pPDI_5$, T_m = 45.6 °C.

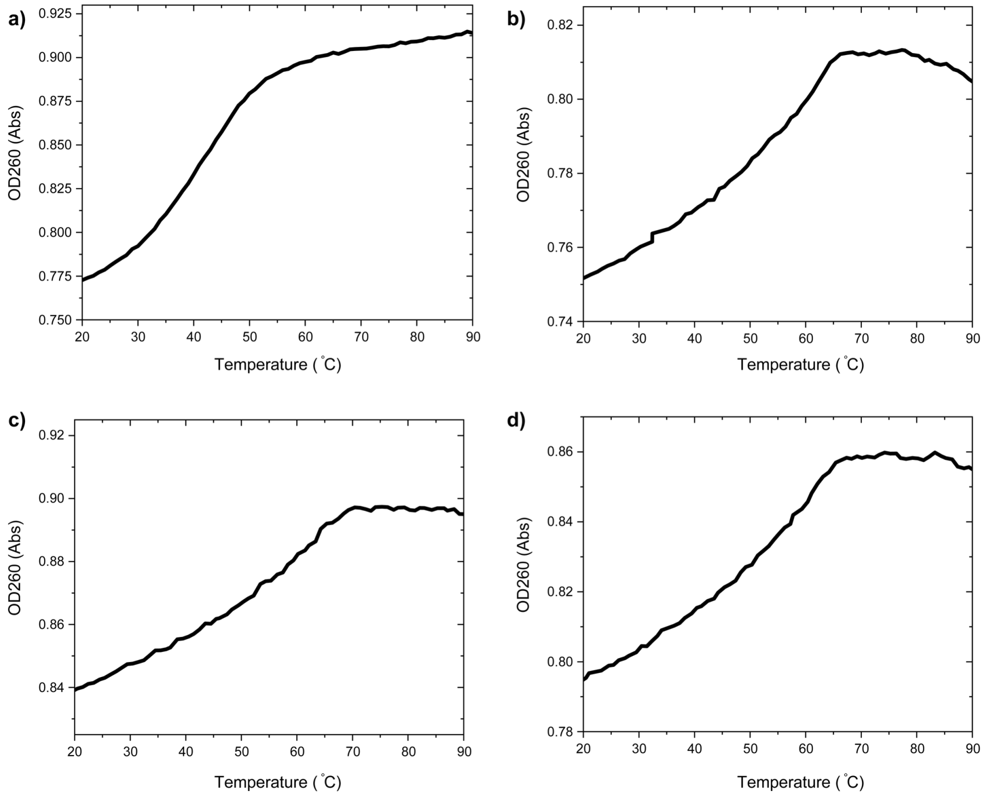

Figure S5. DNA 260 nm melting curves, 1 X PBS, 40 µg/mL. **a)** Por_1, T_m = 41.1 °C. **b)** Por_2, T_m = 61.6 °C. **c)** **Por**$_3$, T_m = 59.1 °C. **d)** Por_5, T_m = 57.3 °C.

4. Atomistic Metadynamics Molecular Dynamics Simulations

Parameterisation

For pPDI, the semiconductors and linkers were parameterised independently (Figure S6). The structures of the semiconductors were first drawn using MarvinSketch and exported as mol2 files. Methyl capping groups were used for the semiconductor. In the case of the linkers, acetyl and PO_3CH_3 capping groups were used. Hydrogen atoms were added to sketched molecules using Chimera.[10] The PyRED server[11] was used to calculate the electrostatic potential of the structures at the HF/6-31G* level of theory using Gaussian09, and then perform a two-stage Restrained Electrostatic Potential (RESP)[12] fit to calculate the atomic charges. During the RESP fit, the partial atomic charges of the capping groups (Ac/Me/PO_3CH_3) were restrained to match their values in the ff99sb[13,14] and parmbsc1[15] parameter set. Antechamber[16] was then used to assign atom-types to the semiconductor/linkers according to the GAFF2 parameter[17] set. For Porphyrin with no alkyl linker, a similar procedure was employed but with two PO_3CH_3 capping groups at each end.

The dsDNA strands were built as 6 base pairs long B-DNA using Avogadro.[18] The xleap module of Amber16[19] was then used to remove the capping groups and append the semiconductor, linker and DNA molecules. The DNA strands were parameterized using the parmbsc1 parameters.[15] The system was solvated in an octahedral box of TIP3P[20] solvent and 0.15 M NaCl ions that used the parameters of Joung and Cheatham.[21] Finally, the amber parameters were converted to Gromacs[22] format using the Parmed.[23]

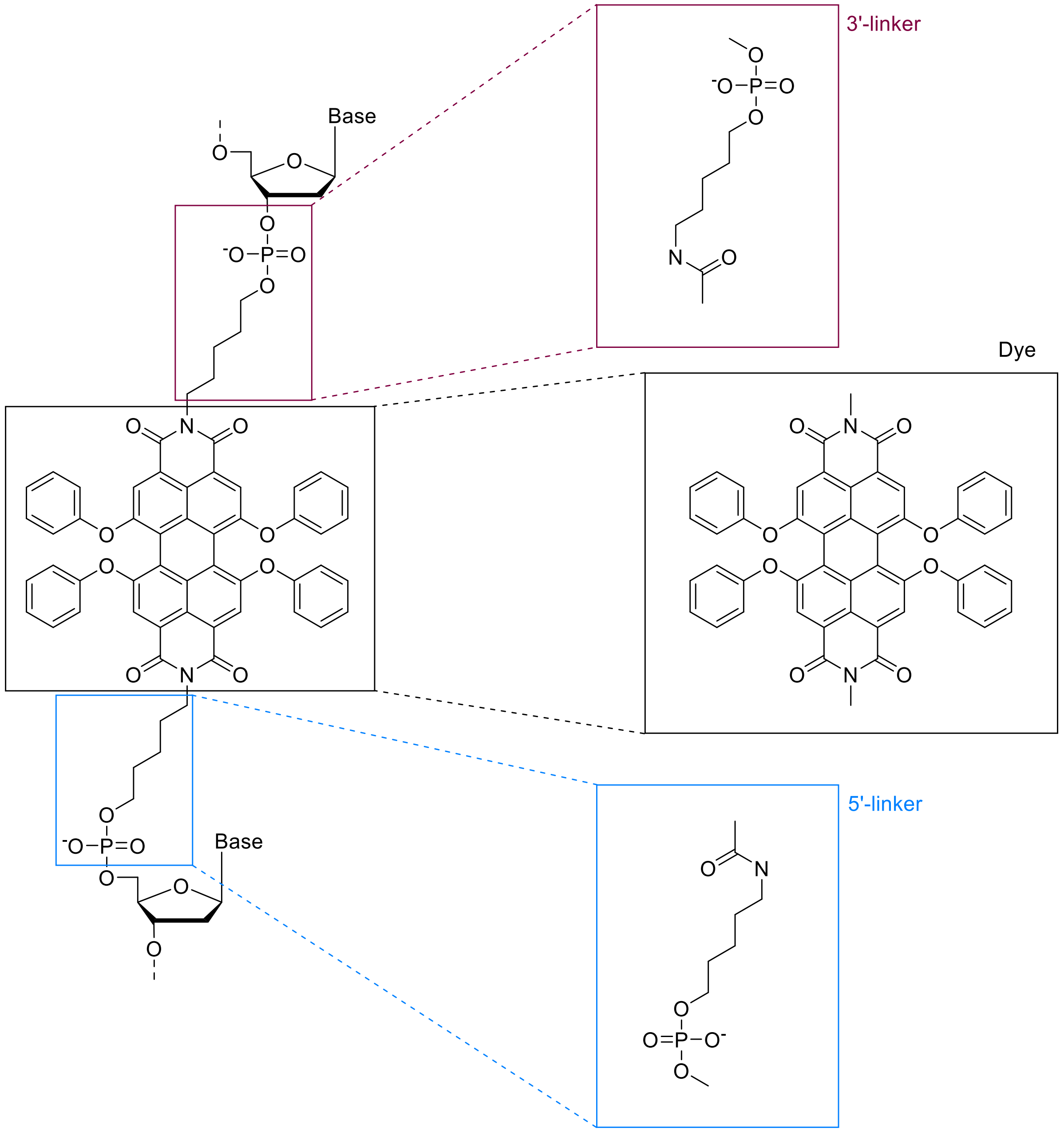

Figure S6. Parametrisation of **oPDI** for MD simulation.

Metadynamics Simulation Setup

Models were first energy minimized until the maximum force on any of the atoms was below 1000 kJ mol^{-1} nm^{-1}. The systems were then equilibrated using restraints on the DNA and semiconductors (force constants of 1000 kJ mol^{-1} nm^{-2}) for 1 ns in the NVT ensemble followed by 1 ns in the NPT ensemble.

The simulations were performed starting with random velocities obtained from a Maxwell–Boltzmann distribution at 300 K and using a pressure of 1 bar. The temperature was kept constant using the V-rescale thermostat.[24] The pressure was maintained using the Parrinello-Rahman barostat.[25] Long-range electrostatics were calculated using the particle mesh Ewald (PME) algorithm[26] with a cutoff of 1.0 nm. The simulations used a timestep of 2 fs and were performed using Gromacs 2019[22] patched with Plumed 2.6.1.[27,28] Trajectories were analysed using a combination of Gromacs and Plumed tools together with Python MDAnalysis scripts.[29]

Metadynamics Simulations

Following equilibration, we perform well-tempered metadynamics simulations,[30,31] which uses an external biasing potential to promote efficient sampling along chosen Collective Variables (ξ). The metadynamics biasing potential V_E used is constructed as a sum of gaussians deposited along the Collective Variable space (ξ_i) as:

$$V_E = \sum_{\{t=\tau_G,2\tau_G\ldots\}} W \prod_i e^{\left(-\frac{(\xi_i-\xi_{ti})}{2\delta_i^2}\right)^2} \quad (1)$$

where τ_G is the time interval at which the gaussians are added with height W, width δ and mean ξ_{ti}. We used a two-dimensional gaussian (i = 2) to simultaneously bias the system along two important collective variables that determine the relative stacking orientations of the two semiconductors (Figure S7). ξ_1 is defined as the Euclidean distance between the centres of the central aromatic rings of the two semiconductors. ξ_2 is defined as the angle between two vectors identified by two pairs of atoms and is calculated as:

$$\theta = \ arccos\left(\frac{\vec{r}_{21}\cdot\vec{r}_{34}}{|\vec{r}_{21}||\vec{r}_{34}|}\right) \quad (2)$$

where 1-2 and 3-4 are the terminal Nitrogen atoms of the two semiconductor molecules. However, it should be noted that, by definition, this ξ_2 includes the offset angle both along and orthogonal to the semiconductor molecule plane.

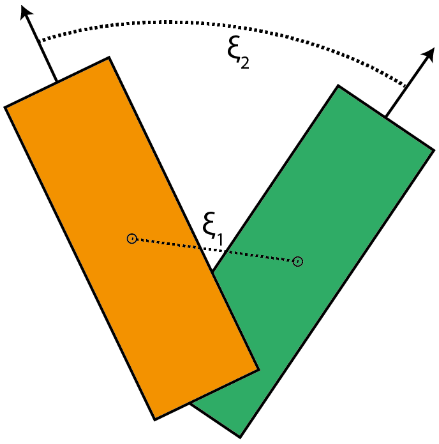

Figure S7. Illustration of the distance and angle metadynamics collective variables.

The metadynamics biasing potentials were deposited every 10 ps using a bias factor of 15 and gaussian widths of 0.008 nm and 0.025 radians along ξ_1 and ξ_2 respectively. The simulations were performed for 100 ns and convergence was assessed using the time-evolution of the free-energy profiles.

The addition of the history dependant bias however precludes a direct ensemble averaging of the system's characteristics. Thus, the methodology of Tiwary and Parrinello[32] was used to reweight the trajectory frames and subsequently calculate the equilibrium distribution of semiconductor stacking configurations (orthogonal ($R_\perp$) and planar ($R_\parallel$) distances, Figure 2 in the main manuscript). Briefly, the Probability Distribution of the biased simulated system ($P(R,t)$) as a function of its atomic coordinates 'R' can be expressed as:

$$P(R) = \frac{exp(-\beta[U(R) + V_E(\xi(R))])}{\int exp(-\beta[U(R) + V_E(\xi(R))])\, dR} \quad (3)$$

where $U(R)$ is the internal potential and $V(\xi(R),t)$ is the metadynamics bias potential. The introduction of the delta function $\delta(\xi - \xi(R))$ and the unbiased probability density function $P_0(R,t)$ allows the expression of the equation as:

$$P(R) = P_0(R) \cdot exp(-\beta[V_E(\xi(R)) - c(t)]) \quad (4)$$

where $c(t)$ is the time-dependant bias offset defined as:

$$c(t) = \frac{1}{\beta} ln\left[\frac{\int exp(-\beta F(\xi))\, d\xi}{\int exp(-\beta[F(\xi) + V_E(\xi)])\, d\xi}\right] \tag{5}$$

The $c(t)$ offset is calculated using Plumed following the protocol of Tiwary and Parrinello[32] and used to assign weights

$$w(t) \propto exp(\beta[V(\xi(R), t) - c(t)])$$

from the biased probability density. See Bonomi *et al.*[33] for a complete derivation.

The equilibrium distribution of the orthogonal ($R_\perp$) and planar ($R_\parallel$) distances between the semiconductors were then calculated as a weighted histogram using these weights $w(t)$.

$R_\perp$ and $R_\parallel$ Calculation

For each trajectory frame, the cartesian equation of a plane describing the first semiconductor:

$$ax + by + cz + d = 0 \tag{6}$$

was calculated using a least-square regression fit of its central aromatic ring using the Python scipy library[34] (Figure S8).

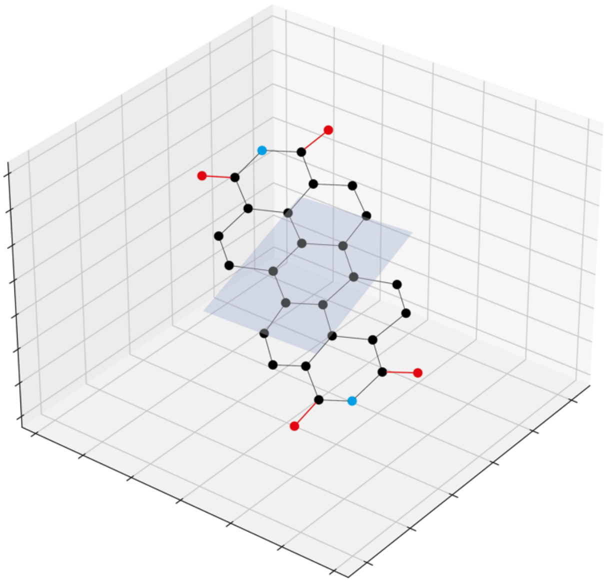

Figure S8. Illustration of the semiconductor and the plane fitted to its central aromatic ring.

The orthogonal distance of the second semiconductor to this fitted plane ($R_\perp$) for the trajectory frame was then calculated as:

$$R^\perp = \frac{|a \cdot x_0 + b \cdot y_0 + c \cdot z_0 + d|}{\sqrt{a^2 + b^2 + c^2}} \tag{7}$$

where (x_0, y_0, z_0} is the centre of the central aromatic ring of the second semiconductor. The planar distance $R_\parallel$ for the frame was then calculated as

$$R^\parallel = \sqrt{(R^{euc.})^2 - (R^\perp)^2} \tag{8}$$

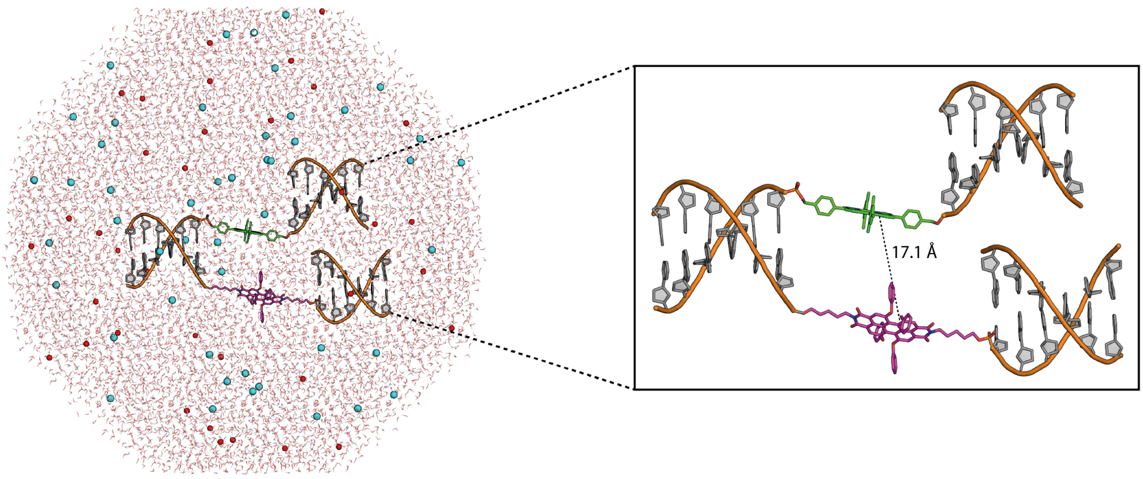

Figure S9. Initial geometry used in the MD simulations (here pPDI-Por). The semiconductors are well separated by water and ions to avoid any bias towards π-stacking.

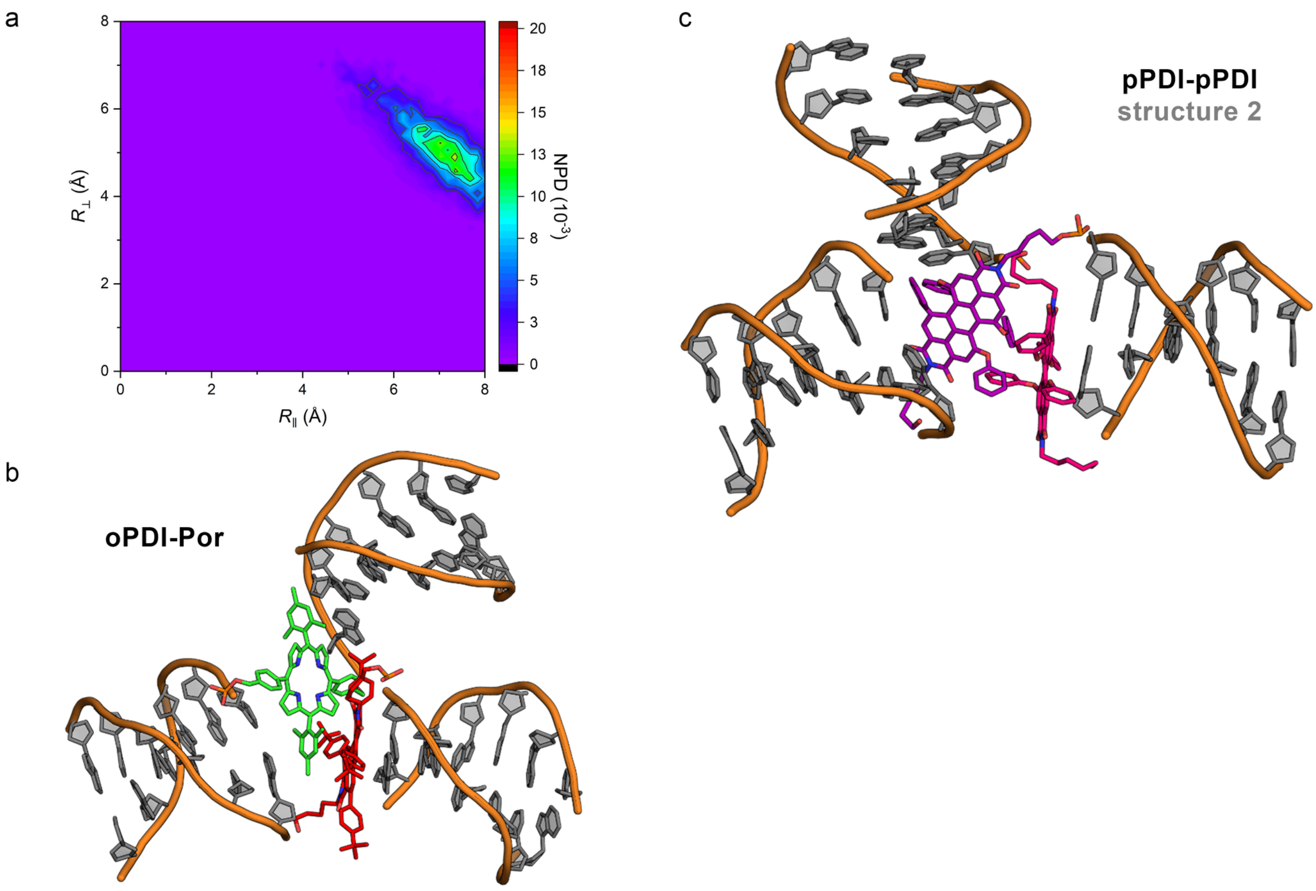

Figure S10. Supplementary simulated semiconductor-component dimer structures. (**a**) Structure normalised probability densities (NPDs) of oPDI-Por as a function of orthogonal ($R_\perp$) and planar ($R_\parallel$) centre-to-centre distances between planes of the semiconductors. Sum of all grid element probabilities is normalised to 1. (**b**) Snapshot of the most-probable structure of oPDI-Por. Solvent and ions are omitted for clarity. (c) Snapshot of the most-probable structure corresponding to the second minimum of pPDI-pPDI (see main manuscript, Figure 2). Solvent and ions are omitted for clarity.

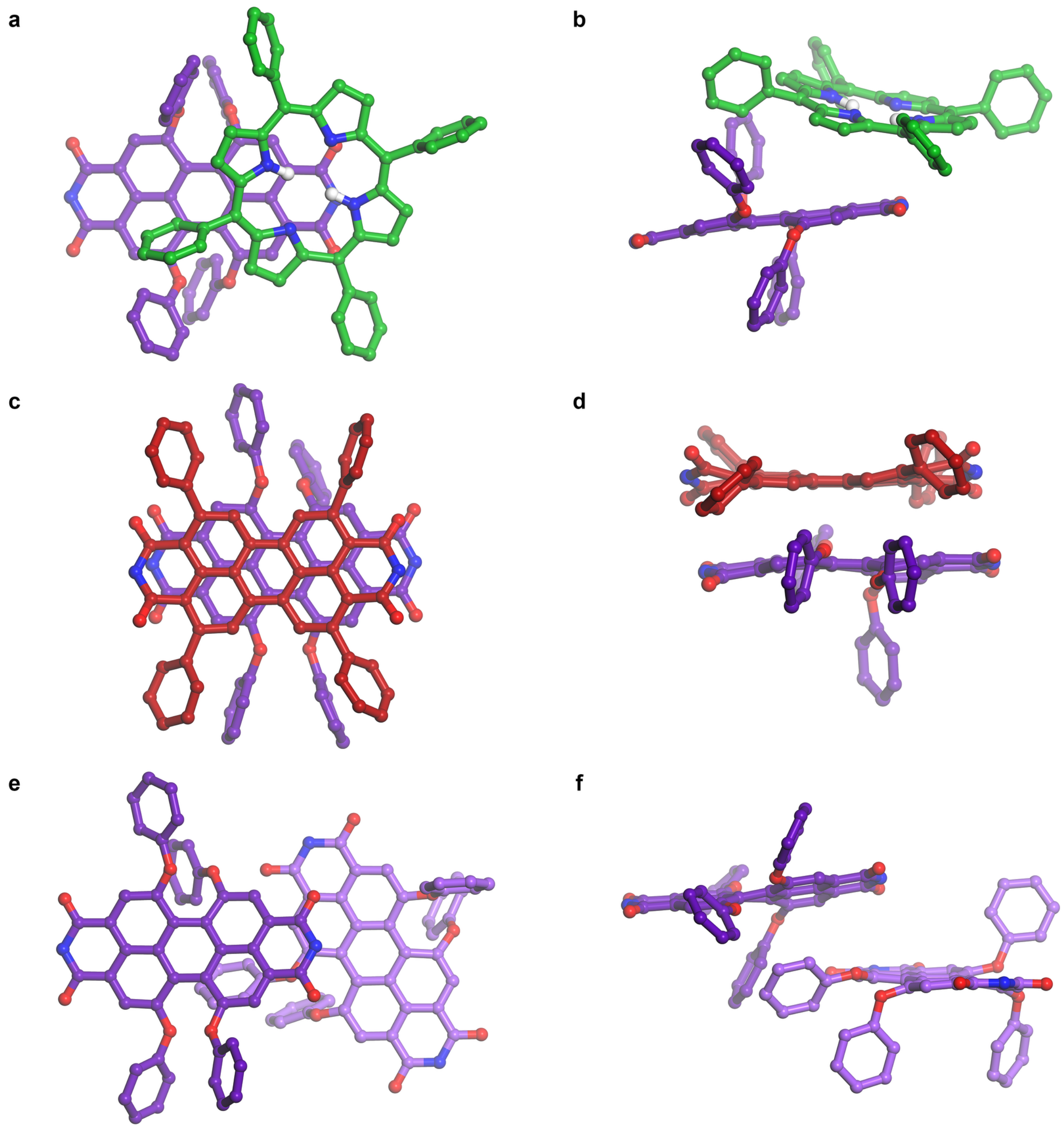

Figure S11. Top-down (left) and side-on (right) snapshots of highest NPD structures of al MD dimers. (**a**,**b**) pPDI-Por, (**c**,**d**) oPDI-pPDI, (**e**,**f**) pPDI-pPDI. DNA omitted for clarity.

5. Modelling of Larger Aggregates

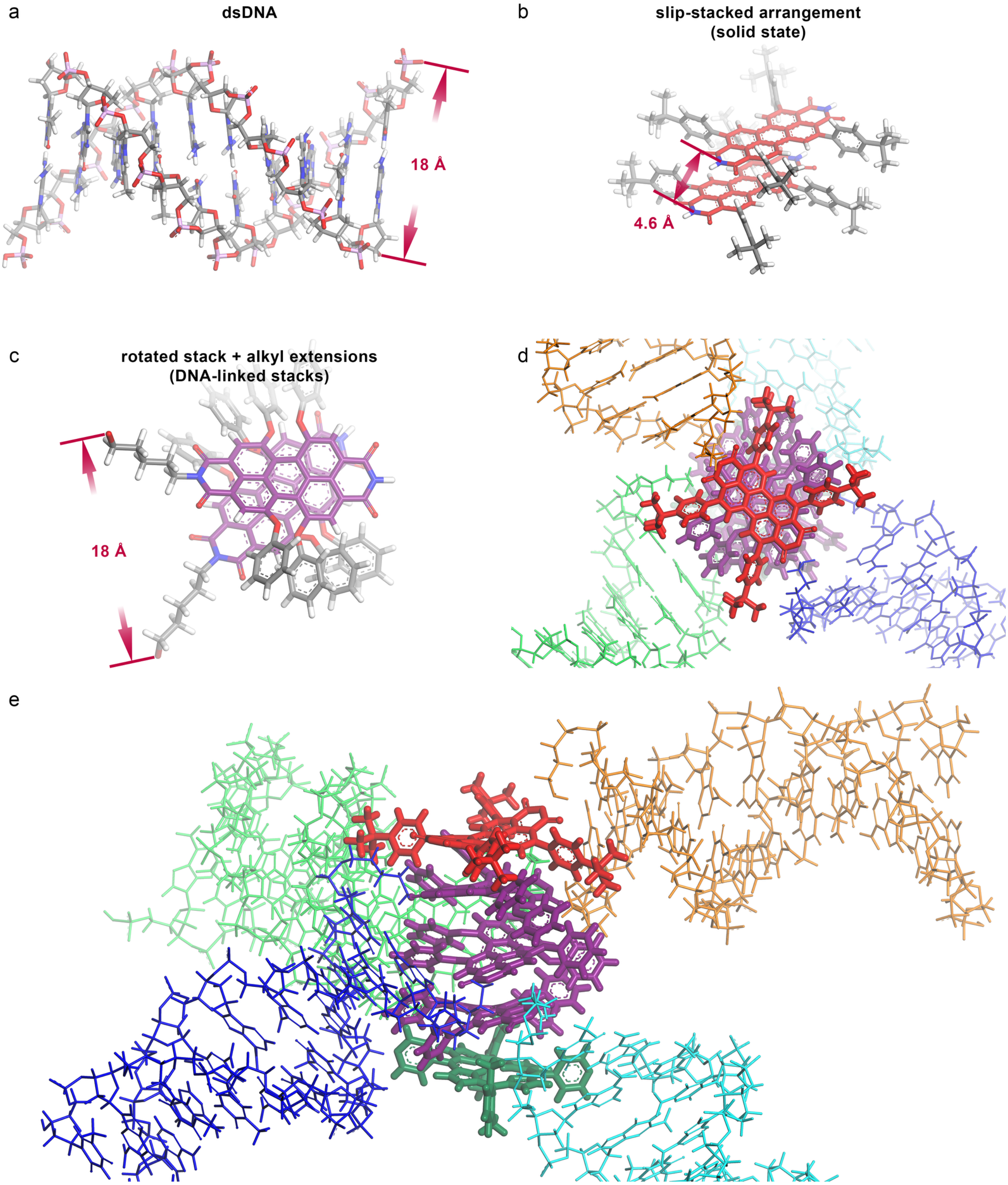

Figure S12. Simulation of an **oPDI-(pPDI)$_3$-Por** pentamer. Colour code: **oPDI** – red; **pPDI** – purple; **Por** – green. The dsDNA are colour coded according to Figure 1 in the main manuscript. The terminating dsDNA on either end of the stack have been omitted for clarity. (**a**) The distance between the two semiconductor anchoring points on the phosphate backbone of dsDNA is about 18 Å. (**b**) Conversely, the attachment points in close-packed semiconductors such as PDIs are only 4 - 7 Å apart. (**c**) In order to compensate for this size mismatch, we equipped the semiconductors with short extensions (pentyl chains shown here with the pPDI dimer).

Additionally, the semiconductors are designed such that they can adopt a rotated arrangement, which places the DNA connection points on the ends of the alkyl chains 18 Å apart – exactly the distance needed for the connection to dsDNA. (**d**, **e**) The resulting architectures consist of a central column of approximately coplanar semiconductors surrounded by the connecting dsDNA strands. Due to the rotational offset between the individual dsDNA, there is no sterics limit to the size of the stacks.

Due to the large number of atoms (the pentamers consist of <4400 atoms), modelling of the complete semiconductor assemblies was performed using force-field methods. Starting geometries were built using the Accelrys Materials Studio software package and were based on the MD-optimised dimer structures. Geometry optimisations were performed using the Dreiding force field. The architectures are designed such that the coarse positioning of the semiconductors within the stacks is achieved via the specific recognition of a complementary base sequence in the DNA duplexes, providing deterministic control over the semiconductor sequence. On a sub-nm length scale, the semiconductors have enough flexibility to aggregate in a favourable geometry.

Organic semiconductors such as the PDIs and porphyrins used in our study tend to stack at 3.5 - 5 Å π-π distance with 4 - 7 Å centre-to-centre distance including lateral offset (Figure S11), depending on their side groups. However, the distance between the phosphate backbones of the two complementary strands in a DNA helix (where the semiconductors are attached) is ~18 Å (Figure S10). In order to compensate for this size mismatch, we use a combination of two design strategies:

We installed short extensions – pentyl chains on the PDI imides, benzyl groups on the porphyrin – which provide a slightly flexible connection between the DNA and the semiconductor cores, but are sufficiently short not to compromise the exact definition of the semiconductor sequence. An additional rotation of about 45° for PDIs and 90° for porphyrins between adjacent semiconductors brings the DNA attachment points to the required 18 Å distance (Figure S12c). These steric requirements produce well-defined packing motifs along the columnar semiconductor constructs, which can differ from the typical packing motifs of the semiconductors in the solid state.

The semiconductor rotation about the stacking axis also allows considerably more space for the bulky dsDNA helices (Figure S12d,e), enabling extended stacks of theoretically unlimited size.

6. Monomer Steady-State Spectra Compared to $CHCl_3$ Analogues

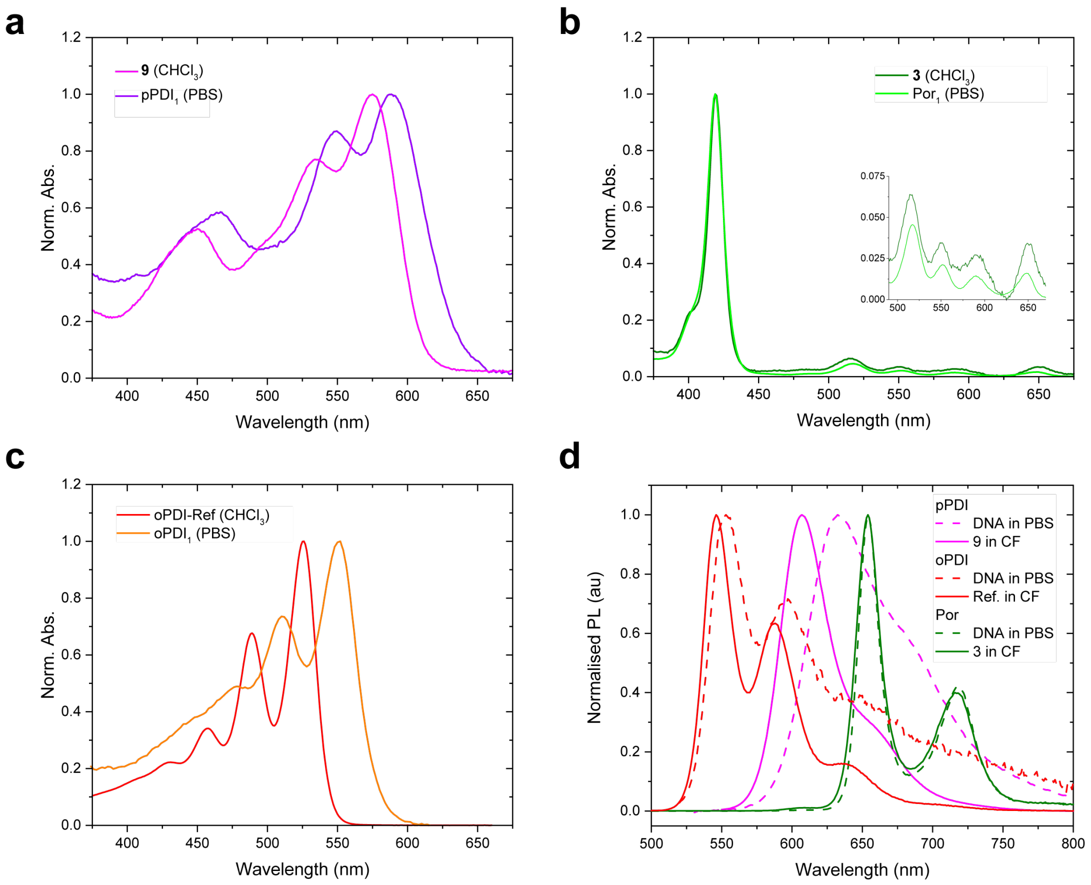

Figure S13. Steady-state optical absorption and emission spectra. See the synthesis section for the chemical formulas of compounds **3** and **9**. (**a**) Intensity normalised absorption spectrum of monomeric **pPDI₁**, and reference dihydroxyl (**9**) in $CHCl_3$ (10 µM). (**b**) Intensity normalised absorption spectrum of monomeric **oPDI₁**, and reference dihydroxyl (oPDI-Ref) in $CHCl_3$ (13 µM).† (**c**) Intensity normalised absorption spectrum of monomeric **Por₁**, and reference dihydroxyl (**3**) in $CHCl_3$ (8 µM). (Inset) Zoom of Q-bands. (**d**) Steady State Emission of all monomeric semiconductors on DNA/PBS (solid line) and reference dihydroxyl in $CHCl_3$ (Dashed). †Data reproduced from Ref.[9]

Table S18. Photoluminescence quantum yields (ϕ_{PL}) of the semiconductors (recorded in triplicate).

Semiconductor	ϕ_{PL} Dihydroxyl Reference ($CHCl_3$) / %	ϕ_{PL} DNA (PBS) / %
$pPDI_1$	85.73 ± 0.06	23.53 ± 1.72
$pPDI_2$	--	0.38 ± 0.03
$pPDI_3$	--	0.83 ± 0.03
$pPDI_5$	--	1.16 ± 0.05
$oPDI_1$†	2.53 ± 0.06	0.55 ± 0.03
$oPDI_2$†	--	1.77 ± 0.01
$oPDI_3$†	--	2.13 ± 0.05
$oPDI_5$†	--	2.53 ± 0.06
Por_1	5.69 ± 0.09	5.55 ± 0.03
Por_2	--	0.82 ± 0.00
Por_3	--	2.47 ± 0.05
Por_5	--	5.69 ± 0.09

†Data from Ref.[9]

7. ns-Transient Absorption Spectroscopy

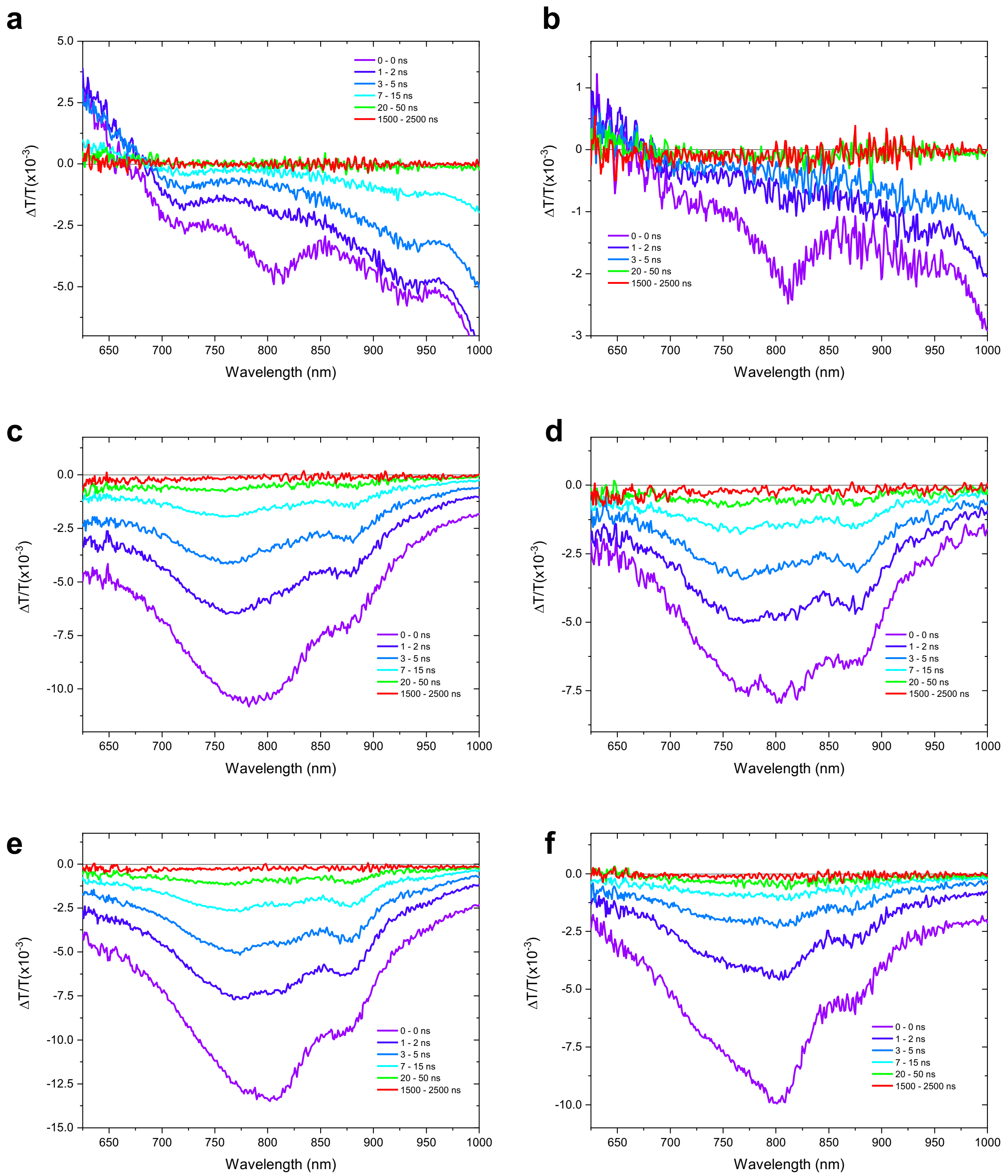

Figure S14. ns-TA. (**a**) pPDI, (**b**) pPDI-Por, (**c**) oPDI-pPDI, (**d**) oPDI-(pPDI)$_1$-Por, (**e**) oPDI-(pPDI)$_2$-Por, (**f**) oPDI-(pPDI)$_3$-Por. λ_{ex} = 585 nm, 64 μJ cm^{-2}.

8. fs-TA of Compound 15 and pPDI Radical Anion Spectra

See the synthesis section for the chemical formulas of compound **9**.

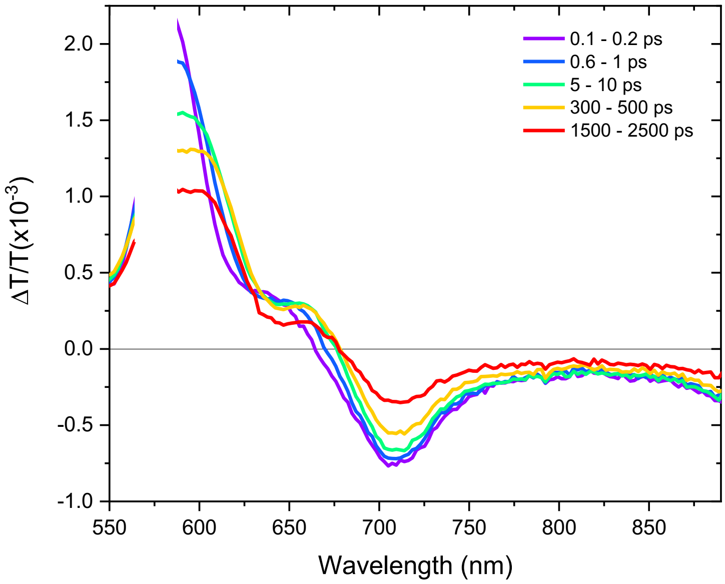

Figure S15. fs-TA of **9** 30 μM in $CHCl_3$, λ_{ex} = 575 nm, 29 μJ cm^{-2}. 570 – 585 nm removed due to pump scatter.

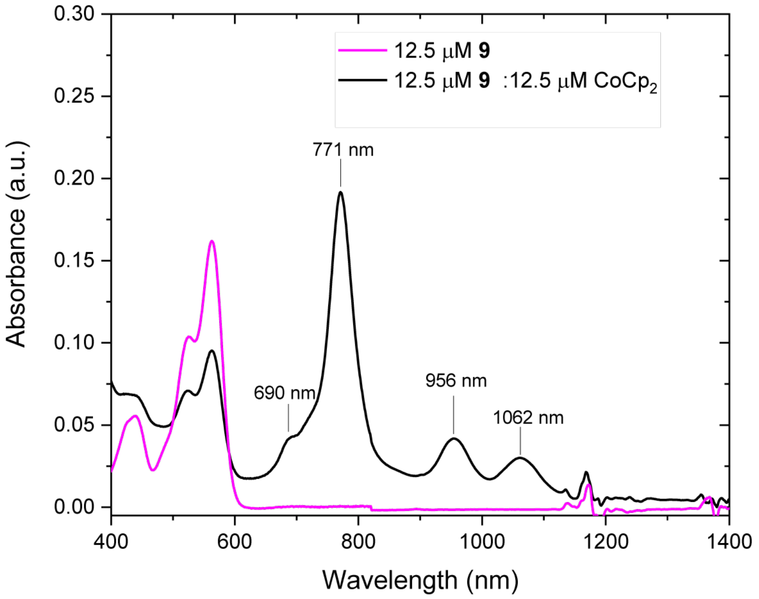

Figure S16. pPDI radical anion by chemical doping. (pink) Spectrum of 12.5 μM pPDI **9** in its neutral state. (black) Radical anion generated from 1:1 (12.5 μM: 12.5 μM) **9**:$CoCp_2$. Solvent: degassed, dry, BHT-free THF.

9. Rehm-Weller Analysis

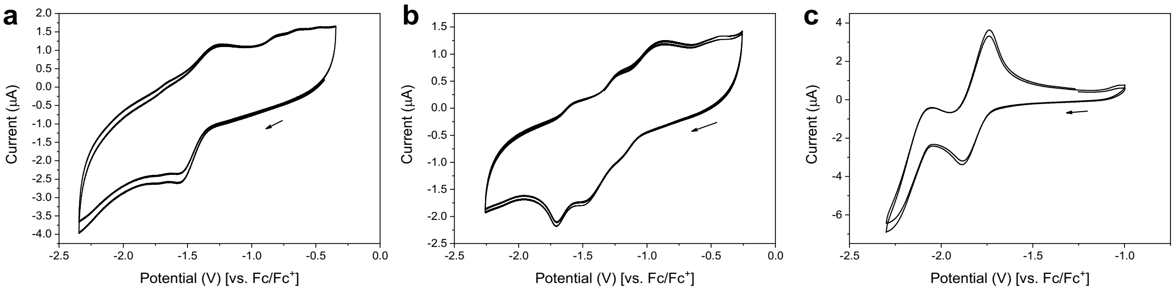

Figure S17. Reduction curves. 200 mV s^{-1} in a 0.1 M $TBAPF_6$/DCM electrolyte under argon. (**a**) pPDI **9**, (**b**) oPDI-ref,† (**c**) Por **3**. The arrow indicates the direction of the scan. See the synthesis section for the chemical formulas of compounds **9**, oPDI-ref, and **3**. †Data reproduced from Ref.[9]

The oxidation curves of dihydroxls were irreversible owing to the free hydroxyls, hence only reduction potentials were taken from the cyclic voltammograms. Lowest unoccupied molecular orbital (LUMO) energy levels were estimated using:

$$LUMO = -(4.8 - E_{\frac{1}{2}}, Fc, Fc^{+} + E_{onset}^{red}) \tag{9}$$

Highest occupied molecular orbital (HOMO) levels were approximated by:

$$HOMO = LUMO - E_g^{opt} \tag{10}$$

Table S19. Estimated Frontier Molecular Orbitals.

	E_{Onset}^{Red} (eV)	LUMO (eV)	E_g^{opt} (eV)[a]	HOMO (eV)
pPDI **9**	-1.22	-3.58	1.98	-5.56
oPDI-ref†	-1.17	-3.63	2.25	-5.88
Por **3**	-1.83	-2.98	1.87	-4.85

[a] Determined from absorption edge in 10 μM $CHCl_3$. For Por, lowest energy Q_x band was used. †Data from Ref.[9]

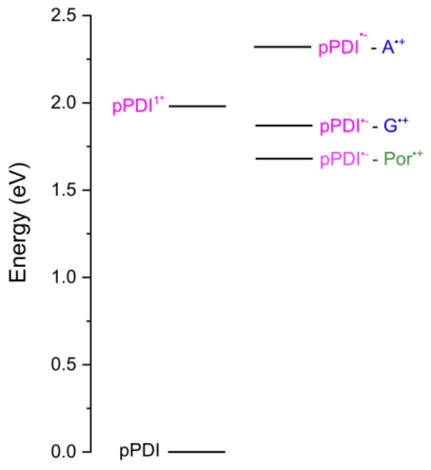

Figure S18. Energetic Scheme comparing **pPDI** levels calculated using Weller formalism.

Weller analysis is based on the Born dielectric-continuum solvent model, and calculates the free energy of an ion-pair ΔG_{IP} in a solvent of arbitrary polarity:

$$\Delta G_{IP} = E_{ox} - E_{red} - \frac{e^2}{r_{DA}\varepsilon_s} + e^2\left(\frac{1}{2r_1} + \frac{1}{2r_2}\right)\left(\frac{1}{\varepsilon_s} + \frac{1}{\varepsilon_{sp}}\right) \quad (11)$$

$$\Delta G_{CS} = \Delta G_{IP} - E_g \quad (12)$$

where E_{ox} is the nucleobase (or porphyrin) oxidation potential. E_{red} is the PDI reduction potential measured in CH_2Cl_2 with a CV solvent dielectric ε_{sp}= 8.93, e is the elementary constant, r_{DA} is the donor-acceptor distance-displacement calculated from MD minima, and $r_1 = r_2 = 1/2\ r_{DA}$ are the effective ionic radii of radical ions. In line with previous calculations, we assume a moderately polar DNA environment of ε_s ~ 10.[35] E_g is the optical bandgap, directly related to the singlet excited state energy, ΔG_{CS} is the free energy for donor-acceptor charge separation, and ΔG_{CR} is the free energy for recombination back to the ground state. Isolated purine (A, G) nucleotide energy levels were taken from Refs.[36,37]

10. Absorption of Dimers

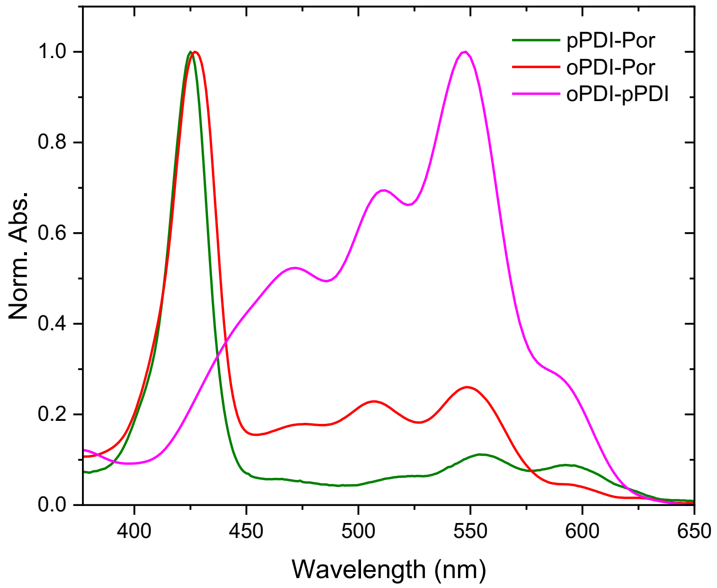

Figure S19. Intensity normalised absorption and emission spectra. 10 - 30 µM in PBS.

11. Three-Component Absorption and Emission

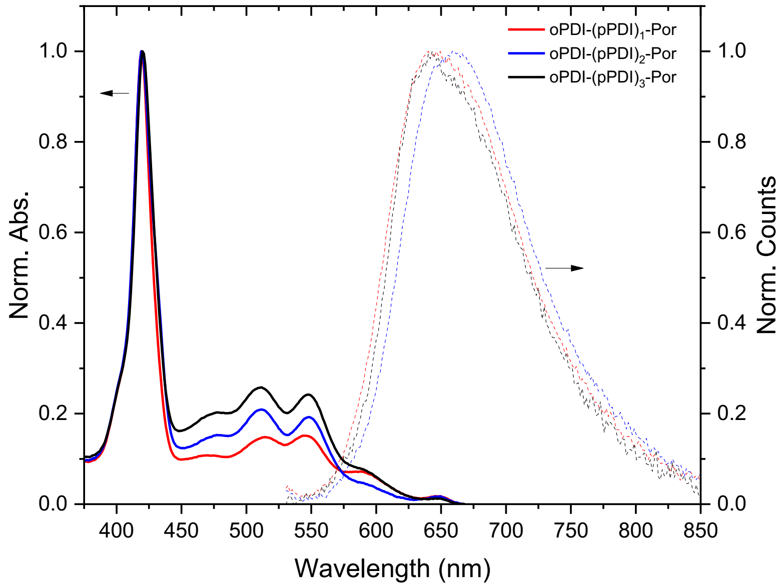

Figure S20. Intensity normalised absorption and emission spectrum. 10 - 30 µM in PBS. λ_{ex} = 520 nm.

12. Genetic Algorithm

To deconvolve the component spectra making up the transient absorption spectra in Figure 3-4 we use a genetic algorithm. This is a global analysis which produces species associated spectra and kinetics, presented in Figure S21. The full details of this approach can be found elsewhere.[38] In summary, a large population of random spectra are generated and bred to form successive generations of offspring, using a survival of the fittest approach. The best spectra are returned as optimized solutions. For a given solution, the fitness is calculated as the inverse of the sum of the squared residual with a penalty added for non-physical results. The parent spectra are selected using a tournament method with adaptive crossover. The offspring are generated using a Gaussian-function mask of random parameters. In the case of these semiconductor-DNA structures, there is significant charge transfer occurring within the instrument response time of the transient absorption experiment. In the first timeslices, there is already significant absorption from the pPDI anion at ~800 nm. Consequently, Species 1 is a mix of singlet exciton and charge transfer state, while Species 2 is dominated by the charge transfer exciton with the singlet exciton (700 nm absorption and 600 nm stimulation emission) having decayed away. The growth of Species 2 and fall of Species 1 gives the timescale on which the spectrum is evolving, and hence the timescale for the evolution of singlet exciton to charge transfer state, which varies with the size of the semiconductor-DNA stacks.

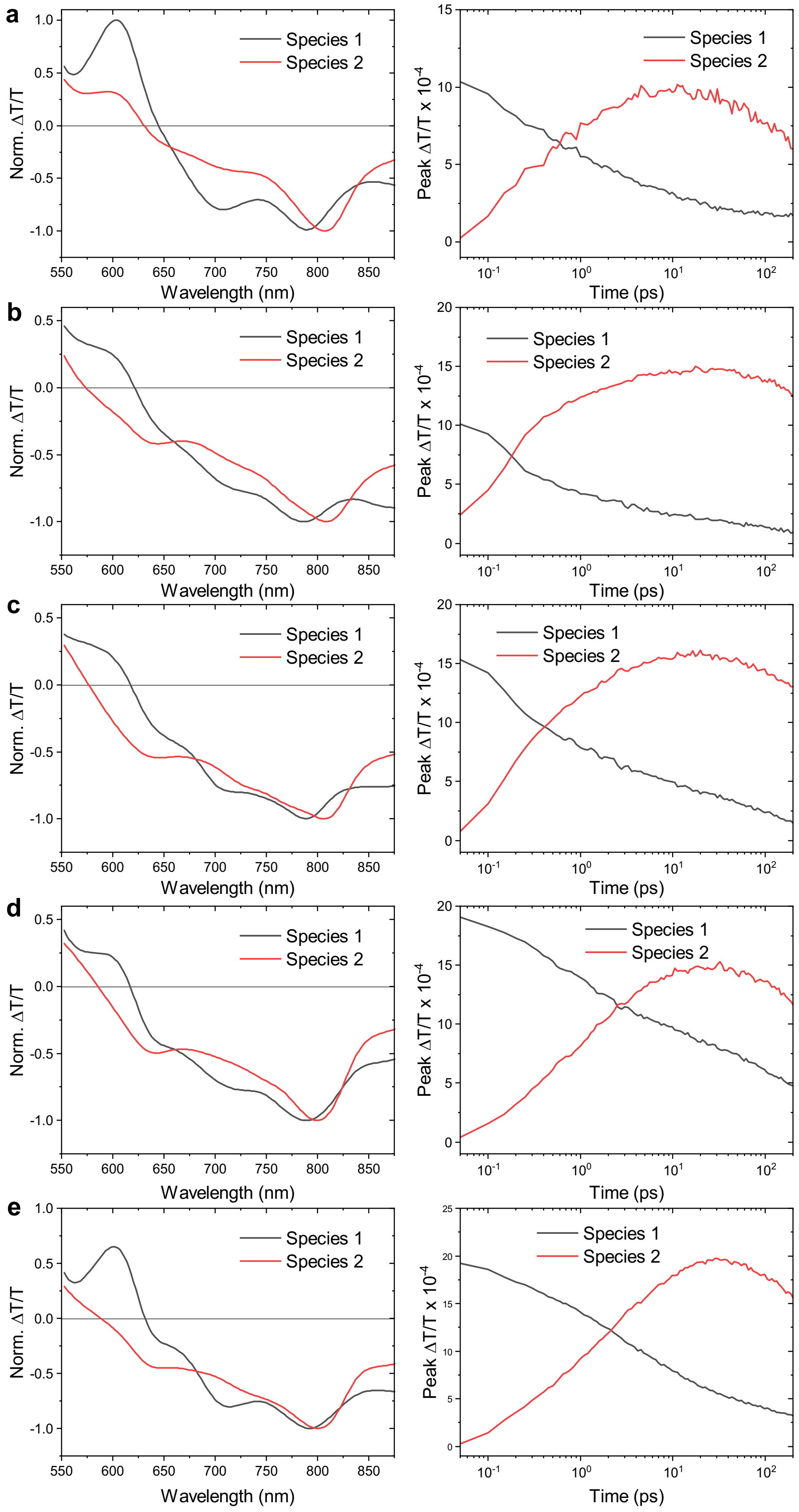

Figure S21. Genetic analysis of early-time fs-TA fitting two species and their associated decays. Species 1 = predominantly initial singlet exciton, Species 2 = radical anion. (**a**) **pPDI-Por**, (**b**) **oPDI-pPDI**, (**c**) **oPDI-(pPDI)$_1$-Por**, (**d**) **oPDI-(pPDI)$_2$-Por**, (**e**) **oPDI-(pPDI)$_3$-Por**.

13. Supplementary Note 1

DNA Assembled oPDI Photophysics

We analyzed the DNA assembled **oPDI-Por** heterodimer. By Weller analysis, we find **oPDI-Por** should form a favourable heterojunction between the electron-withdrawing oPDI and electron-donating Por (Figure S22a). However, hole transfer of a photoexcited oPDI to either G (and possibly A) is also thermodynamically allowed. Hence, we expected parasitic hole transfer to occur in **oPDI$_1$**.

MD predicts oPDI-Por cofacial stacking is unfavoured (Figure S22b). We extract a broad range of $R_{\perp}$ > 4 Å, indicating no cofacial π-π stacking compared to all other dimer configurations in the main text. Additionally, a broad distribution of $R_{\parallel}$ 5.5–8.0 Å indicates no well-defined structure between oPDI and Por. The highest probability structure snapshot in Figure S22c shows a well-separated a near perpendicular arrangement between the π-systems of both semiconductor component. However, a relatively close centre-to-centre distance is maintained due to covalent bonding by dsDNA appendages. Cofacial stacking is prevented by the steric bulk of the ortho-aryl substituents of oPDI and meso-mesityl groups of the Por component.

CAM-B3LYP simulations of the MD minimum structure (Figure S22b) yield a low[39,40] transfer integral of 1.55 meV, despite the large HOMO-HOMO and LUMO-LUMO energetic offset – highlighting the poor π-orbital overlap.

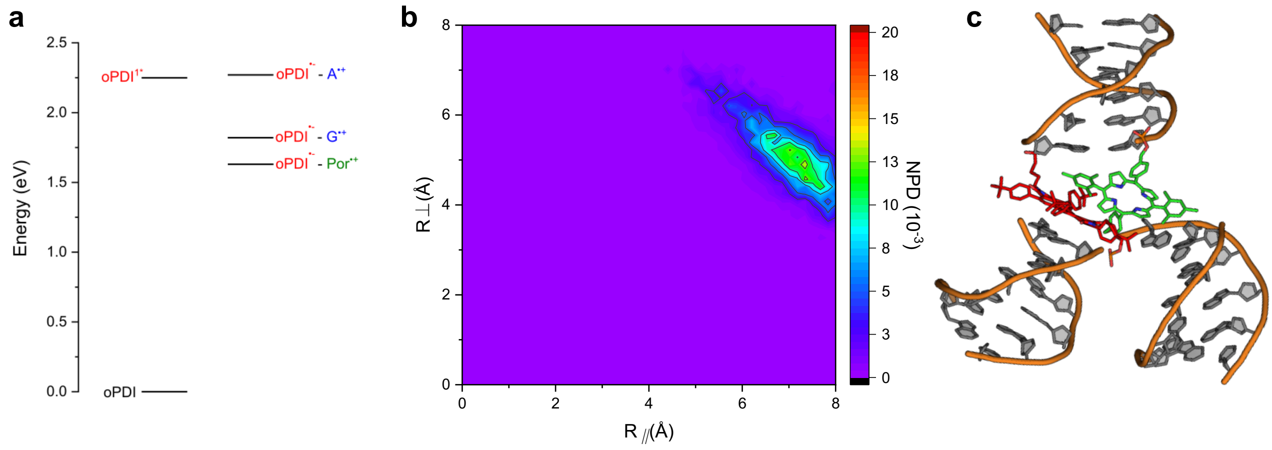

Figure S22. oPDI-Por Simulations. (**a**) Energetic Scheme comparing **oPDI** levels calculated using Weller formalism, (**b**) MD simulation of **oPDI-Por** with (**c**) high probability structure shown. NPD= normalised probability density, where are grid elements sum to 1.

14. Synthesis

Reactions were carried out with oven-dried glassware under an argon atmosphere unless otherwise stated. Reaction solvents were degassed and dry unless stated otherwise. Mesitylene and pinacolone were purchased from Sigma Aldrich, degassed, dried over CaH_2, and filtered through 0.45 µm PTFE filters before use. $TBAPF_6$ (TBA = $n\mathrm{Bu}_4\mathrm{N}^+$) electrochemical grade was purchased from Sigma Aldrich and recrystallised three times from EtOH. Anhydrous *N*,*N*-dimethylformamide (DMF) was purchased from Sigma Aldrich and was degassed prior to use. Ferrocene, used as a reference in cyclic voltammetry studies, was sublimed and stored under Ar. All other reagents were acquired from commercial sources (Tokyo Chemical Industries, Fluorochem, Alfa Aesar, or Sigma Aldrich) and used as received.

Heptathyamine and endcapping strands were purchased dry from Integrated DNA Technologies (IDT) with desalting purity and diluted in PBS (20 mM phosphate buffer, 200 mM NaCl).

All reactions were followed by analytical thin layer chromatography on cut aluminium-backed silica gel 60 F254 plates (Merck) and visualised with ultraviolet irradiation (λ = 254 or 365 nm) or permanganate staining. Flash column chromatography purification was carried out with Acros ultra-pure silica gel 40-60 µm under a positive pressure of air.

^{1}H nuclear magnetic resonance (^{1}H-NMR) spectra were recorded on Bruker Avance Ultrashield 400 or 500 spectrometers. Chemical shifts (δ) were quoted to the nearest 0.01 ppm and are referenced to the solvent residual peak. Coupling constants (*J*) were reported to the nearest 0.5 Hz. Data are reported as follows: chemical shift, multiplicity (br, broad; s, singlet; d, doublet; t, triplet; q, quartet; quin, quin; sext, sextet; m, multiplet; or a combination thereof, coupling constant(s), and integration. ^{13}C nuclear magnetic resonance (^{13}C-NMR) spectra were recorded on Bruker Avance Ultrashield 400 or 500 spectrometers. Assignments were supported by DEPT-135 spectra where necessary. Chemical shifts were quoted to the nearest 0.1 ppm and are referenced to the solvent residual peak.

Infrared spectra were recorded neat on a Perkin Elmer Spectrum One FT-IR spectrophotometer fitted with an attenuated total reflectance (ATR) sampling accessory. Absorption maxima are reported in wavenumbers (cm^{-1}).

High resolution mass spectrometry (HRMS) was carried out using Bruker ultrafleXtreme matrix-assisted laser desorption/ionization - time of flight (MALDI-TOF) or Waters LCT Premier Electrospray (ESI) instruments. Reported mass values are within the error limits of ±5 ppm.

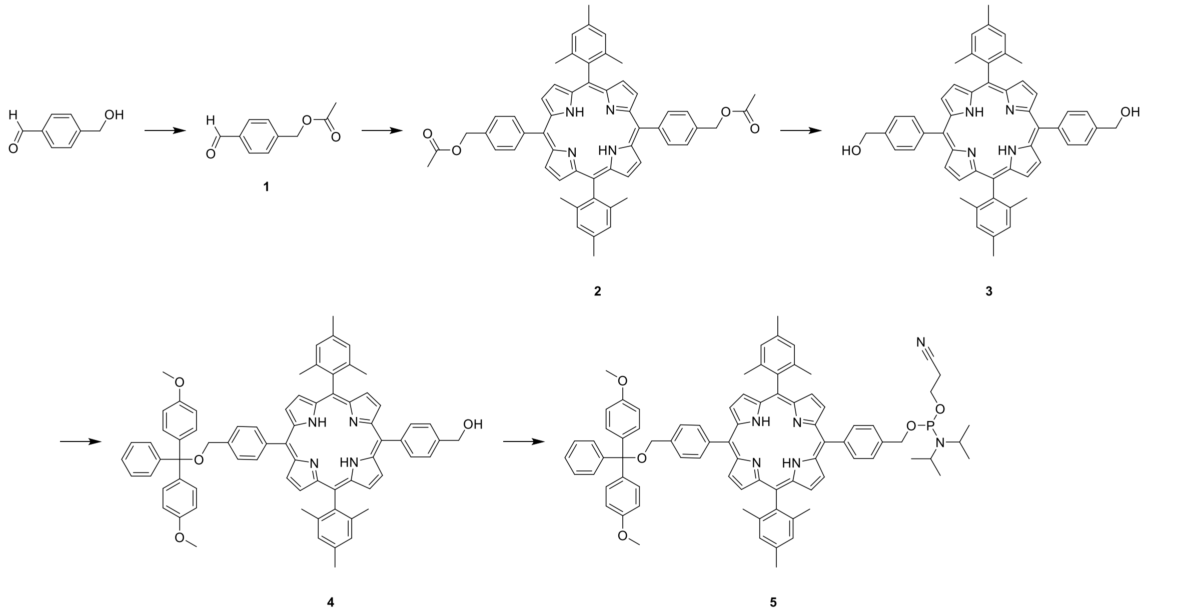

Scheme S1. Synthetic Scheme of Por-phosphoramidite.

Compound 1

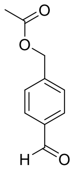

In accordance with literature.[41] 4-(Hydroxymethyl)benzaldehyde (1.00 g, 7.34 mmol, 1.00 eq.) was dissolved in pyridine (25 mL) under argon and the solution was cooled in an ice bath. Acetic anhydride (1.80 mL) was added and the ice bath was removed. The reaction mixture was stirred at r.t. overnight and then poured into water (250 mL). The mixture was extracted with DCM (4 × 75 mL) and the extracts were combined, washed with water (2 × 50 mL), dried over $MgSO_4$ and filtered. The solvent was removed under reduced pressure. Residual acetic anhydride was then distilled off by coevaporation with toluene on a rotary evaporator (2 × 50 mL) to afford 4-(acetyloxymethyl)benzaldehyde **1** as a colourless oil (1.21 g, 6.77 mmol, 92%).

^1H NMR (400 MHz, $CDCl_3$) δ 10.02 (s, 1H), 7.89 (d, J = 8.2 Hz, 2H), 7.52 (d, J = 8.2 Hz, 2H), 5.19 (s, 2H), 2.15 (s, 3H).

Compound 2

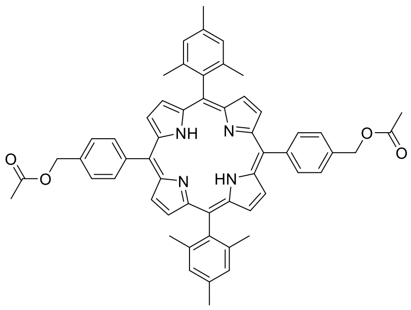

5-Mesityl dipyrromethene[42] (2.30 g, 5.78 mmol, 1.00 eq.) and **1** (1.53 g, 5.78 mmol, 1.00 eq.) were dissolved in dry DCM (1000 mL) under argon and the resulting solution was degassed for 1 h. Trifluoroacetic acid (1.20 mL, 7.06 mmol, 1.22 eq.) was added and the mixture was stirred at r.t. for 3 h. DDQ (2.36 g, 10.4 mmol, 1.47 eq.) was added in a single portion and stirring was continued at r.t. overnight. The mixture was then heated to reflux for 1 h before it was cooled to r.t. and poured onto a plug of silica gel packed with hexane (⌀ 12 cm × 2 cm). It was eluted with further DCM (1000 mL) and the purple band was collected. Et_3N (5 mL) was added and the solvent was removed under reduced pressure. The residue was then further purified by flash chromatography (silica gel, DCM/hexane 1:9-1:0) to afford **2** as a purple powder sufficiently pure for the next step (730 mg, 0.87 mmol, 30%).

^1H NMR (400 MHz, $CDCl_3$) δ 8.79 (d, J = 4.7 Hz, 4H), 8.69 (d, J = 4.7 Hz, 4H), 8.23 (d, J = 7.9 Hz, 4H), 7.73 (d, J = 7.9 Hz, 4H), 7.29 (s, 4H), 5.47 (s, 4H), 2.64 (s, 6H), 2.29 (s, 6H), 1.84 (s, 12H), -2.63 (s, 2H).

HRMS (ESI): m/z 843.3883 [M–H]$^+$. Calcd. for $C_{56}H_{51}N_4O_4^+$; Found 843.3905.

Compound 3

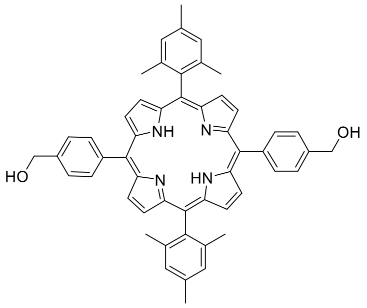

2 (713 mg, 0.85 mmol, 1.00 eq.) and NaOH (676 mg, 16.9 mmol, 19.9 eq.) were combined in 1:1 THF/ water *v*/*v* (200 mL) and the mixture stirred at r.t. under air for 72 h. The solvent volume was reduced to precipitate **9** as a shiny purple powder that was isolated via filtration and washed sequentially with MeOH (2 x 5 mL) and diethyl ether (3 x 1 mL) (640 mg, 0.84 mmol, 99%). The material was sufficiently pure for the next step.

^1H NMR (400 MHz, $CDCl_3$) δ 8.79 (d, J = 4.7 Hz, 4H), 8.69 (d, J = 4.7 Hz, 4H), 8.22 (d, J = 7.9 Hz, 4H), 7.75 (d, J = 7.9 Hz, 4H), 7.28 (s, 4H), 5.07 (d, J = 5.8 Hz, 4H), 3.49-3.47 (m, 4H), 2.63 (s, 2H), 1.84 (s, 12H), -2.62 (s, 2H).

FT-IR (ATR) ν = 3472 (br w, O-H), 3295 (br w, N-H), 2916 (w, C-H), 2853 (w, C-H), 1610 (w), 1558 (w), 1470 (m), 1401 (m), 1375 (m), 1347 (m), 1211 (m), 1186 (m) 1018 (m) cm^{-1}.

HRMS (ESI): m/z 759.3702 [M–H]$^+$. Calcd. for $C_{52}H_{47}N_4O_2^+$; Found 759.3694.

Compound 4

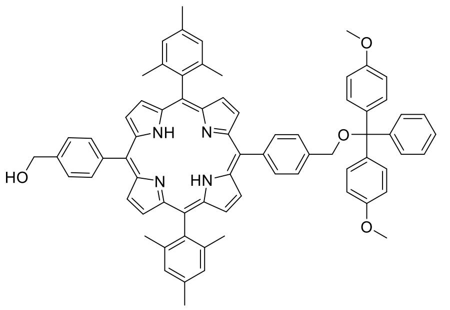

3 (560 mg, 0.97 mmol, 1.00 eq.), DMAP (15 mg, 0.12 mmol, 0.12 eq.), and anhydrous Et_3N (418 µL, 3 mmol, 3.09 eq.) were dissolved in anhydrous DCM (425 mL) under argon at r.t. for 30 mins. 4,4′-Dimethoxytrityl chloride (458 mg, 1.35 mmol, 1.39 eq.) was added as a solid in three portions over 1 h against argon flow. The reaction was left to continue for another 2 h, and then quenched with MeOH (1 mL). The solvent was reduced to a 40 mL slurry and re-diluted in 150 mL $CHCl_3$. The organic phase was washed with sat. $NaHCO_3$, brine, then dried over $MgSO_4$, filtered, and concentrated under reduced pressure. The solid was purified by column chromatography (silica gel, $CHCl_3$/EtOAc/Et_3N 95:5:3-70:30:3) the second dark red band (containing product) was collected. The first (starting material) and third red bands (bis-DMTr product) were collected and dried. Bis-DMTr product was converted back to starting material by stirring in DCM:TFA (1/1: vol/vol) for 10 mins. A red purple was precipitated by addition of hexanes, collected by filtration, washed with H2O, and dried. The combined starting materials were subjected to repeat reaction conditions and purification a further two times. Trituration in DCM/MeOH yielded **4** as a purple solid (515 mg, 0.49 mmol, 50%).

^{1}H NMR (400 MHz, $CDCl_3$) δ 8.84 (d, J = 4.8 Hz, 2H), 8.80 (d, J = 4.8 Hz, 2H), 8.69 (m, 4H), 8.24-8.18 (m, 4H), 7.77-7.74 (m, 4H), 7.70-7.68 (m, 2H), 7.60-7.56 (m, 4H), 7.40 (t, J = 7.2 Hz, 2H), 7.32-7.29 (m, 5H), 6.96-6.94 (m, 4H), 5.07-5.06 (m, 2H), 4.56 (s, 2H), 3.85 (s, 6H), 2.64 (s, 6H), 1.85 (s, 12H), -2.60 (br s, 2H).

^{13}C NMR (101 MHz, $CDCl_3$) δ 158.6, 145.2, 141.5, 140.6, 139.4, 138.8, 138.5, 137.7, 136.4, 134.7, 134.4, 130.3, 128.4, 128.0, 127.8, 126.9, 125.3, 125.2, 125.1, 119.4, 118.9, 118.3, 113.3, 86.7, 65.7, 65.5, 55.3, 21.7, 21.5.

R_f = 0.52 (DCM/EtOAc 9:1).

FT-IR (ATR) ν = 3477 (br w, O-H), 2916 (w, C-H), 2854 (w, C-H), 1610 (w), 1558 (w), 1475 (m), 1437 (m), 1400 (m), 1380 (m), 1349 (m), 1210 (m), 1018 (m) cm^{-1}.

HRMS (MALDI-TOF) m/z: $[M + H]^+$ Calc for $C_{73}H_{65}N_4O_4^+$ 1061.5000; Found 1061.5020.

Compound 5

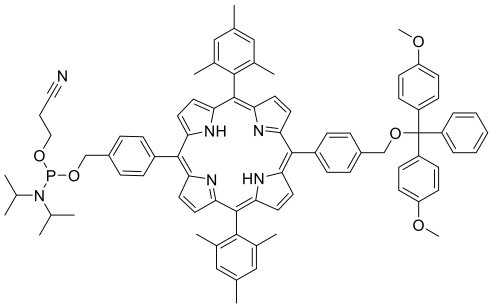

In an oven-dried, argon-filled round bottom flask with 4 Å molecular sieves; **4** (170 mg, 0.16 mmol, 1.00 eq.) was dissolved in anhydrous DCM (7 mL), followed by degassed DIPEA (111 µL, 0.64 mmol, 4.00 eq.). Degassed 2-cyanoethyl *N*,*N*-diisopropylchlorophosphoramidite (105 µL, 0.47 mmol, 2.94 eq.) was added by injection and left to gently stir for 45 mins under argon. The mixture was concentrated by gas flow, precipitated with anhydrous hexane (10 mL), and supernatant removed by cannula filtration under argon. The solids were washed again with anhydrous (10 mL) and filtered by cannula under argon until dry. The solids were dissolved in anhydrous DCM:MeCN / 2:1 (4 mL) and transferred by cannula to an oven dried, argon filled ampule, and used immediately for SPOS.

R_f = 0.22 (DCM/EtOAc 9:1)

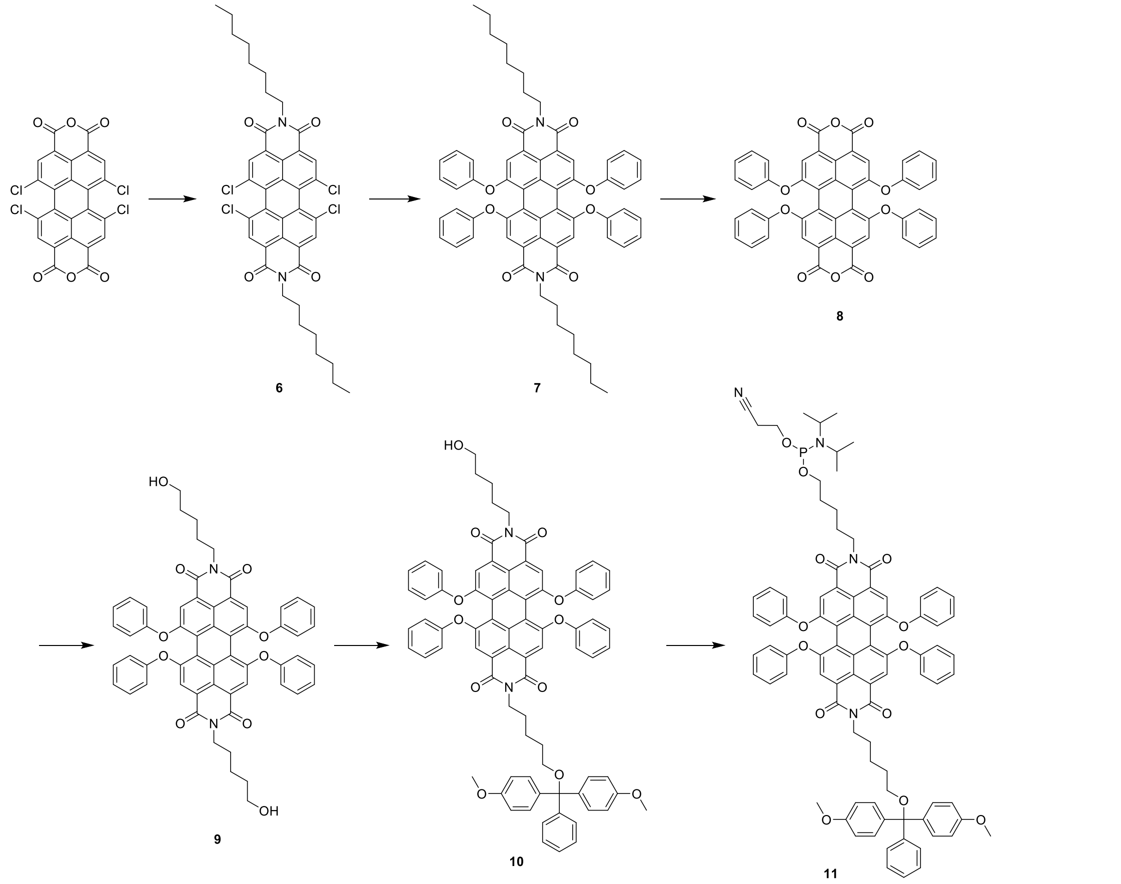

Scheme S2. Synthetic Scheme of pPDI-phosphoramidite.

Compound 6

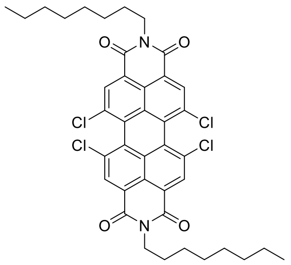

In accordance with literature.[43] *n*-octylamine, (7.8 mL, 47 mmol, 4.9 eq.) was added dropwise to a suspension of 1,6,7,12-Tetrachloroperylene tetracarboxylic acid dianhydride (5 g, 9.42 mmol, 1.0 eq) in propionic acid (90 mL) and heated under reflux overnight. The mixture was cooled, the orange precipitate collected by filtration, and washed with water. The solid was dissolved in DCM, washed with brine, dried over $MgSO_4$, filtered, and concentrated under reduced pressure. The solid was purified by column chromatography (silica gel, DCM) to yield **6** as an orange powder (6.59 g, 8.76 mmol, 93%).

^{1}H NMR (400 MHz, $CDCl_3$) δ 8.68 (s, 4H), 4.21 (t, *J* = 7.65 Hz, 4H), 1.75 (quin, *J* = 7.48 Hz, 4H), 1.47-1.26 (m, 20H), 0.88 (t, *J* = 6.79 Hz, 6H).

^{13}C NMR (101 MHz, $CDCl_3$) δ 162.3, 135.7, 132.9, 131.4, 128.6, 128.6, 123.3, 50.0, 31.8, 29.3, 29.2, 28.1, 27.1, 14.1.

R_f = 0.91 ($CHCl_3$)

Compound 7

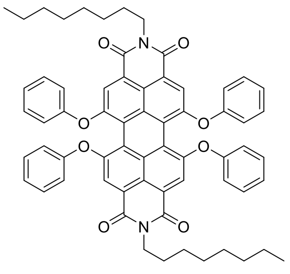

In accordance with literature.[44] **6** (6.77 g, 9 mmol, 1 eq.), phenol (33.8 g, 360 mmol, 40 eq.), K_2CO_3 (24.8 g, 180 mmol, 40 eq.), and NMP (200 mL) were mixed under argon and heated overnight at 130 °C. The reaction was cooled, dropped into 1 M HCl (300 mL), and stirred for 3 h. The purple precipitate was collected by filtration and washed with water. The solid was purified by column chromatography (silica gel, DCM/Heptane 4:1) to yield **7** as a purple powder (6.72 g, 6.83 mmol, 76%).

^{1}H NMR (400 MHz, $CDCl_3$) δ 8.19 (s, 4H), 7.26 (t, J = 7.83 Hz, 8H), 7.13-7.08 (m, 4H), 6.95-9.93 (m, 8H), 4.11-4.07 (m, 4H), 1.66 (quin, J = 7.20 Hz, 4H), 1.36-1.23 (m, 20H), 0.85 (t, J = 6.94 Hz, 6H).

^{13}C NMR (101 MHz, $CDCl_3$) δ 163.3, 155.8, 155.3, 132.8, 130.0, 124.6, 122.7, 120.5, 120.0, 119.6, 40.7, 31.8, 29.3, 29.2, 28.1, 27.1, 22.6, 14.1.

R_f = 0.92 (DCM + 5 % Heptane).

Compound 8

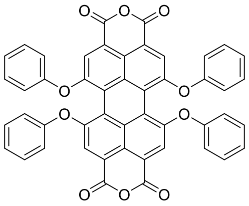

In accordance with literature.[45] KOH (4.76 g, 85 mmol, 12.7 eq.) was added to a suspension of **7** (6.68g, 6.8 mmol, 1.0 eq.) in ^{t}BuOH (200 mL) and water (2 mL), the mixture was heated at reflux overnight under argon. The reaction was cooled to 30 °C before acetic acid (200 mL) and 2 M HCl (100 mL) were added slowly, and stirred for 3 h. The precipitate was collected by filtration and washed with water. The solid was dissolved in $CHCl_3$, washed with water then brine, dried over $MgSO_4$, filtered, and concentrated under reduced pressure to yield **8** as a purple powder (3.46 g, 4.56 mmol, 67%).

^{1}H NMR (400 MHz, $CDCl_3$) δ 8.17 (s, 4H), 7.32-7.28 (m, 8H), 7.18-7.15 (m, 4H), 6.60-6.93 (m, 8H).

^{13}C NMR (101 MHz, $CDCl_3$) δ 159.6, 156.4, 154.7, 133.4, 130.3, 123.5, 121.5, 121.4, 121.3, 120.1, 120.0, 118.9.

R_f = 0.38 (DCM).

Compound 9

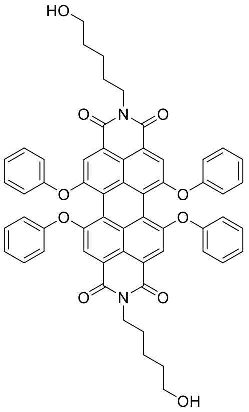

8 (3.4 g, 4.5 mmol, 1.0 eq.), $Zn(OAc)_2{\cdot}2H_2O$ (3.96 g, 18 mmol, 4.0 eq.), 5-amino-1-pentanol (1.85 g, 18 mmol, 4.0 eq.), and imidazole (30 g) were mixed under argon at 100 °C for 7 h. The reaction was cooled, the crude dissolved in $CHCl_3$ (200 mL) and 0.5 M HCl (100 mL) and stirred for 3 h. The mixture was diluted with water and product extracted with $CHCl_3$. The combined organic phases were washed with brine, dried over $MgSO_4$, filtered, and concentrated under reduced pressure. The solid was purified by column chromatography (silica gel, DCM/MeOH 100:5) to yield **9** as a purple powder (2.60 g, 2.79 mmol, 62%).

^{1}H NMR (400 MHz, $CDCl_3$) δ 8.18 (s, 4H), 7.26 (t, J = 7.57 Hz, 8H), 7.11 (t, J = 7.36 Hz, 4H), 6.95-6.92 (m, 8H), 4.12 (t, J = 7.21 Hz, 4H), 3.64-3.60 (m, 6H), 1.71 (quin, J = 7.26 Hz, 4H), 1.60 (quin, J = 7.84 Hz, 4H), 1.48-1.39 (m, 4H).

^{13}C NMR (101 MHz, $CDCl_3$) δ 163.3, 155.9, 155.3, 132.8, 130.0, 124.6, 122.6, 120.5, 120.0, 119.6, 62.7, 40.4, 32.3, 29.2, 27.8, 23.2.

R_f = 0.25 (DCM + 5 % MeOH).

FT-IR (ATR) ν = 3333 (br m, O-H), 3039 (w, C-H), 2916 (m, C-H), 1692 (m, C=O), 1646 (s, C=O), 1585 (s, C=O), 1560 (m), 1509 (w), 1486 (s), 1437 (w), 1412 (m), 1355 (m), 1286 (s, C-O), 1212 (s, C-O), 1163 (m), 1110 (m), 1070 (w) cm^{-1}.

HRMS (MALDI-TOF) m/z: $[M + H]^+$ Calc for $C_{58}H_{47}N_2O_{10}^+$ 931.3225; Found 931.3209.

Compound 10

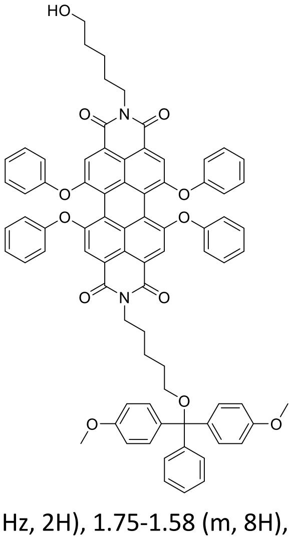

9 (978 mg, 1.06 mmol, 1.0 eq.), DMAP (15 mg, 0.12 mmol, 0.1 eq.), Et_3N (442 µL, 3.18 mmol, 3.0 eq.), and DCM (125 mL) were stirred under argon at r.t.. 4,4′-Dimethoxytrityl chloride (536 mg, 1.58 mmol, 1.5 eq.) was added as a solid in three portions over 1 hour against argon flow. The reaction was left to continue for another 2 h, then quenched with MeOH (1 mL), and diluted with 50 mL of DCM. The organic phase was washed with sat. NaHCO3, brine, then dried over $MgSO_4$, filtered, and concentrated under reduced pressure. The solid was purified by column chromatography (silica gel, DCM/EtOAc/Et_3N 85:15:3 → 70:30:3) the second purple band (containing product) was collected. The first and third purple bands were collected, dried, and subjected to repeat reaction conditions and purification a further two times. Trituration in DCM/EtOH yielded **10** as a purple solid (620 mg, 0.50 mmol, 47%).

^{1}H NMR (400 MHz, $CDCl_3$) δ 8.19-1.17 (m, 4H), 7.39-7.37 (m, 2H), 7.28-7.26 (m, 3H), 7.25-7.08 (m, 14H), 6.95-6.91 (m, 8H), 6.79-6.75 (m, 4H), 6.77 (d, J = 8.58 Hz, 2H), 4.14-4.07 (m, 4H), 3.80 (s, 1H), 3.73 (s, 6H), 3.62 (q, J = 6.76 Hz, 2H), 3.00 (t, J = 6.38 Hz, 2H), 1.75-1.58 (m, 8H), 1.48-1.40 (m, 4H).

^{13}C NMR (101 MHz, $CDCl_3$) δ 163.3, 158.6, 128.2, 155.9, 155.3, 139.5, 136.6, 123.8, 1.30.0, 129.1, 128.1, 127.9, 127.8, 127.6, 127.1, 126.5, 122.6, 120.5, 120.0, 119.6, 113.2, 112.9, 85.6, 62.7, 55.3, 55.1, 40.4, 32.3, 27.8, 23.2.

R_f = 0.51 (DCM + 3 %EtOAc).

FT-IR (ATR) v = 3389 (br w, O-H), 2915 (m, C-H), 2901 (m, C-H), 2868 (m, C-H), 1692 (s, C=O), 1651 (s, C=O), 1592 (s, C=O), 1508 (w), 1438 (m), 1401 (m), 1361 (s), 1342 (s), 1247 (m) 1217 (w), 1180 (w), 1060 (s) cm^{-1}.

HRMS (MALDI-TOF) m/z: $[M - H]^+$ Calc for $C_{79}H_{65}N_2O_{12}$ 1233.4459; Found 1233.4397.

Compound 11

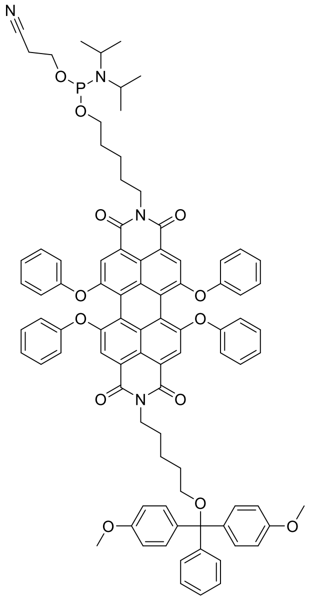

In an oven-dried and thoroughly heat-dried, argon-filled round bottom flask with 4 Å molecular sieves, **10** (198 mg, 0.16 mmol, 1.0 eq.) was dissolved in dry DCM (7 mL), followed by DIPEA (111 µL, 0.64 mmol, 4.0 eq.). 2-Cyanoethyl *N*,*N*-diisopropylchlorophosphoramidite (105 µL, 0.47 mmol, 3.0 eq.) was added by injection and left to gently stir for 45 mins under argon. The mixture was concentrated by gas flow, precipitated with dry MeCN (10 mL), and supernatant removed by cannula filtration under argon. The solids were washed again with MeCN (10 mL) and filtered by cannula under argon until dry. The solids were dissolved in DCM (4 mL) and transferred by cannula to an oven dried, argon filled ampule, and used immediately for SPOS.

R_f = 0.44 (DCM/EtOAc 9:1)

15. References

(1) De Mello, J. C.; Wittmann, H. F.; Friend, R. H. An Improved Experimental Determination of External Photoluminescence Quantum Efficiency. *Adv. Mater.* **1997**, *9* (3), 230–232. https://doi.org/10.1002/adma.19970090308.

(2) Neese, F. Software Update: The ORCA Program System, Version 4.0. *WIREs Comput Mol Sci* **2018**, *8* (1). https://doi.org/10.1002/wcms.1327.

(3) Neese, F. The ORCA Program System. *WIREs Comput Mol Sci* **2012**, *2* (1), 73–78. https://doi.org/10.1002/wcms.81.

(4) Yanai, T.; Tew, D. P.; Handy, N. C. A New Hybrid Exchange–Correlation Functional Using the Coulomb-Attenuating Method (CAM-B3LYP). *Chemical Physics Letters* **2004**, *393* (1–3), 51–57. https://doi.org/10.1016/j.cplett.2004.06.011.

(5) Weigend, F.; Ahlrichs, R. Balanced Basis Sets of Split Valence, Triple Zeta Valence and Quadruple Zeta Valence Quality for H to Rn: Design and Assessment of Accuracy. *Phys. Chem. Chem. Phys.* **2005**, *7* (18), 3297. https://doi.org/10.1039/b508541a.

(6) Madjet, M. E.; Abdurahman, A.; Renger, T. Intermolecular Coulomb Couplings from Ab Initio Electrostatic Potentials: Application to Optical Transitions of Strongly Coupled Pigments in Photosynthetic Antennae and Reaction Centers. *J. Phys. Chem. B* **2006**, *110* (34), 17268–17281. https://doi.org/10.1021/jp0615398.

(7) Zhang, J. Libreta : Computerized Optimization and Code Synthesis for Electron Repulsion Integral Evaluation. *J. Chem. Theory Comput.* **2018**, *14* (2), 572–587. https://doi.org/10.1021/acs.jctc.7b00788.

(8) Lu, T.; Chen, F. Multiwfn: A Multifunctional Wavefunction Analyzer. *J. Comput. Chem.* **2012**, *33* (5), 580–592. https://doi.org/10.1002/jcc.22885.

(9) Gorman, J.; Orsborne, S. R. E.; Sridhar, A.; Pandya, R.; Budden, P.; Ohmann, A.; Panjwani, N. A.; Liu, Y.; Greenfield, J. L.; Dowland, S.; Gray, V.; Ryan, S. T. J.; De Ornellas, S.; El-Sagheer, A. H.; Brown, T.; Nitschke, J. R.; Behrends, J.; Keyser, U. F.; Rao, A.; Collepardo-Guevara, R.; Stulz, E.; Friend, R. H.; Auras, F. Deoxyribonucleic Acid Encoded and Size-Defined π-Stacking of Perylene Diimides. *Journal of the American Chemical Society* **2021**, *144* (1), 368–376. https://doi.org/10.1021/jacs.1c10241.

(10) Pettersen, E. F.; Goddard, T. D.; Huang, C. C.; Couch, G. S.; Greenblatt, D. M.; Meng, E. C.; Ferrin, T. E. UCSF Chimera: A Visualization System for Exploratory Research and Analysis. *J. Comput. Chem.* **2004**, *25* (13), 1605–1612. https://doi.org/10.1002/jcc.20084.

(11) Vanquelef, E.; Simon, S.; Marquant, G.; Garcia, E.; Klimerak, G.; Delepine, J. C.; Cieplak, P.; Dupradeau, F. Y. R.E.D. Server: A Web Service for Deriving RESP and ESP Charges and Building Force Field Libraries for New Molecules and Molecular Fragments. *Nucleic Acids Research* **2011**, *39*, 511–517. https://doi.org/10.1093/nar/gkr288.

(12) Bayly, C. I.; Cieplak, P.; Cornell, W.; Kollman, P. A. A Well-Behaved Electrostatic Potential Based Method Using Charge Restraints for Deriving Atomic Charges: The RESP Model. *Journal of Physical Chemistry* **1993**, *97* (40), 10269–10280. https://doi.org/10.1021/j100142a004.

(13) Hornak, V.; Abel, R.; Okur, A.; Strockbine, B.; Roitberg, A.; Simmerling, C. Comparison of Multiple Amber Force Fields and Development of Improved Protein Backbone Parameters. *Proteins: Structure, Function and Bioinformatics* **2006**, *65* (3), 712–725. https://doi.org/10.1002/prot.

(14) Lindorff-Larsen, Kresten; Piana, Stefano; Palmo, Kim; Marakis, Paul; Klepeis, John L.; Dror, Ron O.; Shaw, David E. Improved Side-chain Torsion Potentials for the Amber Ff99SB Protein Force Field.Pdf. *Proteins* **2010**, *78* (8), 1813–2004.

(15) Ivani, I.; Dans, P. D.; Noy, A.; Pérez, A.; Faustino, I.; Hospital, A.; Walther, J.; Andrio, P.; Goñi, R.; Balaceanu, A.; Portella, G.; Battistini, F.; Gelpí, J. L.; González, C.; Vendruscolo, M.; Laughton, C. A.; Harris, S. A.; Case, D. A.; Orozco, M. Parmbsc1: A Refined Force Field for DNA Simulations. *Nature Methods* **2015**, *13* (1), 55–58. https://doi.org/10.1038/nmeth.3658.

(16) Wang, Jumei; Wang, Wei; Kollman, Peter A.; Case, David A. Automatic Atom Type and Bond Type Perception in Molecular Mechanical Calculations. *J. Mol. Graph. 25* (2), 247–260.

(17) Wang, J. M.; Wolf, R. M.; Caldwell, J. W.; Kollman, P. A.; Case, D. A. Development and Testing of a General Amber Force Field. *Journal of Computational Chemistry* **2004**, *25* (9), 1157–1174. https://doi.org/10.1002/jcc.20035.

(18) Hanwell, M. D.; Curtis, D. E.; Lonie, D. C.; Vandermeersch, T.; Zurek, E.; Hutchison, G. R. Avogadro: An Advanced Semantic Chemical Editor, Visualization, and Analysis Platform. *J Cheminform* **2012**, *4* (1), 17. https://doi.org/10.1186/1758-2946-4-17.

(19) Case, D. A.; Cheatham, T. E.; Darden, T.; Gohlke, H.; Luo, R.; Merz, K. M.; Onufriev, A.; Simmerling, C.; Wang, B.; Woods, R. J. The Amber Biomolecular Simulation Programs. *Journal of Computational Chemistry* **2005**, *26* (16), 1668–1688. https://doi.org/10.1002/jcc.20290.

(20) Jorgensen, W. L.; Chandrasekhar, J.; Madura, J. D.; Impey, R. W.; Klein, M. L. Comparison of Simple Potential Functions for Simulating Liquid Water. *Journal of Chemical Physics* **1983**, *79* (2), 926–935. https://doi.org/10.1063/1.445869.

(21) Joung, I. S.; Cheatham, T. E. Determination of Alkali and Halide Monovalent Ion Parameters for Use in Explicitly Solvated Biomolecular Simulations. *Journal of Physical Chemistry B* **2008**, *112* (30), 9020–9041. https://doi.org/10.1021/jp8001614.

(22) Abraham, M. J.; Murtola, T.; Schulz, R.; Pall, S.; Smith, J. C.; Hess, B.; Lindah, E. Gromacs: High Performance Molecular Simulations through Multi-Level Parallelism from Laptops to Supercomputers. *SoftwareX* **2015**, *1* (2), 19–25. https://doi.org/10.1016/j.softx.2015.06.001.

(23) Shirts, M. R.; Klein, C.; Swails, J. M.; Yin, J.; Gilson, M. K.; Mobley, D. L.; Case, D. A.; Zhong, E. D. Lessons Learned from Comparing Molecular Dynamics Engines on the SAMPL5 Dataset. *J Comput Aided Mol Des* **2017**, *31* (1), 147–161. https://doi.org/10.1007/s10822-016-9977-1.

(24) Bussi, G.; Donadio, D.; Parrinello, M. Canonical Sampling through Velocity Rescaling. *Journal of Chemical Physics* **2007**, *126* (1). https://doi.org/10.1063/1.2408420.

(25) Parrinello, M.; Rahman, A. Polymorphic Transitions in Single Crystals: A New Molecular Dynamics Method. *Journal of Applied Physics* **1981**, *52* (12), 7182–7190. https://doi.org/10.1063/1.328693.

(26) York, D. M.; Darden, T. A.; Pedersen, L. G. The Effect of Long-range Electrostatic Interactions in Simulations of Macromolecular Crystals: A Comparison of the Ewald and Truncated List Methods. *The Journal of Chemical Physics* **1993**, *99* (10), 8345–8348. https://doi.org/10.1063/1.465608.

(27) Bonomi, M.; Branduardi, D.; Bussi, G.; Camilloni, C.; Provasi, D.; Raiteri, P.; Donadio, D.; Marinelli, F.; Pietrucci, F.; Broglia, R. A.; Parrinello, M. PLUMED: A Portable Plugin for Free-Energy Calculations with Molecular Dynamics. *Computer Physics Communications* **2009**, *180* (10), 1961–1972. https://doi.org/10.1016/j.cpc.2009.05.011.

(28) Tribello, G. A.; Bonomi, M.; Branduardi, D.; Camilloni, C.; Bussi, G. PLUMED 2: New Feathers for an Old Bird. *Computer Physics Communications* **2014**, *185* (2), 604–613. https://doi.org/10.1016/j.cpc.2013.09.018.

(29) Michaud-Agrawal, Naveen; Denning, Elizabeth J.; Woolf, Thomas B.; Beckstein, Oliver. MDAnalysis A Toolkit for the Analysis of Molecular Dynamics Simulations.Pdf. *J Comput. Chem. 32* (10), 205–2338.

(30) Laio, A.; Parrinello, M. Escaping Free-Energy Minima. *Proceedings of the National Academy of Sciences of the United States of America* **2002**, *99* (20), 12562–12566. https://doi.org/10.1073/pnas.202427399.

(31) Barducci, A.; Bussi, G.; Parrinello, M. Well-Tempered Metadynamics: A Smoothly Converging and Tunable Free-Energy Method. *Physical Review Letters* **2008**, *100* (2). https://doi.org/10.1103/PhysRevLett.100.020603.

(32) Tiwary, P.; Parrinello, M. A Time-Independent Free Energy Estimator for Metadynamics. *Journal of Physical Chemistry B* **2015**, *119* (3), 736–742. https://doi.org/10.1021/jp504920s.

(33) Bonomi, M.; Barducci, A.; Parrinello, M. Reconstructing the Equilibrium Boltzmann Distribution from Well-tempered Metadynamics.Pdf. *Journal of Computational Chemistry 30* (11), 1615–1747.

(34) Virtanen, P.; Gommers, R.; Oliphant, T. E.; Haberland, M.; Reddy, T.; Cournapeau, D.; Burovski, E.; Peterson, P.; Weckesser, W.; Bright, J.; van der Walt, S. J.; Brett, M.; Wilson, J.; Millman, K. J.; Mayorov, N.; Nelson, A. R. J.; Jones, E.; Kern, R.; Larson, E.; Carey, C. J.; Polat, İ.; Feng, Y.; Moore, E. W.; VanderPlas, J.; Laxalde, D.; Perktold, J.; Cimrman, R.; Henriksen, I.; Quintero, E. A.; Harris, C. R.; Archibald, A. M.; Ribeiro, A. H.; Pedregosa, F.; van Mulbregt, P.; Vijaykumar, A.; Bardelli, A. P.; Rothberg, A.; Hilboll, A.; Kloeckner, A.; Scopatz, A.; Lee, A.; Rokem, A.; Woods, C. N.; Fulton, C.; Masson, C.; Häggström, C.; Fitzgerald, C.; Nicholson, D. A.; Hagen, D. R.; Pasechnik, D. V.; Olivetti, E.; Martin, E.; Wieser, E.; Silva, F.; Lenders, F.; Wilhelm, F.; Young, G.; Price, G. A.; Ingold, G. L.; Allen, G. E.; Lee, G. R.; Audren, H.; Probst, I.; Dietrich, J. P.; Silterra, J.; Webber, J. T.; Slavič, J.; Nothman, J.; Buchner, J.; Kulick, J.; Schönberger, J. L.; de Miranda Cardoso, J. V.; Reimer, J.; Harrington, J.; Rodríguez, J. L. C.; Nunez-Iglesias, J.; Kuczynski, J.; Tritz, K.; Thoma, M.; Newville, M.; Kümmerer, M.; Bolingbroke, M.; Tartre, M.; Pak, M.; Smith, N. J.; Nowaczyk, N.; Shebanov, N.; Pavlyk, O.; Brodtkorb, P. A.; Lee, P.; McGibbon, R. T.; Feldbauer, R.; Lewis, S.; Tygier, S.; Sievert, S.; Vigna, S.; Peterson, S.; More, S.; Pudlik, T.; Oshima, T.; Pingel, T. J.; Robitaille, T. P.; Spura, T.; Jones, T. R.; Cera, T.; Leslie, T.; Zito, T.; Krauss, T.; Upadhyay, U.; Halchenko, Y. O.; Vázquez-Baeza, Y. SciPy 1.0: Fundamental Algorithms for Scientific Computing in Python. *Nature Methods* **2020**, *17* (3), 261–272. https://doi.org/10.1038/s41592-019-0686-2.

(35) Carmieli, R.; Zeidan, T. A.; Kelley, R. F.; Mi, Q.; Lewis, F. D.; Wasielewski, M. R. Excited State, Charge Transfer, and Spin Zynamics in DNA Hairpin Conjugates with Perylenediimide Hairpin Linkers'. *Journal of Physical Chemistry A* **2009**, *113* (16), 4691–4700. https://doi.org/10.1021/jp900230q.
(36) Caruso, T.; Carotenuto, M.; Vasca, E.; Peluso, A. Direct Experimental Observation of the Effect of the Base Pairing on the Oxidation Potential of Guanine. *Journal of the American Chemical Society* **2005**, *127* (43), 15040–15041. https://doi.org/10.1021/ja055130s.
(37) Caruso, T.; Capobianco, A.; Peluso, A. The Oxidation Potential of Adenosine and Adenosine-Thymidine Base Pair in Chloroform Solution. *Journal of the American Chemical Society* **2007**, *129* (49), 15347–15353. https://doi.org/10.1021/ja076181n.
(38) Gélinas, S.; Rao, A.; Kumar, A.; Smith, S. L.; Chin, A. W.; Clark, J. Ultrafast Long-Range Charge Separation in Organic Semiconductor Photovoltaic Diodes. **2014**, *343*.
(39) Ji, L.-F.; Fan, J.-X.; Zhang, S.-F.; Ren, A.-M. Theoretical Investigations into the Charge Transfer Properties of Thiophene α-Substituted Naphthodithiophene Diimides: Excellent n-Channel and Ambipolar Organic Semiconductors. *Phys. Chem. Chem. Phys.* **2017**, *19* (21), 13978–13993. https://doi.org/10.1039/C7CP01114H.
(40) Sato, R.; Yoo, D.; Mori, T. 1:2 Charge-Transfer Complexes of Perylene and Coronene With Perylene Diimide, and the Ambipolar Transistors. *CrystEngComm* **2019**, *21* (20), 3218–3222. https://doi.org/10.1039/c9ce00456d.
(41) Matsumoto, T.; Urano, Y.; Takahashi, Y.; Mori, Y.; Terai, T.; Nagano, T. In Situ Evaluation of Kinetic Resolution Catalysts for Nitroaldol by Rationally Designed Fluorescence Probe. *Journal of Organic Chemistry* **2011**, *76* (10), 3616–3625. https://doi.org/10.1021/jo1020344.
(42) Freeman, D. M. E.; Tregnago, G.; Rodriguez, S. A.; Fallon, K. J.; Cacialli, F.; Bronstein, H. Deep-Red Electrophosphorescence from a Platinum(II)–Porphyrin Complex Copolymerised with Polyfluorene for Efficient Energy Transfer and Triplet Harvesting. *Journal of Organic Semiconductors* **2015**, *3* (1), 1–7. https://doi.org/10.1080/21606099.2015.1047473.
(43) Jones, B. A.; Facchetti, A.; Wasielewski, M. R.; Marks, T. J. Tuning Orbital Energetics in Arylene Diimide Semiconductors. Materials Design for Ambient Stability of n-Type Charge Transport. *Journal of the American Chemical Society* **2007**, *129* (49), 15259–15278. https://doi.org/10.1021/ja075242e.
(44) Panda, D. K.; Goodson, F. S.; Ray, S.; Lowell, R.; Saha, S. Multichromophoric Dye-Sensitized Solar Cells Based on Supramolecular Zinc-Porphyrin⋯perylene-Imide Dyads. *Chemical Communications* **2012**, *48* (70), 8775–8777. https://doi.org/10.1039/c2cc33120a.
(45) Dotcheva, D.; Klapper, M.; Müllen, K. Soluble Polyimides Containing Perylene Units. *Macromolecular Chemistry and Physics* **1994**, *195* (6), 1905–1911. https://doi.org/10.1002/macp.1994.021950602.

16. NMR Spectra

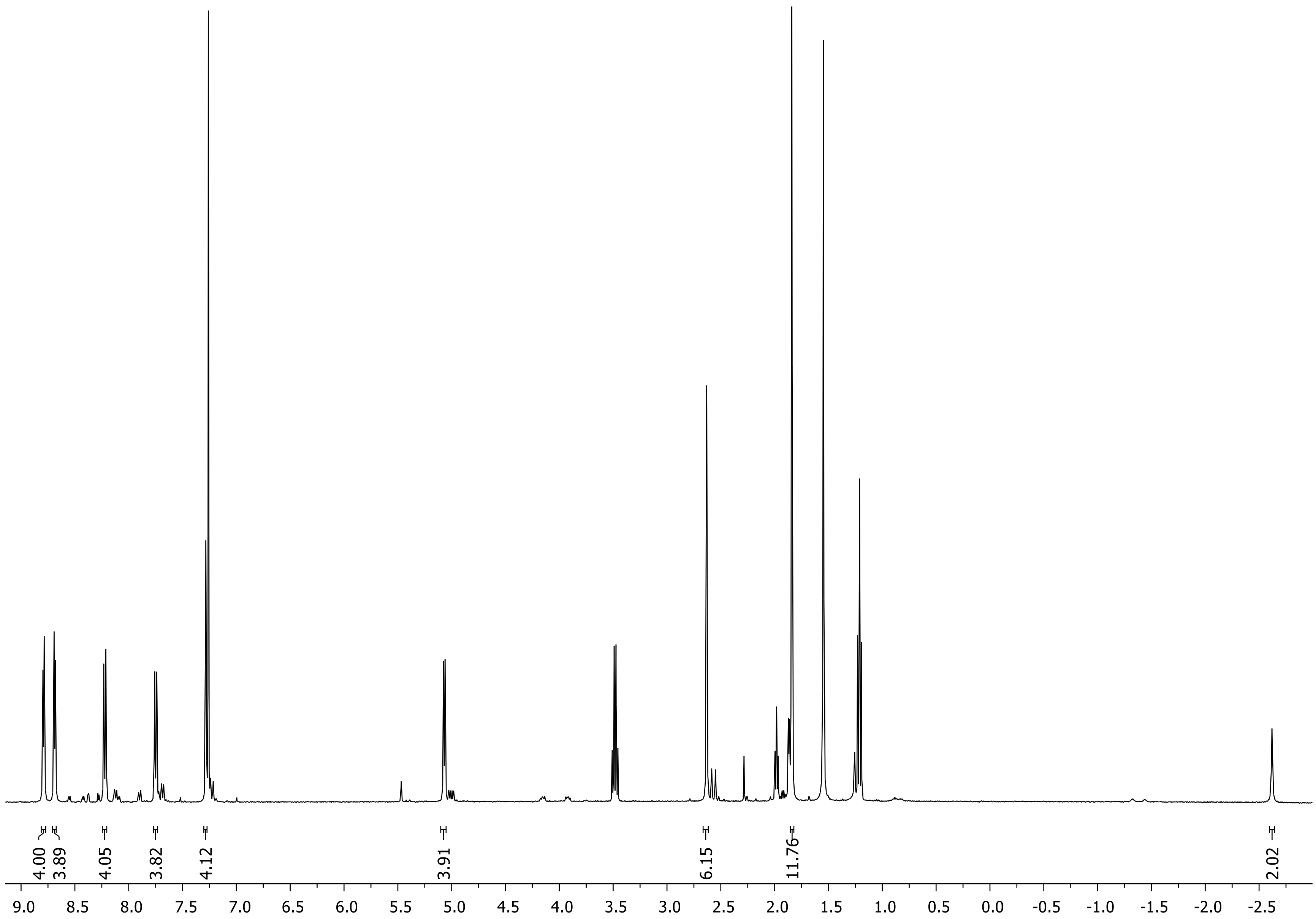

Spectrum S1. ^{1}H NMR spectrum of **9** as used in the subsequent step

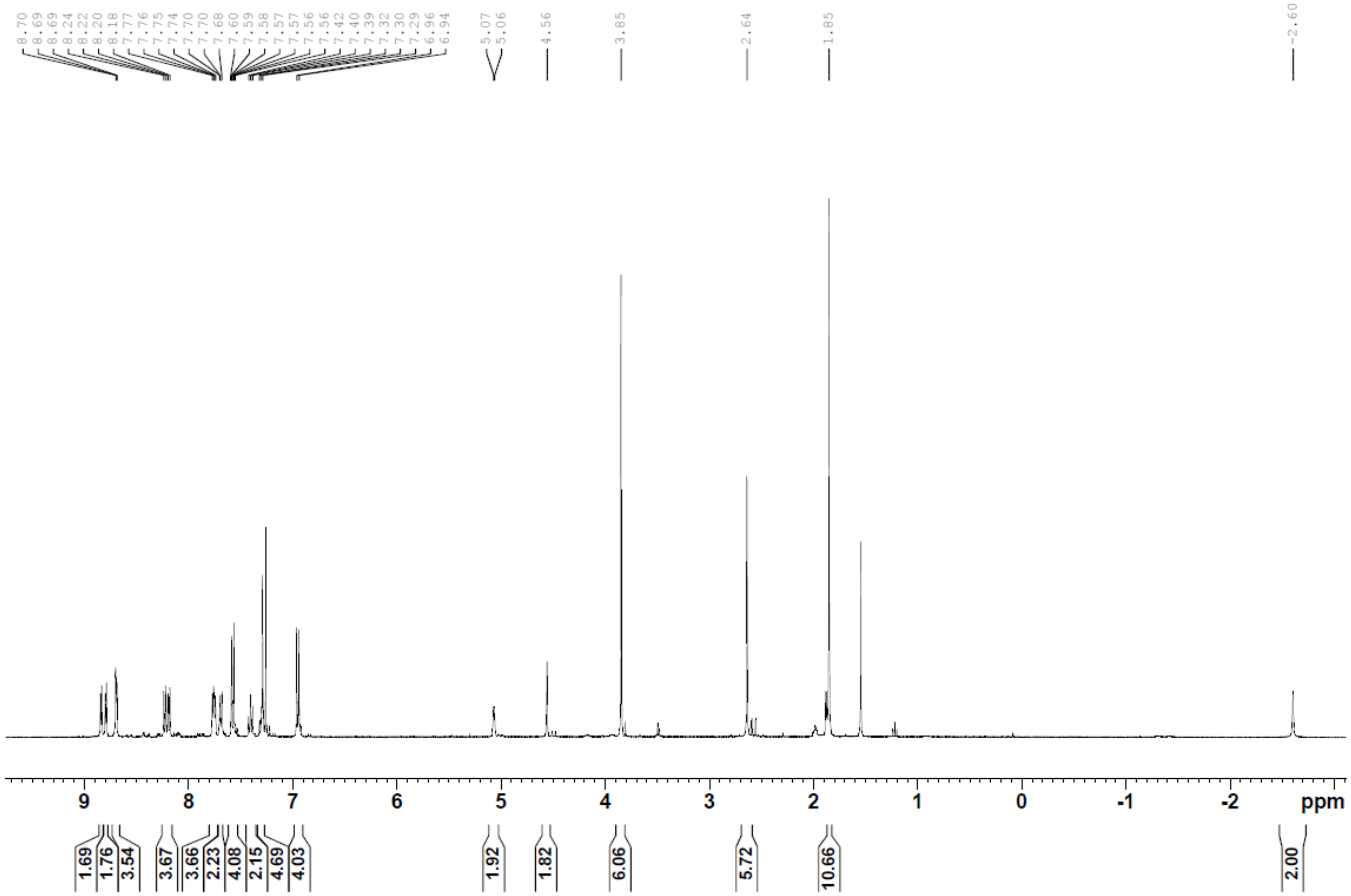

Spectrum S2. ^{1}H NMR spectrum of **3**.

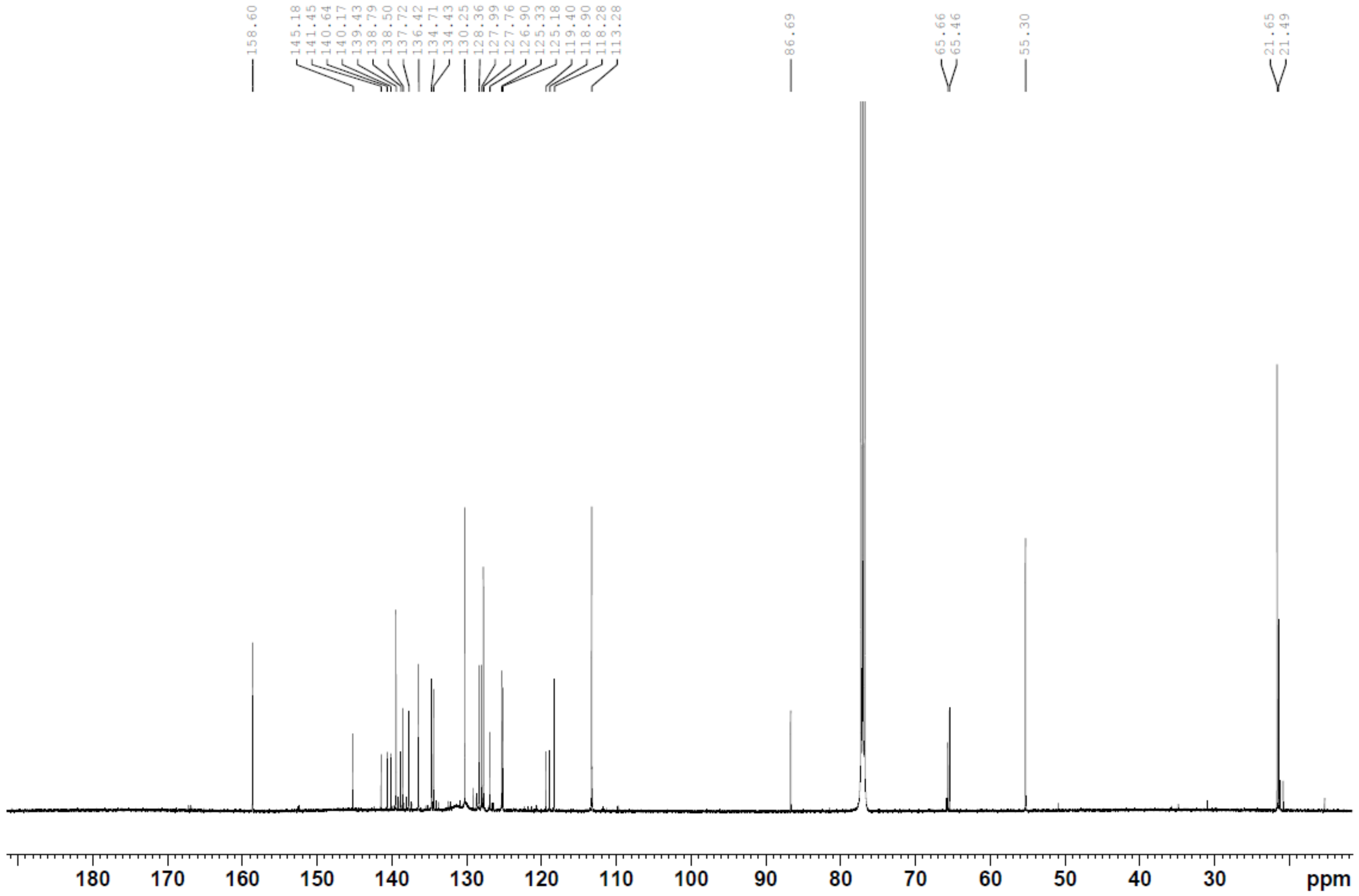

Spectrum S3. ^{13}C NMR spectrum of **3**

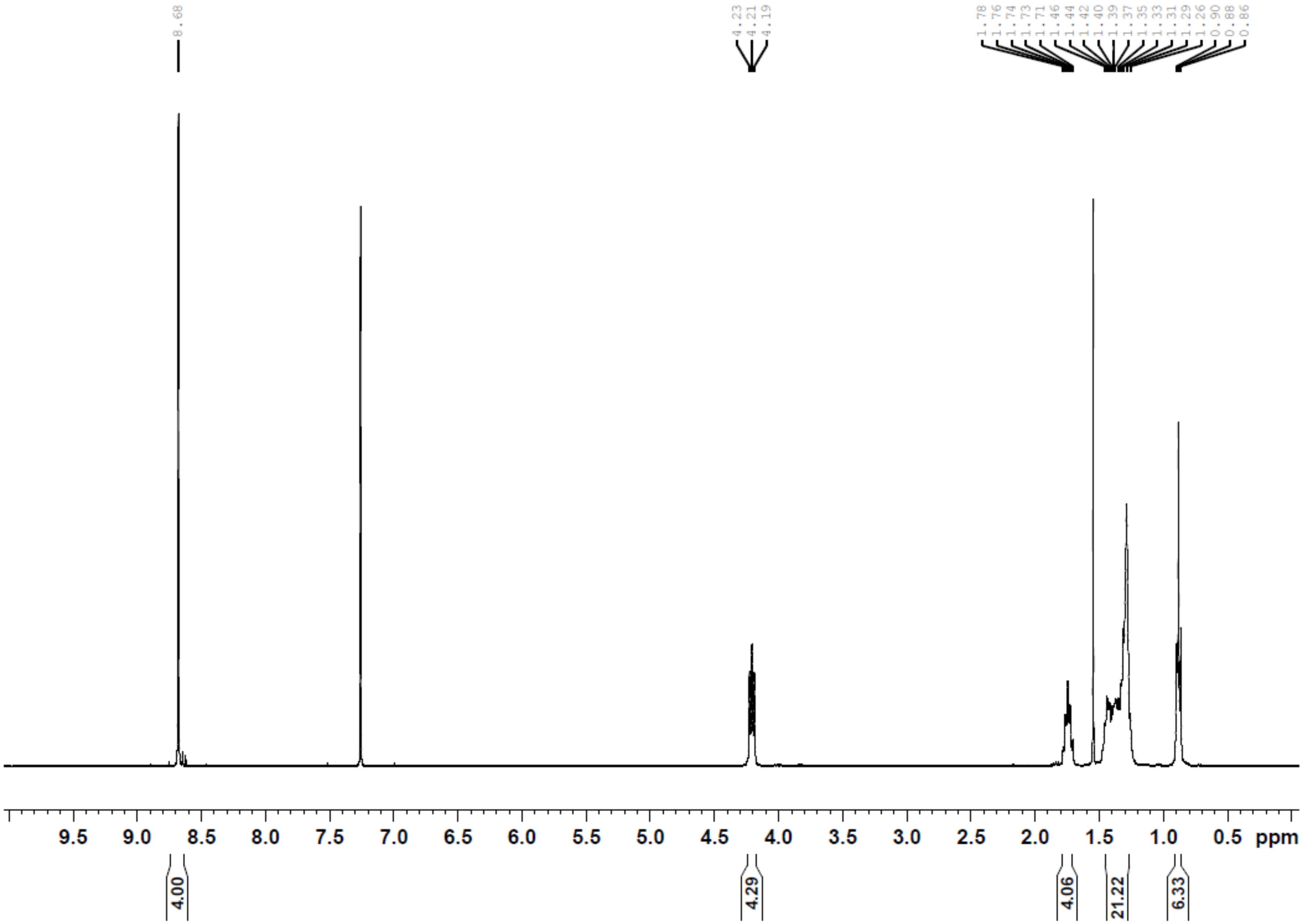

Spectrum S4. ^{1}H NMR spectrum of **6**

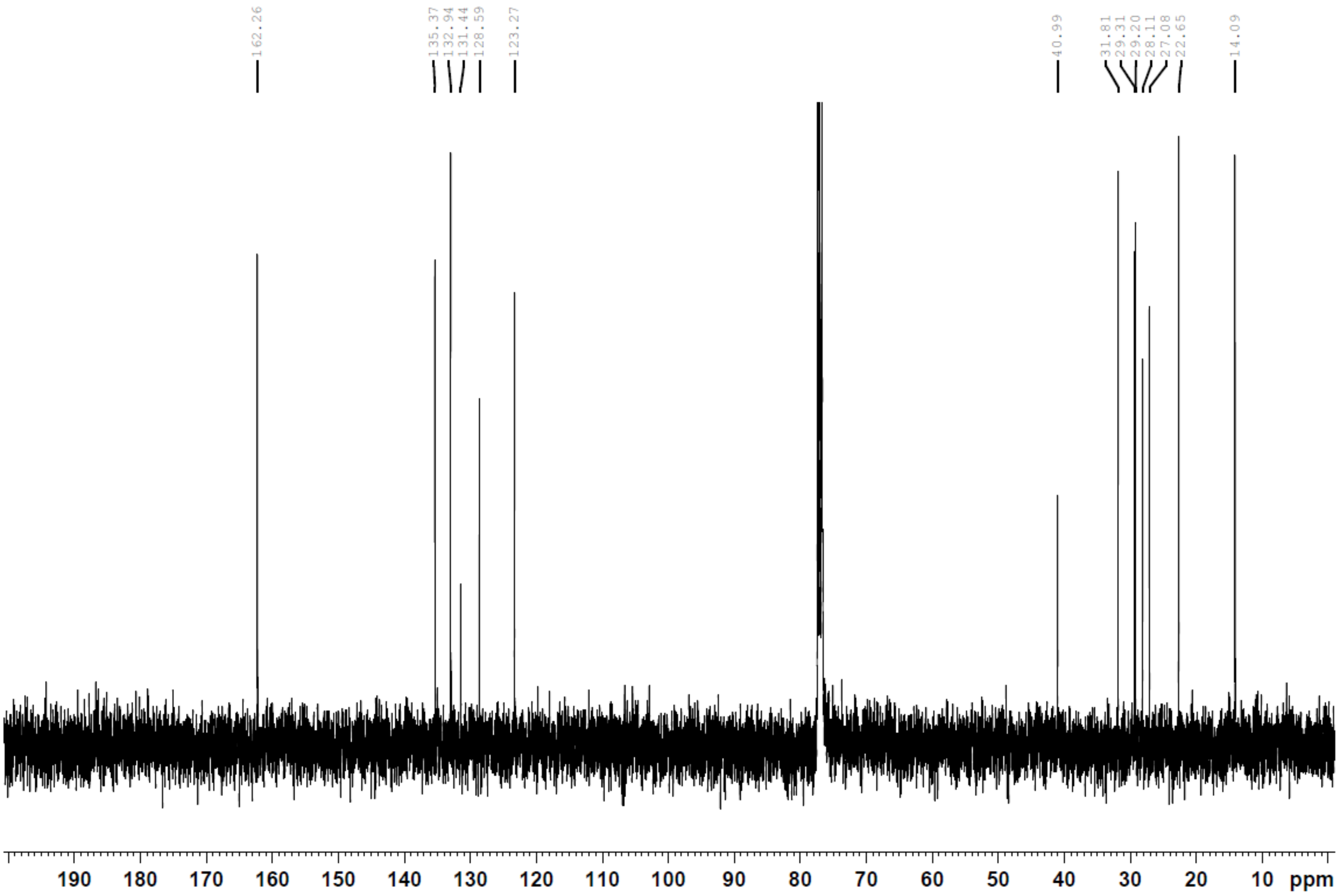

Spectrum S5. ^{13}C NMR spectrum of **6**

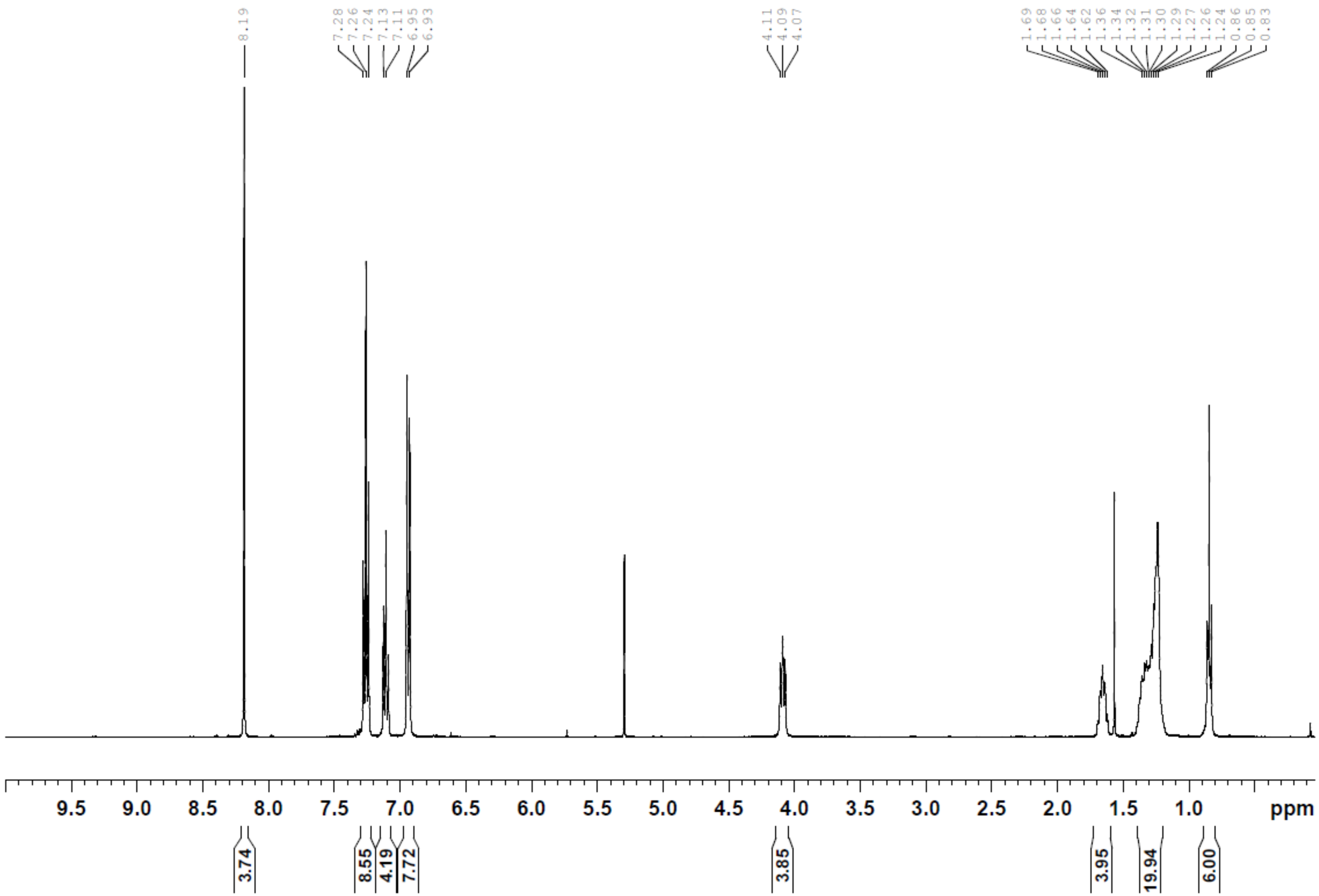

Spectrum S6. ^{1}H NMR spectrum of **7**

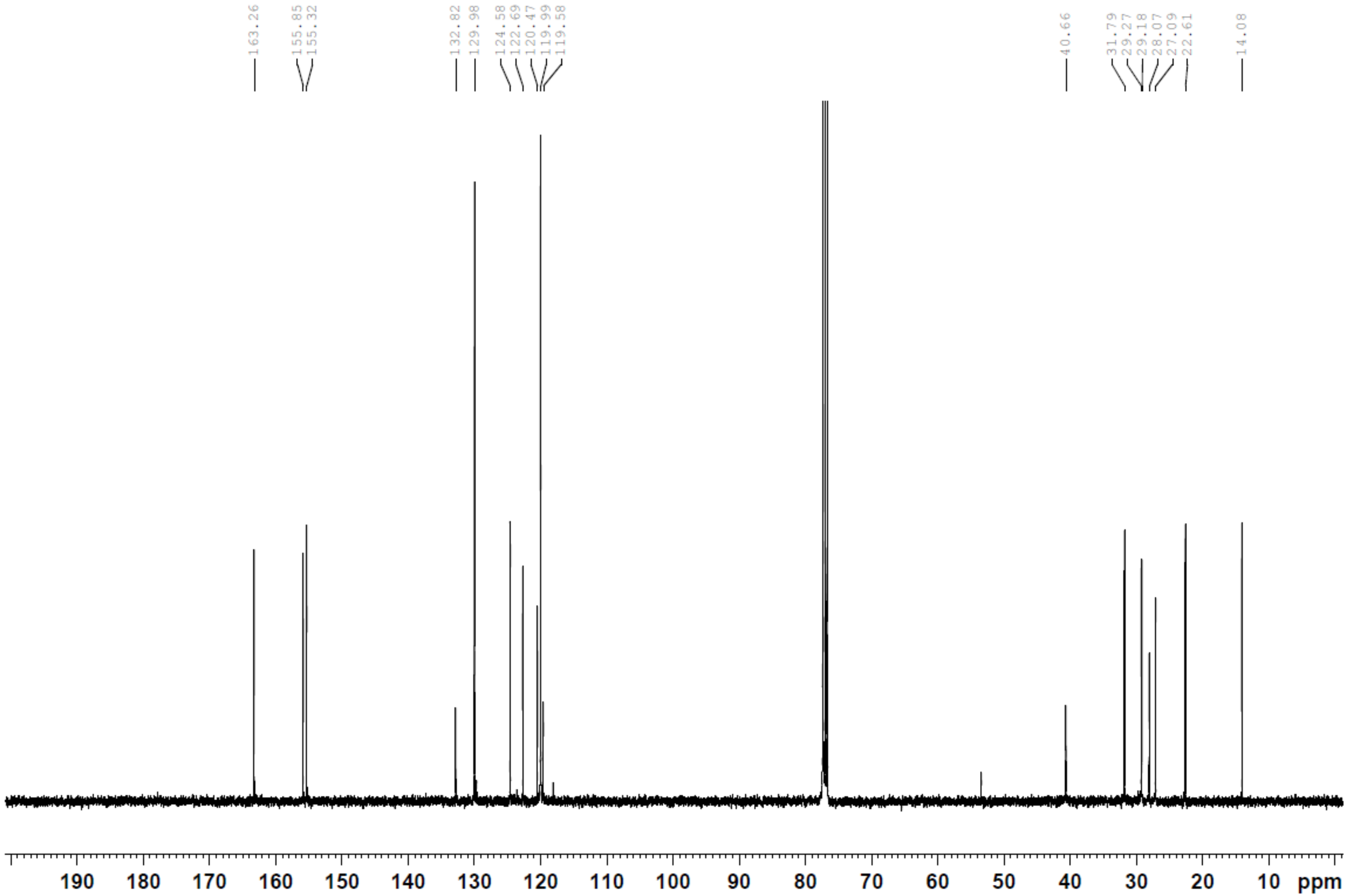

Spectrum S7. ^{13}C NMR spectrum of **7**

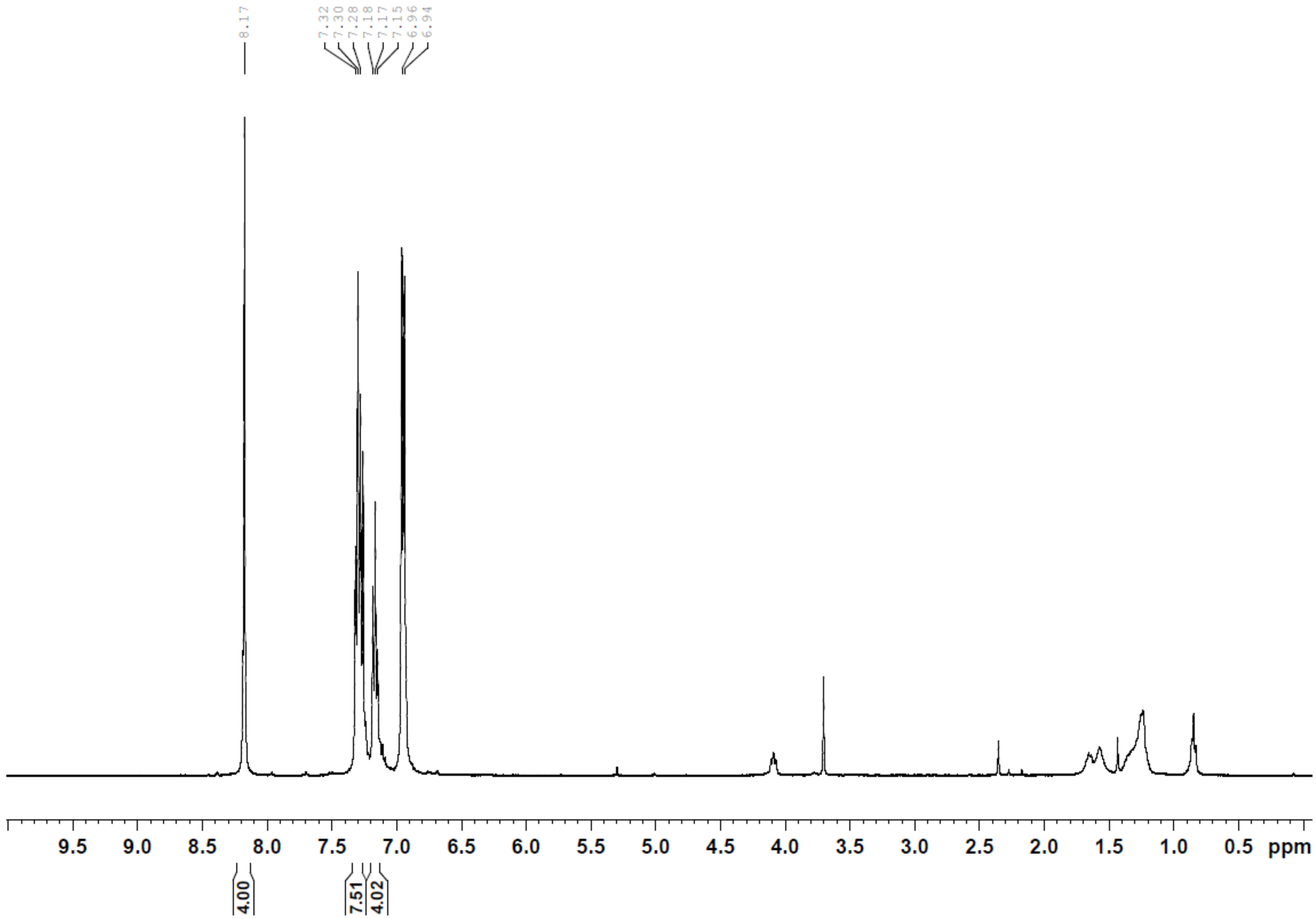

Spectrum S8. ^{1}H NMR spectrum of **8**

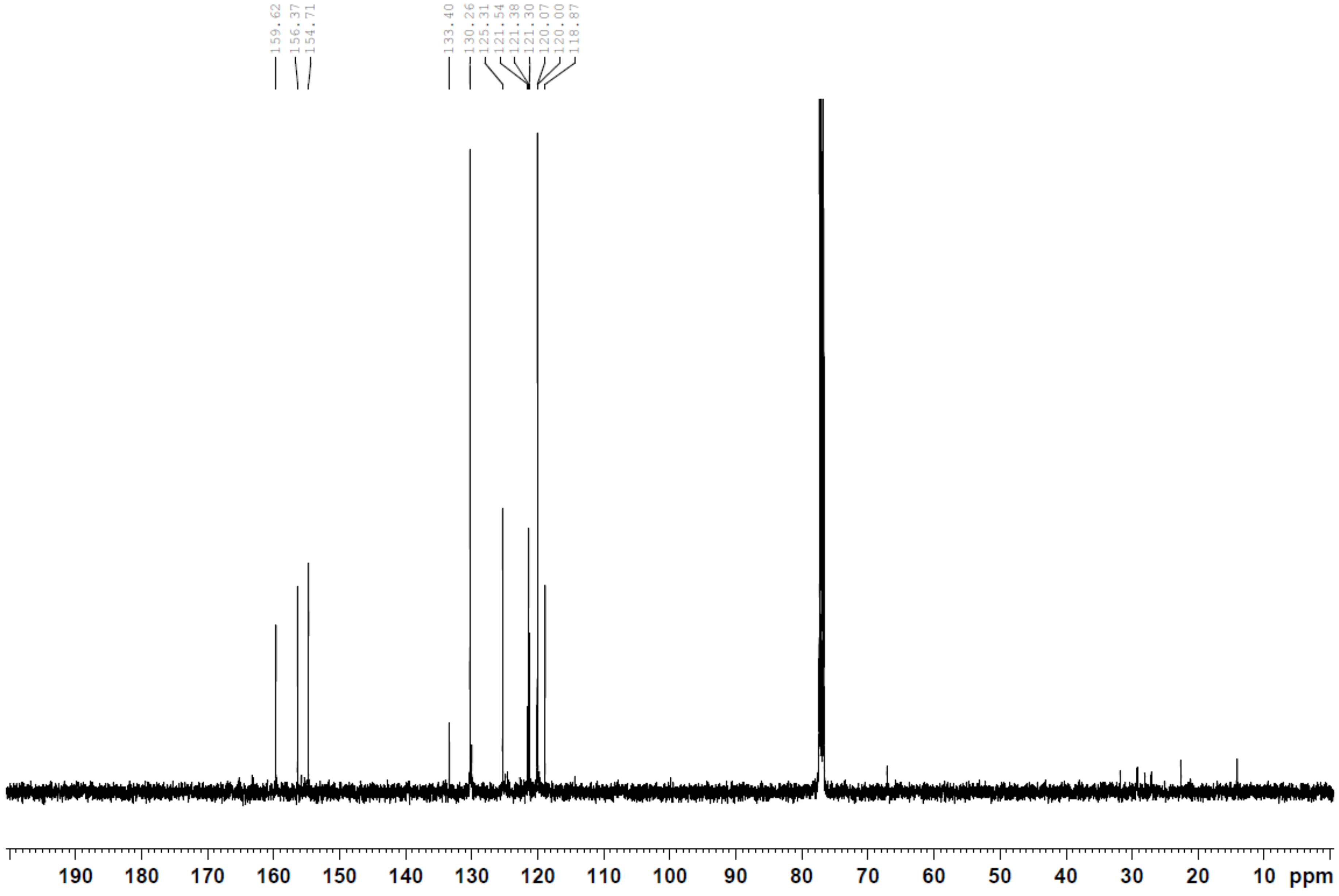

Spectrum S9. ^{13}C NMR spectrum of **8**

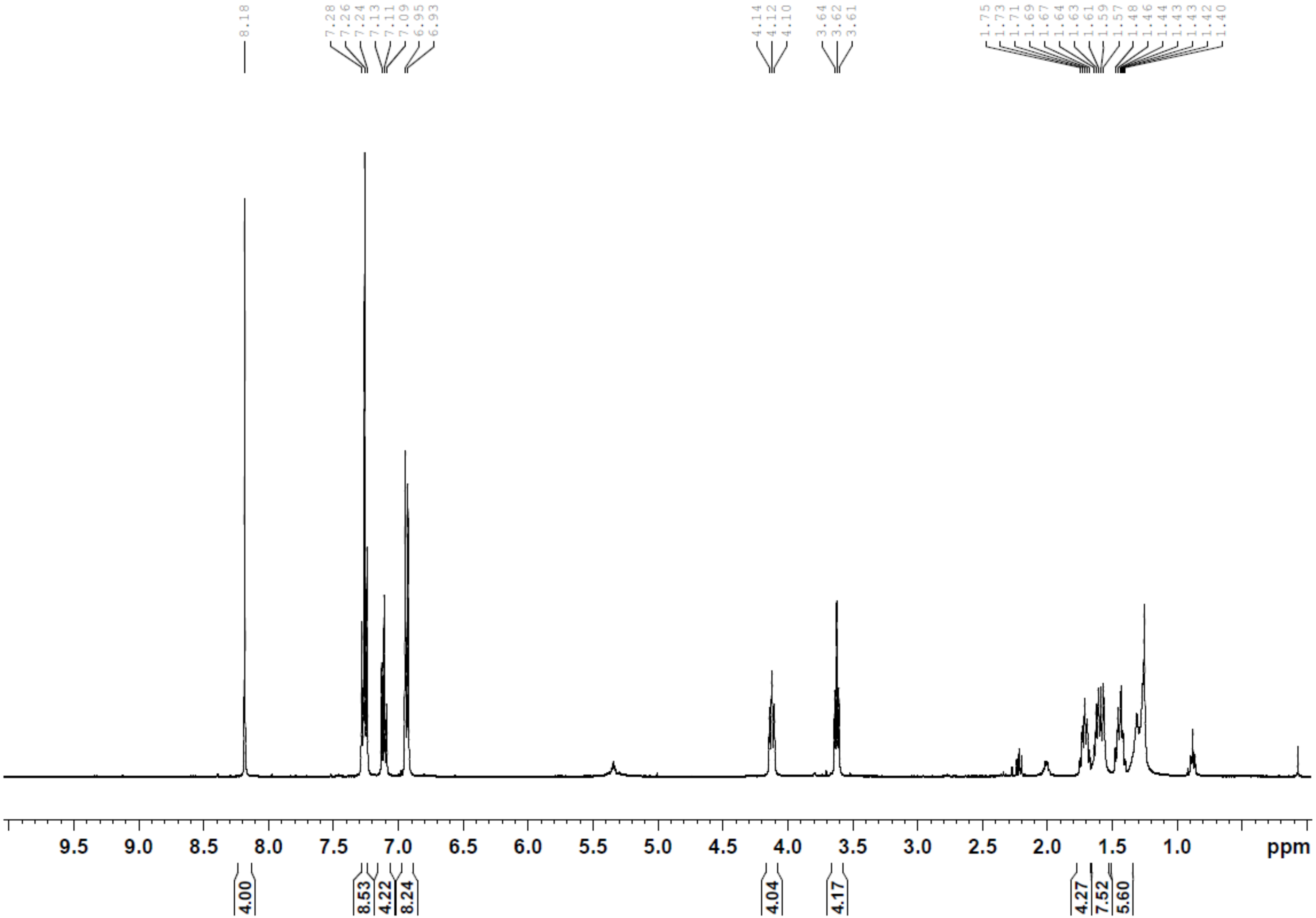

Spectrum S10. ^{1}H NMR spectrum of **9**

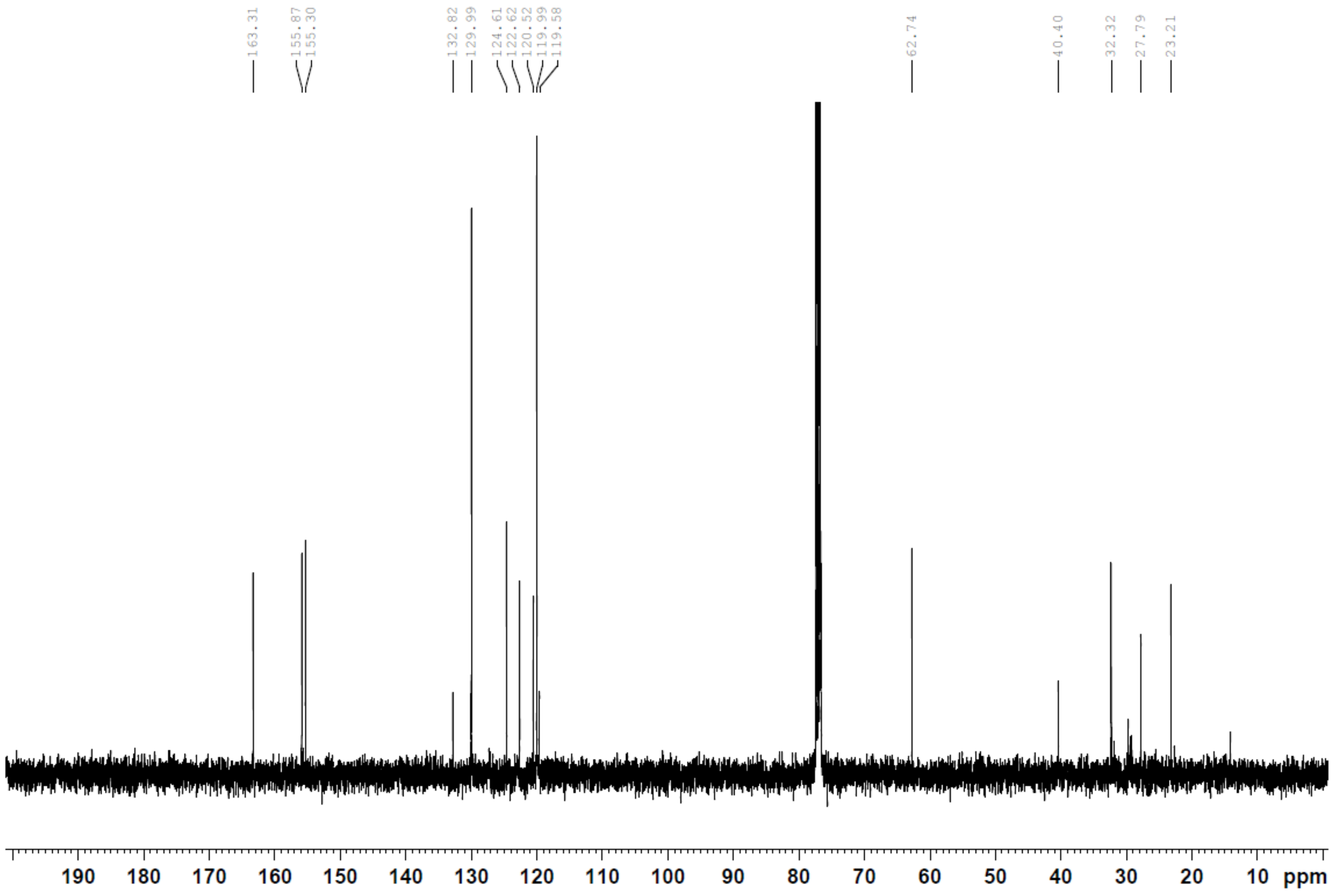

Spectrum S11. ^{13}C NMR spectrum of **9**

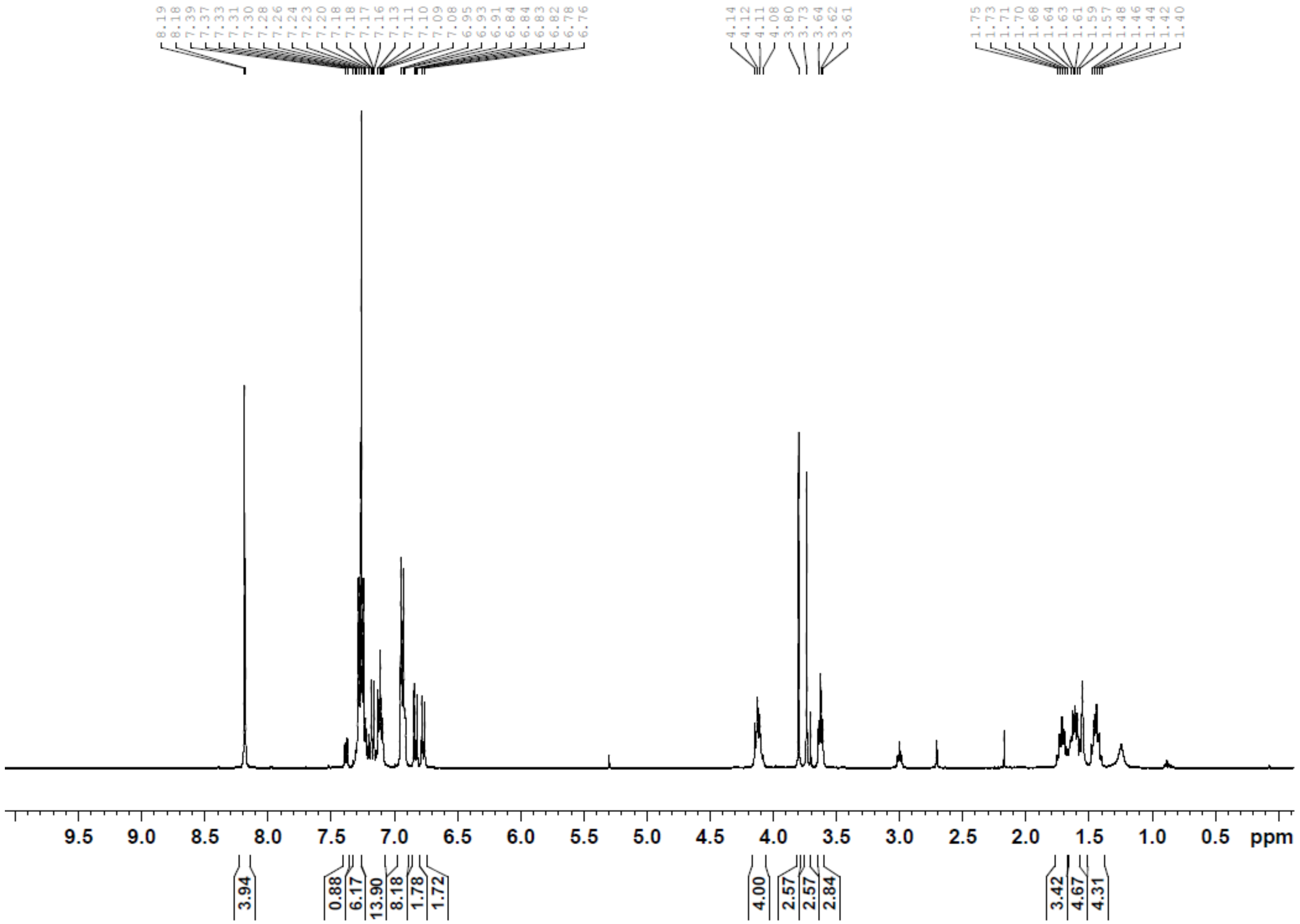

Spectrum S12. ^{1}H NMR spectrum of **10**

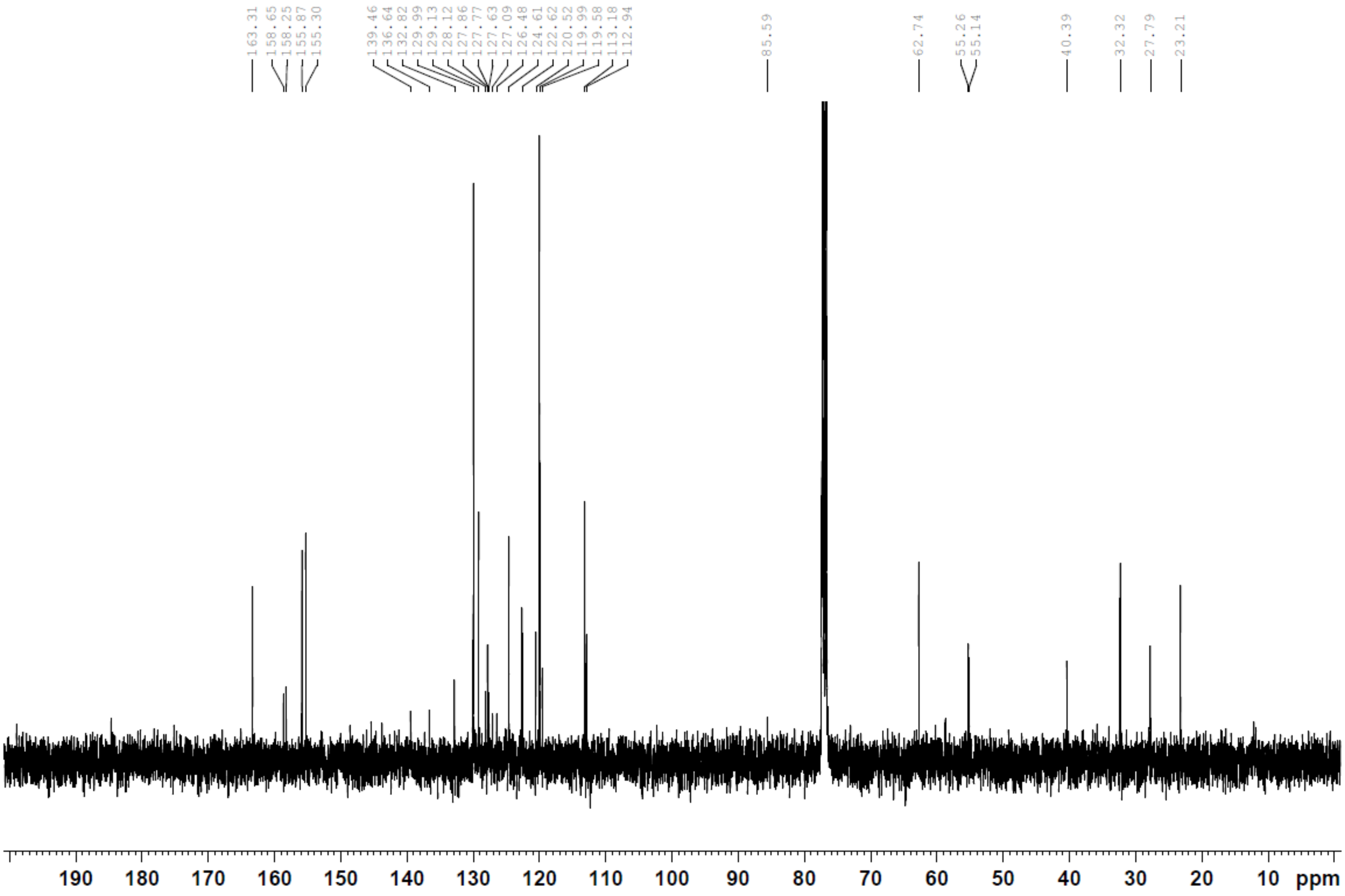

Spectrum S13. ^{13}C NMR spectrum of **1**
